# Dorsal Raphe Dopamine Neurons Represent the Experience of Social Isolation

**DOI:** 10.1016/j.cell.2015.12.040

**Published:** 2016-02-11

**Authors:** Gillian A. Matthews, Edward H. Nieh, Caitlin M. Vander Weele, Sarah A. Halbert, Roma V. Pradhan, Ariella S. Yosafat, Gordon F. Glober, Ehsan M. Izadmehr, Rain E. Thomas, Gabrielle D. Lacy, Craig P. Wildes, Mark A. Ungless, Kay M. Tye

**Affiliations:** 1 The Picower Institute for Learning and Memory, Department of Brain and Cognitive Sciences, Massachusetts Institute of Technology, Cambridge, MA 02139, USA; 2 Medical Research Council Clinical Sciences Centre, Imperial College London, Hammersmith Hospital, Du Cane Road, W12 0NN London, UK

## Abstract

The motivation to seek social contact may arise from either positive or negative emotional states, as social interaction can be rewarding and social isolation can be aversive. While ventral tegmental area (VTA) dopamine (DA) neurons may mediate social reward, a cellular substrate for the negative affective state of loneliness has remained elusive. Here, we identify a functional role for DA neurons in the dorsal raphe nucleus (DRN), in which we observe synaptic changes following acute social isolation. DRN DA neurons show increased activity upon social contact following isolation, revealed by in vivo calcium imaging. Optogenetic activation of DRN DA neurons increases social preference but causes place avoidance. Furthermore, these neurons are necessary for promoting rebound sociability following an acute period of isolation. Finally, the degree to which these neurons modulate behavior is predicted by social rank, together supporting a role for DRN dopamine neurons in mediating a loneliness-like state.

**PaperClip:**

## Highlights

• Dorsal raphe nucleus (DRN) dopamine neurons are sensitive to acute social isolation
• DRN dopamine neurons release dopamine and glutamate in downstream structures
• Optical activation induces, whereas inhibition suppresses, a “loneliness-like” state
• Social rank predicts the behavioral effect induced by optical manipulations

## Graphical Abstract

**Figure:**
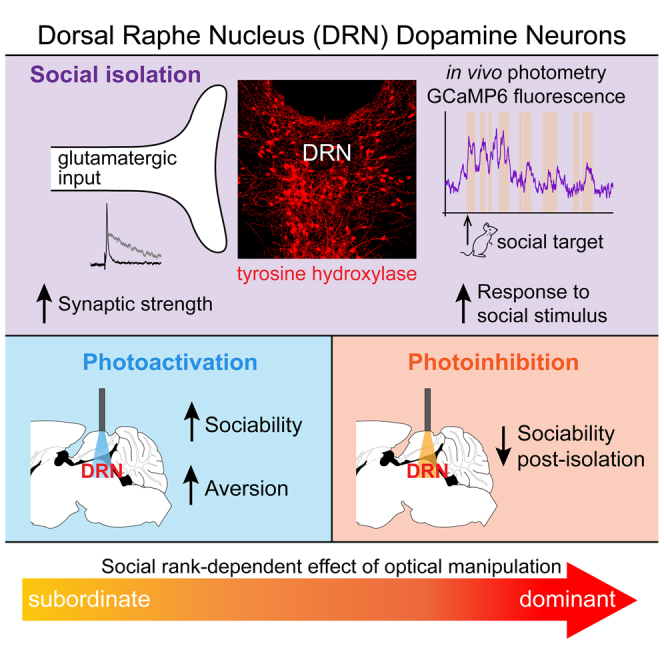

## Teaser

Dopamine neurons within the dorsal raphe nucleus are sensitive to acute social isolation, and are able to modulate a “loneliness-like” state upon optical stimulation. These neurons may underlie the subjective experience of social isolation as well as the motivational drive to re-engage in social connections.

## Introduction

The establishment and maintenance of social bonds is crucial for survival of a social species. A social group offers safety and security, supports offspring survival, reduces the need for energy expenditure, and provides a stage for social reward (Eisenberger, 2012). The motivation to initiate and maintain social bonds may be rooted in emotional states of either positive or negative valence. Social interactions can be rewarding and thereby recruit components of the brain’s reward circuitry, including the ventral tegmental area (VTA) dopamine (DA) neurons and the nucleus accumbens (NAc) (Dölen et al., 2013, Gunaydin et al., 2014, Robinson et al., 2002).

Conversely, the absence of social contact also triggers a strong desire to seek social interaction. Social isolation, social exclusion, or feelings of social disconnection can lead to loneliness, which is a strongly aversive emotional state in humans and detrimental to physical and mental well-being (Cacioppo et al., 2006, Cacioppo et al., 2014, Holt-Lunstad et al., 2010, House et al., 1988). The aversive nature of this state is emphasized by the controversial use of solitary confinement as a form of punishment (Browne et al., 2011, Walker et al., 2014). Therefore, the negative state of isolation can trigger the motivation to seek and engage in social contact (Baumeister and Leary, 1995, Maner et al., 2007, Williams and Sommer, 1997), perhaps as an evolutionarily conserved mechanism to maintain social connections (Buss, 1990).

Social isolation is also aversive to rodents. Rodents are innately social creatures and prefer social rather than isolate housing (Loo et al., 2001). Even an acute period of isolation in rodents increases motivation to seek out and engage with conspecifics (Niesink and van Ree, 1982, Panksepp and Beatty, 1980). However, little is known about how this isolation-induced state is represented at a neural level.

Given that the mesolimbic DA system has been implicated in social behavior (Gunaydin et al., 2014, Puglisi-Allegra and Cabib, 1997, Robinson et al., 2002) and that perturbations in DA signaling have been reported following chronic social isolation (Hall et al., 1998), we initially considered VTA DA neurons as a candidate neural substrate for social isolation. However, since optogenetic activation of VTA DA neurons increases social interaction (Gunaydin et al., 2014) and supports positive reinforcement (Tsai et al., 2009, Witten et al., 2011), they are thought to play a causal role in social reward.

Given that DA neurons are functionally heterogeneous (Brischoux et al., 2009, Lammel et al., 2012), we investigated a relatively neglected subpopulation of DA neurons in the dorsal raphe nucleus (DRN). Amid the sparse existing knowledge of the functional role of DRN DA neurons, optical stimulation of these neurons does not support intra-cranial self-stimulation (ICSS) (McDevitt et al., 2014), in contrast to the VTA (Witten et al., 2011), suggesting that DRN and VTA DA neurons may be functionally distinct.

Here, we investigated the functional role of DRN DA neurons, which we found to possess the properties expected of a neural substrate for a “loneliness-like” state. Specifically, the strength of excitatory inputs onto DRN DA neurons and their naturally occurring activity in vivo were sensitive to social isolation. Optical activation of these neurons recapitulated a loneliness-like state, while optical inhibition prevented the sociability typically observed following a period of isolation. Furthermore, the magnitude of these effects was predicted by an individual’s social rank, which indicates the importance of prior social experience in determining the behavioral effect governed by these neurons. Taken together, we propose that DRN DA neurons represent a neural substrate for the subjective experience of social isolation and serve to promote a response to alleviate this aversive state.

## Results

### Acute Social Isolation Potentiates Synapses onto DA Neurons in the DRN, but Not the VTA

**Figure 1:**
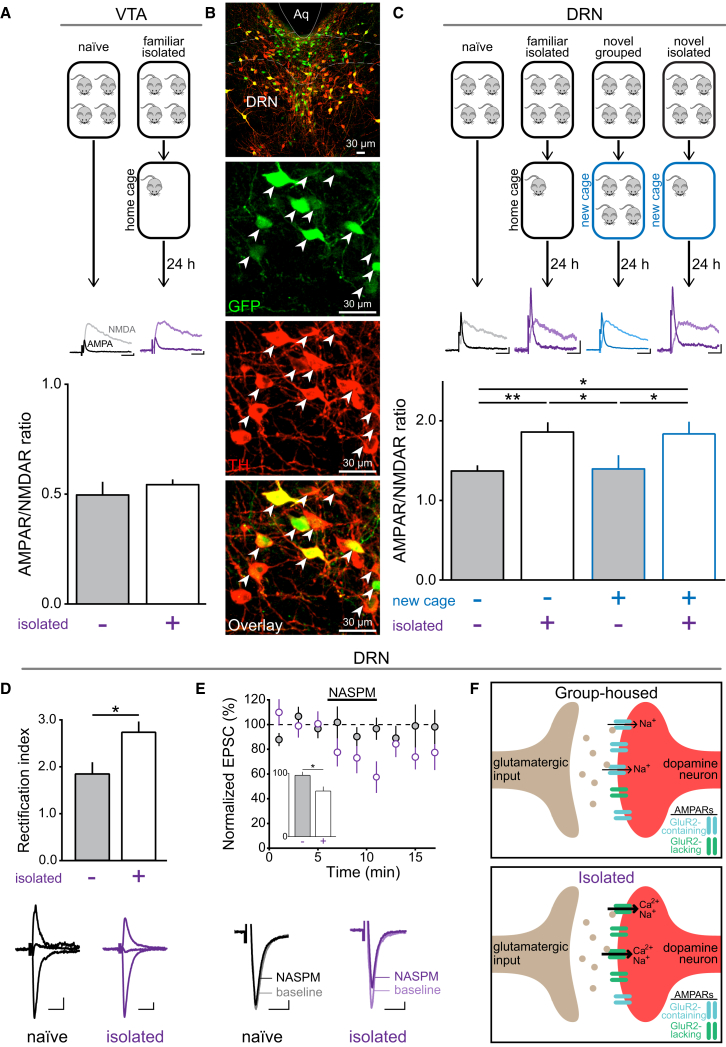
24 Hours of Social Isolation Induces Synaptic Potentiation onto DRN DA Neurons (A) AMPAR/NMDAR ratios recorded from VTA DA neurons in mice socially isolated for 24 hr (n = 12) were not significantly different from group-housed mice (n = 8; unpaired t test: t_18_ = 0.73, p = 0.47). (B) Low- (upper panel) and high-magnification (lower panels) confocal images of the DRN from a TH-GFP mouse showing GFP-expressing (green) and post hoc immunohistochemically verified TH-expressing (red) DA neurons with white arrows indicating co-labeled neurons. (C) AMPAR/NMDAR ratios recorded from DRN DA neurons in mice socially isolated for 24 hr, either in a familiar cage or a novel cage (familiar isolated or novel isolated, respectively), were significantly greater than group-housed mice in familiar or novel cages (one-way ANOVA: F_3,47_ = 5.910, ^∗∗^p = 0.0017; Newman-Keuls post hoc tests: ^∗^p < 0.05, ^∗∗^p < 0.01; n = 19 naive, 17 familiar isolated, 9 novel grouped, and 6 novel isolated). Scale bars, 20 pA, 20 ms. (D) The AMPAR rectification index in DRN DA neurons was significantly greater in socially isolated mice, relative to naive mice (unpaired t test: t_21_ = 2.417, ^∗^p = 0.0248, n = 9 naive, 14 isolated). (E) Normalized AMPAR-mediated EPSC amplitude during bath application of NASPM, and representative averaged EPSCs from a naive and socially isolated mouse (inset shows % change in EPSC amplitude following NASPM, relative to baseline). NASPM significantly reduced EPSC amplitude in socially isolated mice (n = 7), relative to naive mice (n = 8; unpaired t test: t_13_ = 2.853, ^∗^p = 0.0136). Scale bars, 10 pA, 10 ms. (F) Proposed model of AMPARs at synapses onto DRN DA neurons in group-housed mice and following social isolation. Data are represented as mean ± SEM. See also Figure S1.

**Figure S1:**
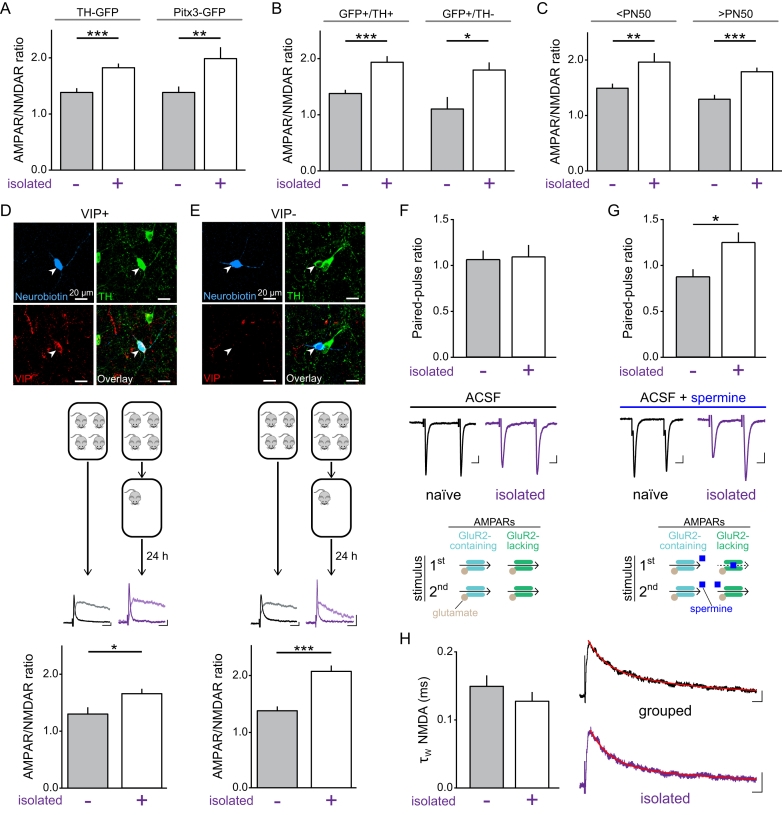
Electrophysiological Measures of AMPAR and NMDAR Current Properties in DRN DA Neurons of Group-Housed and Socially Isolated Mice, Related to Figure 1 (A) AMPAR/NMDAR ratios recorded in DRN DA neurons from both TH-GFP and Pitx3-GFP mice showed a significant increase following 24 hr social isolation in comparison to group-housed mice (TH-GFP – unpaired t test: t_48_ = 4.100, ^∗∗∗^p = 0.0002; n = 25 grouped, 25 isolated; Pitx3-GFP – unpaired t test: t_27_ = 2.813, ^∗∗^p = 0.0090; n = 17 grouped, 12 isolated). (B) AMPAR/NMDAR ratios recorded from GFP+/TH+ and GFP+/TH- DRN neurons, in both transgenic mouse lines, were significantly greater in socially isolated compared with group-housed mice (GFP+/TH+: unpaired t test: t_46_ = 4.149, ^∗∗∗^p = 0.0001; n = 23 grouped, 25 isolated; GFP+/TH-: unpaired t test: t_8_ = 2.695, ^∗^p = 0.0273; n = 5 grouped, 5 isolated). (C) Both adolescent (< PN50) and adult (> PN50) mice showed a significant increase in the AMPAR/NMDAR ratio following social isolation (< PN50 – unpaired t test: t_40_ = 3.011, ^∗∗^p = 0.0045; n = 23 grouped, 19 isolated; ≥ PN50 – unpaired t test: t_33_ = 4.226, ^∗∗∗^p = 0.0002; n = 18 grouped, 17 isolated). (D and E) Example high-magnification confocal images of neurobiotin-filled (blue) neurons immunohistochemically identified as TH+ (green) and (D) VIP+ (red) or (E) VIP−. Illustration of mouse housing manipulations, example AMPAR/NMDAR ratios recorded in DRN DA neurons, and bar charts showing the mean ratio for each condition. Both VIP+ and VIP− DRN DA neurons showed a significantly greater AMPAR/NMDAR ratio following social isolation (VIP+: unpaired t test: t_18_ = 2.210, ^∗^p = 0.0403; n = 10 grouped, 10 isolated; VIP−: unpaired t test: t_36_ = 4.78, ^∗∗∗^p < 0.0001; n = 18 grouped, 20 isolated). Scale bars, 20 pA, 20 ms. (F) In ACSF, the AMPAR PPR was not significantly different between naive (n = 14) and socially isolated mice (n = 11; unpaired t test: t_23_ = 0.0441, p = 0.952). (G) In the presence of the polyamine spermine, the AMPAR PPR was significantly greater in socially isolated relative to naive mice (unpaired t test: t_31_ = 2.269, ^∗^p = 0.0304, n = 11 naive, 22 isolated). Scale bars, 10 pA, 10 ms. (H) The decay phase of the NMDAR current was fitted with a double exponential function to calculate the weighted decay time constant (τ_W_). No significant difference was found in the decay time constant of the NMDAR current between group-housed and socially isolated mice (Mann-Whitney U = 248, p = 0.7002, n = 28 grouped, 19 isolated). Scale bars, 20 pA, 20 ms. Data are represented as mean + SEM.

In order to probe the effect of social isolation on glutamatergic synaptic strength, we used whole-cell patch-clamp electrophysiology in brain slices prepared from male mice expressing GFP in DA neurons (Figures S1A–S1E; Supplemental Experimental Procedures) and measured the α-amino-3-hydroxy-5-methyl-4-isoxazolepropionic acid receptor (AMPAR)/N-methyl-D-aspartate receptor (NMDAR) ratio. Glutamatergic synapses onto VTA DA neurons undergo rapid changes in synaptic strength within 24 hr of an acute appetitive (Ungless et al., 2001) or aversive experience (Lammel et al., 2011, Saal et al., 2003). We therefore considered whether 24 hr of social isolation could induce potentiation at these synapses. However, we did not detect a difference in AMPAR/NMDAR ratio between group-housed and socially isolated mice in VTA DA neurons (Figure 1A).

Among the relatively unexplored subpopulations of DAergic neurons residing outside of the VTA, an intriguing group lies within the DRN (Figure 1B) (Hökfelt et al., 1976), which is highly conserved across species (Saper and Petito, 1982). Strikingly, DRN DA neurons in mice that were socially isolated for 24 hr exhibited a significantly greater AMPAR/NMDAR ratio than group-housed, naive mice (Figure 1C). In order to confirm this effect was related to the experience of social isolation, rather than a nonspecific salient environmental manipulation, we also examined movement into a new cage. We found that transfer into a new cage as a group had no detectable effect on AMPAR/NMDAR ratio, but social isolation in a new cage also increased the AMPAR/NMDAR ratio (Figure 1C).

### Social Isolation Changes Receptor Composition at Synapses onto DRN DA Neurons

To examine the mechanism of social isolation-induced plasticity in DRN DA neurons, we used additional electrophysiological measures. Postsynaptically, AMPAR subunit composition can influence neuron excitability and synaptic efficacy (Liu and Zukin, 2007). In contrast to GluR2-containing AMPARs, GluR2-lacking receptors are Ca^2+^-permeable and exhibit higher single-channel conductance (Hollmann et al., 1991, Swanson et al., 1997). We found that the rectification index (RI) of the AMPAR current was significantly greater in socially isolated mice (Figure 1D), suggesting an increase in GluR2-lacking AMPARs, which exhibit a characteristic inwardly rectifying current at positive potentials (Bellone and Lüscher, 2006, Liu and Zukin, 2007). At resting membrane potentials, the polyamine spermine can partially block GluR2-lacking AMPARs (Bowie and Mayer, 1995), but opening of the receptor temporarily relieves this blockade, which results in a greater response to subsequent stimulation (Rozov and Burnashev, 1999). This therefore promotes paired-pulse facilitation at GluR2-lacking synapses (Liu and Zukin, 2007). Indeed, we found that the paired-pulse ratio (PPR) in the presence of spermine was greater in socially isolated mice (Figures S1F and S1G). Contrastingly, we did not detect a significant difference in decay time constant of the NMDAR current between group-housed and socially-isolated mice (Figure S1H). Finally, to confirm an increase in GluR2-lacking AMPARs at these synapses, we applied 1-naphthyl acetyl spermine (NASPM, a selective blocker of GluR2-lacking AMPARs) to brain slices prepared from group-housed or socially isolated mice. This reduced the amplitude of the evoked AMPAR current recorded in DRN DA neurons from socially isolated, but not group-housed, mice (Figure 1E). Taken together, this suggests that social isolation induces a relative increase in GluR2-lacking AMPARs at glutamatergic synapses onto DRN DA neurons (Figure 1F).

### Activity of DRN DA Neurons In Vivo Increases upon Initial Social Contact after Social Isolation

**Figure 2:**
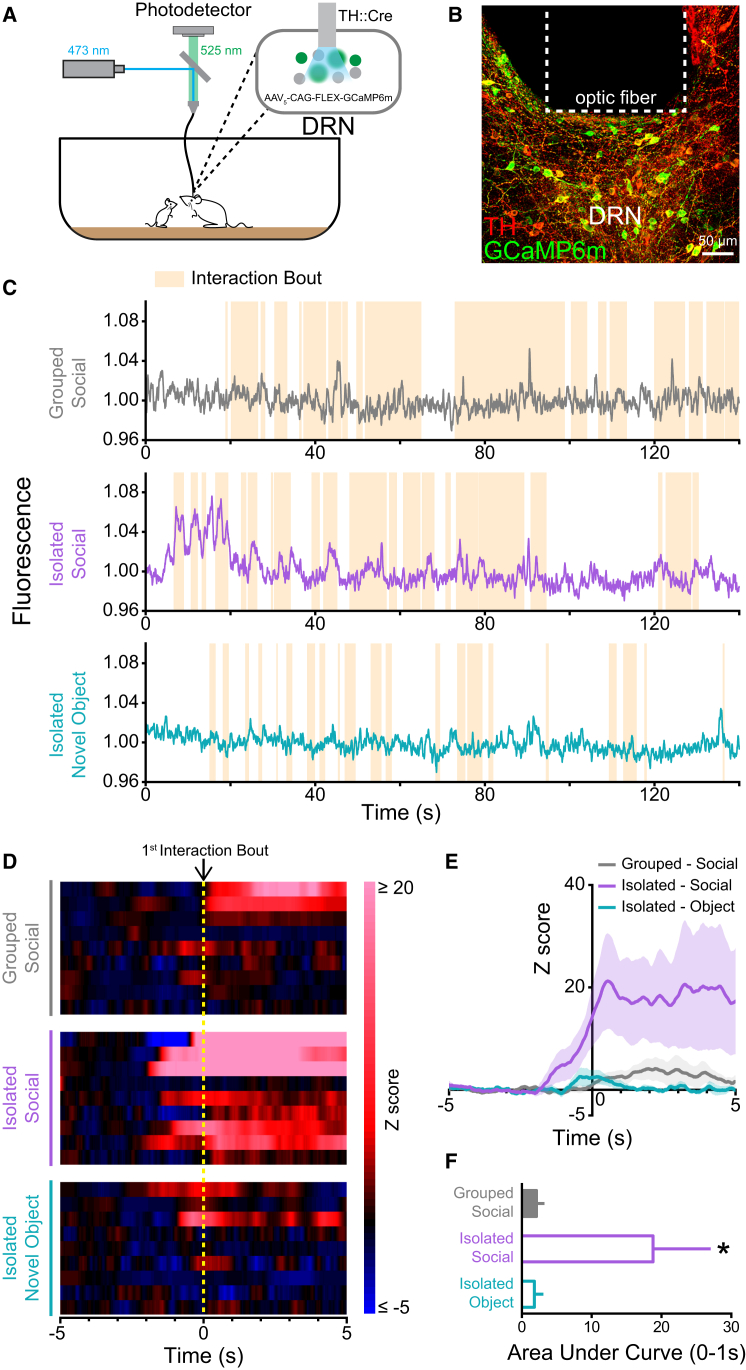
DRN DA Neurons Track Initial Social Contact Following Social Isolation (A) Schematic for recording activity of GCaMP6m-expressing neurons. (B) Image showing Cre-dependent expression of GCaMP6m in the DRN of a TH::Cre mouse, with optic fiber placement indicated. (C) Representative traces of bulk fluorescence signal from DRN DA neurons, with shaded areas indicating interaction bouts. Mice were recorded under three conditions: group-housed mice presented with a juvenile mouse (gray), socially isolated mice presented with a juvenile mouse (lilac), or socially isolated mice presented with a novel object (teal). (D) Heat maps showing the individual Z scores in response to the first interaction bout for each animal under each condition. (E) Population Z score plots showing the averaged response to the first interaction bout. (F) DRN DA neurons in socially isolated mice showed a significantly greater increase in activity upon first contact with the juvenile mouse, compared with group-housed mice or response to a novel object (n = 9; one-way ANOVA: F_2,16_ = 4.978, ^∗^p = 0.0208; Bonferroni post hoc analysis: ^∗^p < 0.05 for both comparisons). Data are represented as mean ± SEM. See also Figure S2 and Movie S1.

**Figure S2:**
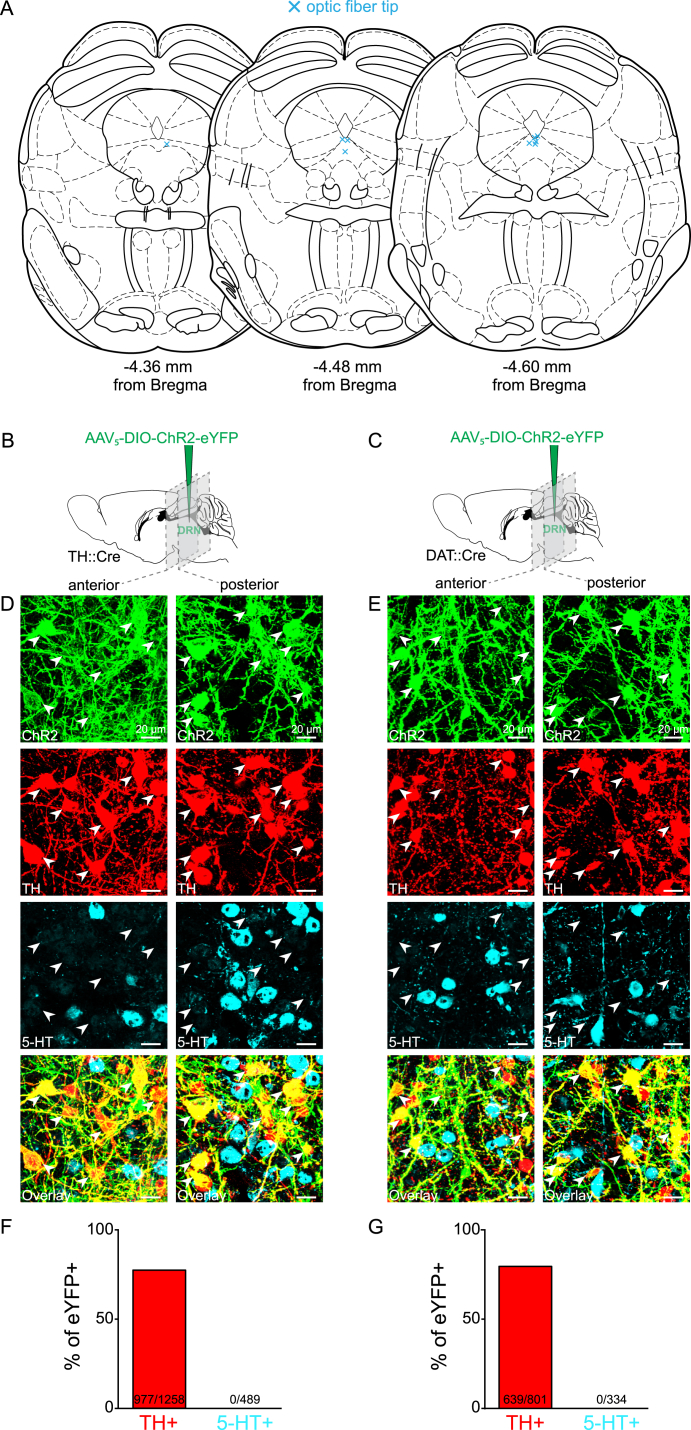
Placement of Optic Fiber in the DRN for Photometry Recordings and Immunohistochemical Characterization of ChR2-eYFP Expression in TH::Cre and DAT::Cre Mice, Related to Figures 2 and 3 (A) The position of the tip of the optic fiber (blue cross) in the DRN of TH::Cre mice expressing GCaMP6m used for photometry recordings. (B) TH::Cre and (C) DAT::Cre mice received an injection of AAV_5_-DIO-ChR2-eYFP into the DRN and coronal brain sections were prepared after at least 4 weeks of expression. (D) High-magnification confocal images of eYFP+ neurons, within one anterior and one posterior section of the DRN, showing co-expression of eYFP with TH (red) but not 5-HT (cyan) in a TH::Cre and (E) a DAT::Cre mouse. White arrows indicate selected eYFP+/TH+ neurons. (F) The proportion of eYFP+ neurons in the DRN co-labeled with TH and 5-HT in TH::Cre and (G) DAT::Cre mice. There was no significant difference in the proportion of eYFP+ neurons co-labeled with TH in TH::Cre and DAT::Cre mice (Chi-square = 1.2931, p = 0.2555, n = 977/1258 neurons for TH::Cre, n = 639/801 neurons for DAT::Cre).

We next considered whether acute social isolation affected the naturally occurring activity within DRN DA neurons. To address this question, we utilized a genetically encodable fluorescent calcium indicator, GCaMP6m (Chen et al., 2013), combined with fiber photometry to enable real-time recording of fluctuations in neural activity (Cui et al., 2013, Gunaydin et al., 2014). We targeted expression of GCaMP6m to DRN DA neurons by injection of an adeno-associated viral vector (AAV_5_) carrying GCaMP6m (AAV_5_-CAG-FLEX-GCaMP6m) into the DRN of tyrosine hydroxylase (TH)::IRES-Cre mice, which facilitated GCaMP6m expression in a Cre-dependent manner. An optic fiber implanted over the DRN allowed simultaneous delivery of 473 nm excitation light and collection of GCaMP6m emission by means of a dichroic and a photodetector (Figures 2A, 2B, and S2A).

To assess the effect of a social target on DRN DA activity, mice were recorded in their home cage during the introduction of a novel juvenile mouse. We compared the fluorescence signal, in response to initial contact with the juvenile mouse, in mice that had either been previously group-housed or socially isolated for 24 hr. Strikingly, in socially isolated mice, we observed a significant increase in the fluorescence signal in response to first contact with the juvenile mouse, compared with group-housed mice (Figures 2C–2F; Movie S1). Furthermore, in isolated mice, the activity in response to initial social contact was significantly greater than in response to initial interaction with a novel object (Figures 2C–2F).

This suggests that, following social isolation, the presence of a social stimulus is associated with a significant increase in DRN DA activity in vivo. This is consistent with our finding that synaptic inputs onto DRN DA neurons are potentiated following social isolation.

### DRN DA Neurons Release DA and Glutamate

We next sought to characterize the neurotransmitter content of DRN DA neurons, establish their sites of release, and validate parameters for subsequent causal experimentation. The TH+ DRN neurons have been confirmed as DAergic as they express aromatic L-amino decarboxylase (AADC), the enzyme that catalyzes conversion of L-3,4-dihydroxyphenylalanine, the product of TH, to DA (Lu et al., 2006) and the DA transporter (DAT) (Dougalis et al., 2012). Furthermore, DRN TH+ neurons lack dopamine-β-hydroxylase, which is necessary to convert DA to norepinephrine (Nagatsu et al., 1979), and do not express 5-hydroxytryptamine (5-HT) (Stratford and Wirtshafter, 1990).

**Figure 3:**
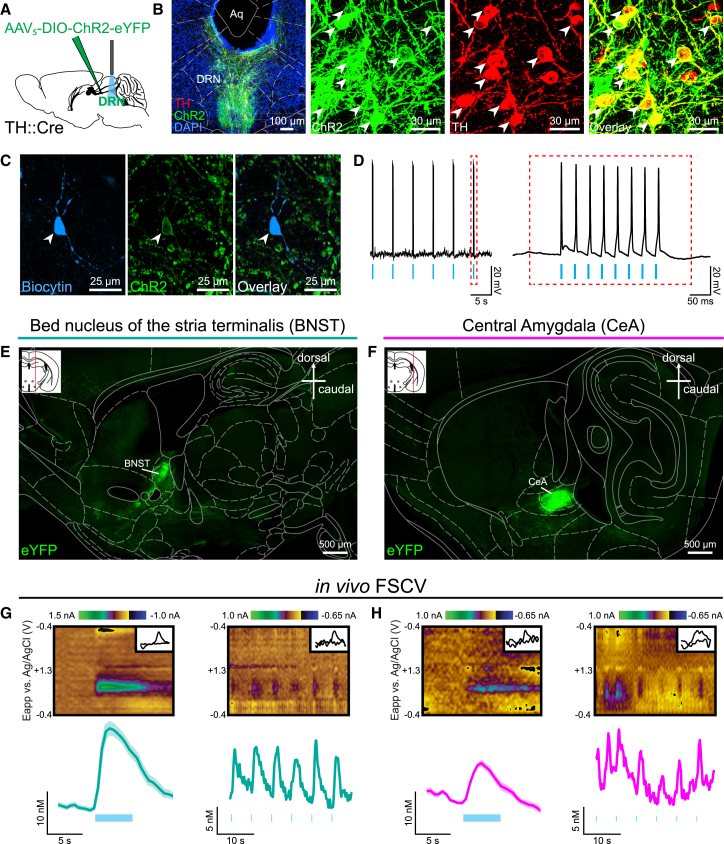
Photostimulation of DRN DA Neurons Elicits DA Release in the BNST and CeA (A) AAV_5_-DIO-ChR2-eYFP was injected into the DRN of TH::Cre mice to facilitate (B) ChR2 (green) expression in TH+ (red) DRN neurons. White arrows indicate selected co-labeled neurons. (C) Example of a biocytin-filled, ChR2-expressing, DRN neuron recorded using ex vivo electrophysiology. (D) ChR2 was activated using eight 5 ms pulses of blue light delivered every 5 s, which elicited a train of action potentials in the ChR2-expressing neuron. (E) Sagittal brain sections showing dense terminal expression in the BNST and (F) CeA following AAV_5_-DIO-eYFP injection into the DRN of a TH::Cre mouse. (G) In vivo FSCV was performed in anesthetized TH::Cre mice following Cre-dependent expression of ChR2 in the DRN. Example color plots and average traces (± SEM) from the BNST (n = 5 mice; 7 recording sites) and (H) CeA (n = 4 mice; 5 recording sites) showing DA release evoked by 150 5 ms pulses of blue light delivered at 30 Hz (left panels) and a representative trace showing eight 5 ms pulses delivered every 5 s (right panels). Eapp, applied potential. Insets show cyclic voltammograms from representative color plots. See also Figures S2 and S3.

Still, it remained to be demonstrated where these neurons synapse, which neurotransmitters they release, and whether their activation is sufficient to elicit detectable neurotransmitter release in vivo. To address these questions, we expressed Channelrhodopsin-2 fused to the enhanced yellow fluorescent protein (ChR2-eYFP) in the DRN in a Cre-dependent manner (Figures 3A and 3B). In TH::Cre mice we found that 77.7% of eYFP+ neurons co-labeled with TH using immunohistochemistry (Figures S2B, S2D, and S2F). This is consistent with a previous study (McDevitt et al., 2014) and is similar to overlap reported in other mouse lines used to selectively target DA neurons, including TH-GFP and Pitx3-GFP (Dougalis et al., 2012). For comparison, we also examined the DRN of DAT::IRES-Cre mice and found a similar proportion (79.8%) of eYFP+ neurons were colabeled with TH (Figure S2C, S2E, and S2G). As previously suggested (Hökfelt et al., 1976, Rogers, 1992), it is possible that some DA neurons within this region express low levels of TH, which may be below the detection threshold for immunohistochemistry and, thus, result in a relatively high proportion of seemingly eYFP+/TH− neurons. Importantly, eYFP expression did not overlap with 5-HT+ (serotonergic) neurons in either TH::Cre or DAT::Cre mice (Figures S2B–S2G).

In order to confirm optically induced firing in DRN DA neurons, we recorded from ChR2-expressing neurons using whole-cell patch-clamp electrophysiology in brain slices (Figure 3C). We delivered 473 nm light in a train of eight pulses of 5 ms pulse-width at 30 Hz every 5 s, a pattern used for VTA stimulation to elicit DA release and promote behavioral changes (Gunaydin et al., 2014, Tsai et al., 2009). In the DRN, ChR2-expressing neurons reliably followed these photostimulation parameters (Figure 3D).

Consistent with previous reports, eYFP expression in DRN DA somata resulted in terminal expression in several regions including the medial prefrontal cortex (mPFC), bed nucleus of the stria terminalis (BNST), lateral hypothalamus, central amygdala (CeA), entorhinal cortex, and basolateral amygdala (Hasue and Shammah-Lagnado, 2002, Meloni et al., 2006, Swanson, 1982, Yoshida et al., 1989). We observed particularly dense terminal expression within the dorsolateral BNST (dlBNST) (Figure 3E) and the lateral part of the CeA (Figure 3F) and, therefore, tested the effects of optical activation of DRN DA neurons on these regions.

**Figure S3:**
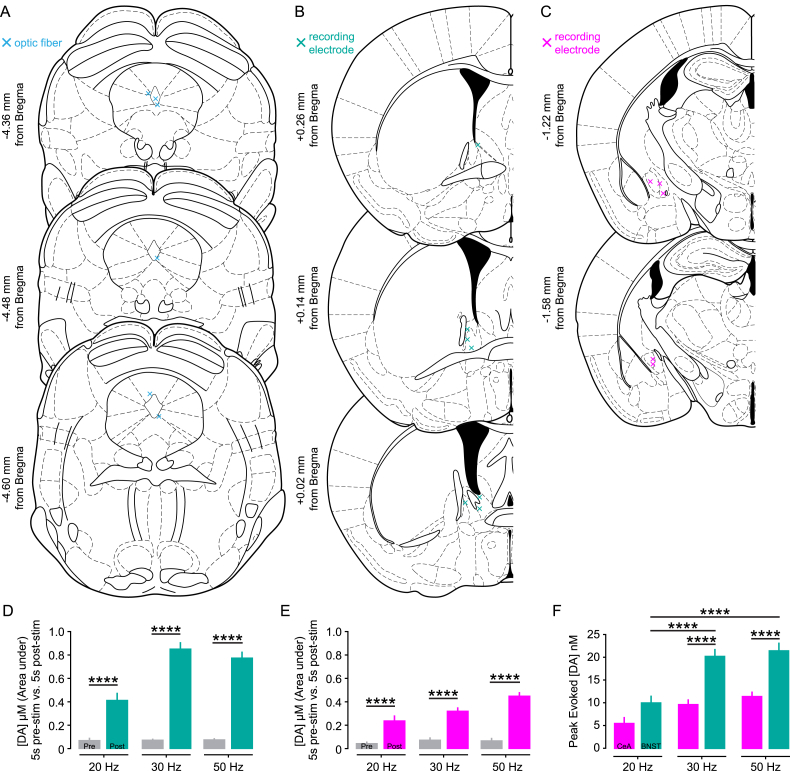
Placement of Optic Fiber Tip and Recording Sites for In Vivo FSCV and Evoked DA Release at Different Stimulation Frequencies, Related to Figure 3 (A) The position of the optic fiber tip (blue cross) in the DRN and (B) the recording electrode in the BNST (teal cross) and (C) CeA (magenta cross) of TH::Cre mice used for in vivo FSCV recordings. (D–E) Extracellular DA concentration ([DA]), evoked by 150 pulses of blue light (5 ms duration) at 20, 30, and 50 Hz, was quantified by calculating the area under each trace during the time window 5 s prior to stimulation onset compared to the time window 5 s post-stimulation onset. (D) Optical stimulation of DRN DA neurons evoked a significant increase in [DA] in the BNST compared to pre-stimulation levels at 20 Hz (paired t test: t_44_ = 5.761, ^∗∗∗∗^p < 0.0001), 30 Hz (paired t test: t_106_ = 13.74, ^∗∗∗∗^p < 0.0001), and 50 Hz (paired t test; t_105_ = 13.57, ^∗∗∗∗^p < 0.0001). (E) Optical stimulation of DRN DA neurons evoked a significant increase in [DA] in the CeA compared to pre-stimulation levels at 20 Hz (paired t test: t_51_ = 4.914, ^∗∗∗∗^p < 0.0001), 30 Hz (paired t test: t_70_ = 9.911, ^∗∗∗∗^p < 0.0001), and 50 Hz (paired t test: t_65_ = 10.64, ^∗∗∗∗^p < 0.0001). (F) As a group, peak [DA] was increased in a frequency-dependent manner (two-way ANOVA: F_2,441_ = 22.19, ^∗∗∗∗^p < 0.0001). In the BNST, peak [DA] evoked by 30 Hz and 50 Hz stimulation was greater than that evoked by 20 Hz (Bonferroni multiple comparisons: 20 Hz versus 30 Hz, ^∗∗∗∗^p < 0.0001; 20 Hz versus 50 Hz, ^∗∗∗∗^p < 0.0001; 30 Hz versus 50 Hz, p > 0.9999). In contrast, peak [DA] in the CeA did not differ significantly for different stimulation frequencies (Bonferroni multiple comparisons: 20 Hz versus 30 Hz, p = 0.7134; 20 Hz versus 50 Hz, p = 0.0806; 30 Hz versus 50 Hz, p > 0.9999). Peak-evoked [DA] was greater in the BNST compared to the CeA at 30 Hz (F_1,441_ = 54.39, ^∗∗∗∗^p < 0.0001; Bonferroni multiple comparisons: ^∗∗∗∗^p < 0.0001) and 50 Hz (Bonferroni multiple comparisons: ^∗∗∗∗^p < 0.0001). Data are represented as mean + SEM.

To confirm DA release in anesthetized TH::Cre mice, with Cre-dependent expression of ChR2 in the DRN, we performed in vivo fast-scan cyclic voltammetry (FSCV) (Figures S3A–S3C). Optical stimulation of DRN DA neurons elicited DA release in both the dlBNST (Figure 3G) and the CeA (Figure 3H). The peak-evoked DA release was greater in the dlBNST than the CeA at 30 Hz and 50 Hz (Figures S3D–S3F), suggesting possible differences in the dynamics of DA release and reuptake in these two regions. In response to eight pulses of 30 Hz stimulation, delivered every 5 s, DA transients were consistently recorded in the dlBNST. However, in the CeA, transients were inconsistent and signals did not adequately resolve as DA, perhaps suggesting DA release just below the FSCV detection threshold (Figures 3G and 3H).

**Figure 4:**
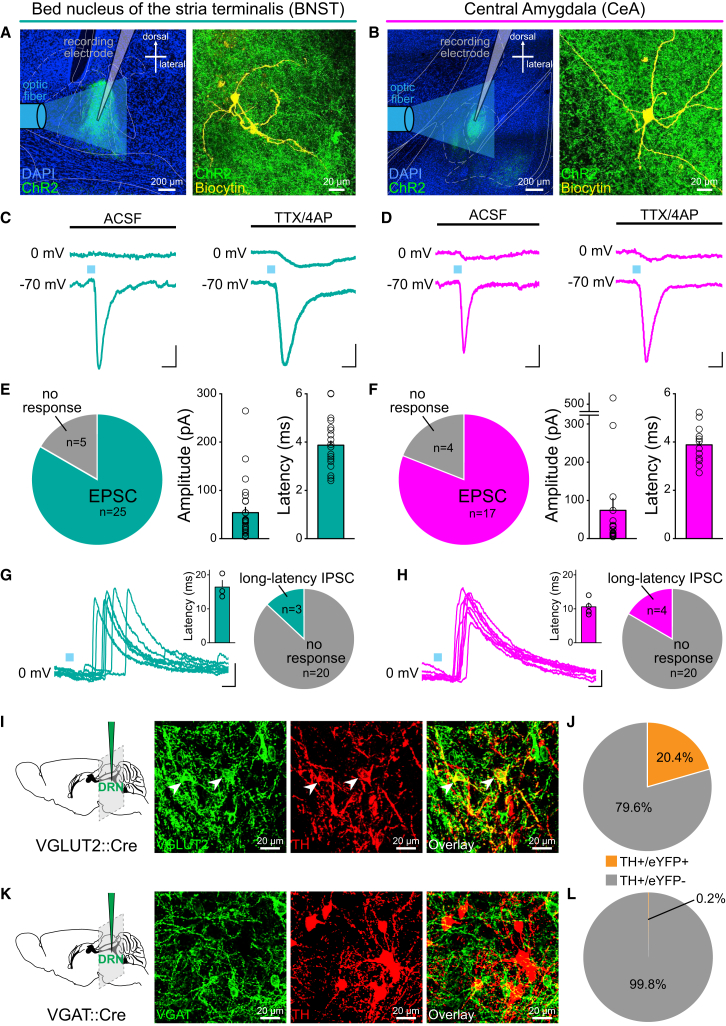
Optical Stimulation of DRN DA Neurons Elicits Monosynaptic Glutamate Release in the BNST and CeA (A) Ex vivo electrophysiology was performed in the BNST and (B) CeA following Cre-dependent expression of ChR2 in the DRN of TH::Cre and DAT::Cre mice. (C and D) Optical stimulation of ChR2-expressing terminals with a 5 ms blue light pulse elicited a short-latency, fast EPSC (measured in voltage-clamp at −70 mV), which persisted in the presence of TTX/4AP, but no detectable short-latency IPSC (measured at 0 mV). Scale bars, 10 pA, 10 ms. (E) The proportion of recorded neurons in the BNST and (F) CeA that responded with an EPSC (in the absence of TTX/4AP) and the amplitude and latency of each. (G and H) Individual traces showing the long-latency IPSCs elicited by successive 5 ms pulses of blue light, delivered every 20 s. Insets show average IPSC latency; pie charts show the proportion of cells which responded with an IPSC. Scale bars, 50 pA, 10 ms. (I and J) Confocal images showing eYFP-expressing neurons (green) in the DRN with post hoc immunohistochemistry for TH (red), following Cre-dependent expression of eYFP, in VGLUT2::Cre and (K and L) VGAT::Cre mice. White arrows indicate selected co-labeled cells. A significantly greater proportion of TH+ neurons co-expressed VGLUT2 (n = 119/582 neurons) compared with VGAT (n = 1/577 neurons; Chi-square = 128.30, p < 0.0001). Data are represented as mean ± SEM. See also Figure S4.

**Figure S4:**
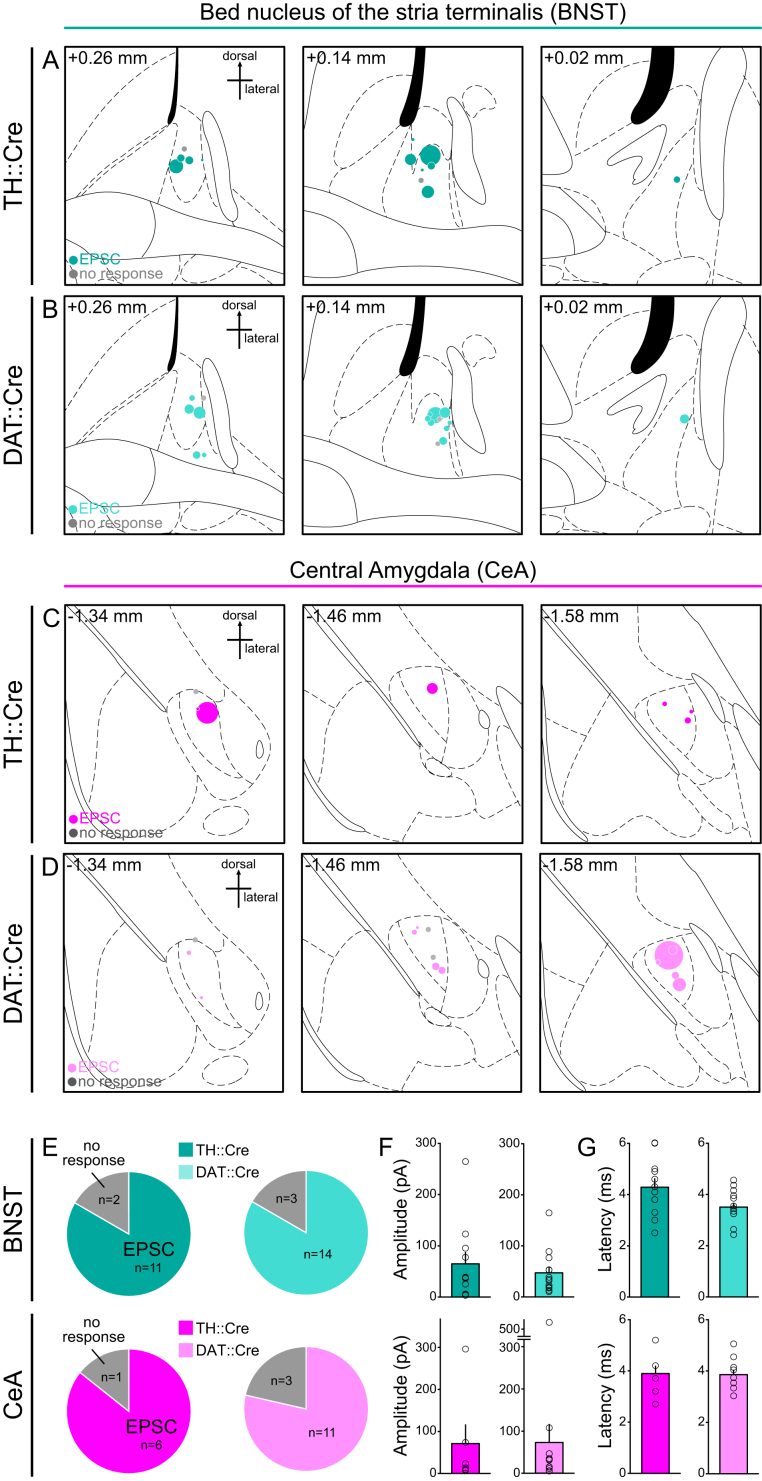
Optically Evoked Glutamate Currents in the BNST and CeA Following DRN DA Terminal Stimulation in TH::Cre and DAT::Cre Mice, Related to Figure 4 (A and B) Location of ex vivo recorded cells in the BNST and (C and D) CeA of TH::Cre and DAT::Cre mice, superimposed on coronal brain atlas sections (distance from Bregma shown in top left corner). The relative amplitude of the optically evoked EPSC from each cell is indicated by the size of the teal (BNST) or magenta (CeA) circle. Cells with no response are displayed in gray. (E) Pie charts showing the proportion of cells that responded with an EPSC to a 5 ms blue light pulse in the BNST and CeA of TH::Cre and DAT::Cre mice and (F) the amplitude and (G) latency of each EPSC. There was no significant difference in the proportion of neurons which responded to optical stimulation with an EPSC in TH::Cre mice compared to DAT::Cre mice in the BNST (Chi-square = 0.0271, p = 0.8691) or CeA (Chi-square = 0.1544, p = 0.6944). Data are represented as mean + SEM

To determine whether DRN DA neurons co-release glutamate and/or GABA in downstream targets, we prepared brain slices containing the dlBNST (Figure 4A) or CeA (Figure 4B) from TH::Cre and DAT::Cre mice expressing ChR2 in a Cre-dependent manner in the DRN and recorded from neurons within the region of terminal expression (Figures S4A–S4D). Photostimulation of ChR2-expressing DA terminals elicited a short-latency fast AMPAR-mediated excitatory postsynaptic current (EPSC) in 25/30 neurons recorded in the dlBNST and 17/21 neurons in the CeA (Figure 4C-F). There was no significant difference in the proportion of neurons responding with an EPSC in TH::Cre and DAT::Cre mice, so these data were pooled (Figures S4E–S4G). Furthermore, the EPSCs persisted in the presence of tetradotoxin (TTX) and 4-aminopyridine (4AP), suggesting that they represent monosynaptic glutamate release from DRN DA terminals (Petreanu et al., 2007).

In contrast, optical stimulation of DAergic terminals did not elicit a short-latency GABA_A_-mediated inhibitory postsynaptic current (IPSC) in the dlBNST or CeA (Figures 4C and 4D). However, in 3/23 dlBNST neurons and 4/24 CeA neurons we observed IPSC responses with a long and variable latency (Figures 4G and 4H), suggesting that terminal stimulation can activate GABAergic neurons to elicit a polysynaptic IPSC.

We next confirmed the presence of a glutamatergic, but not GABAergic, marker in DRN DA neurons using two lines of transgenic mice: vesicular glutamate transporter 2 (VGLUT2)::IRES-Cre and vesicular GABA transporter (VGAT)::IRES-Cre. With Cre-dependent expression of eYFP in the DRN, a subset of TH+ neurons co-expressed VGLUT2 (Figures 4I and 4J), yet there was almost no overlap with VGAT (Figures 4K and 4L). This is consistent with the observed co-expression of VGLUT2 in more caudal and medially located DA neurons (Kawano et al., 2006).

Taken together, this demonstrates that optical stimulation of ChR2-expressing DRN DA neurons is sufficient to trigger rapid DA and glutamate release in two major downstream targets.

### Optogenetic Activation of DRN DA Neurons Mimics a Loneliness-like State

**Figure 5:**
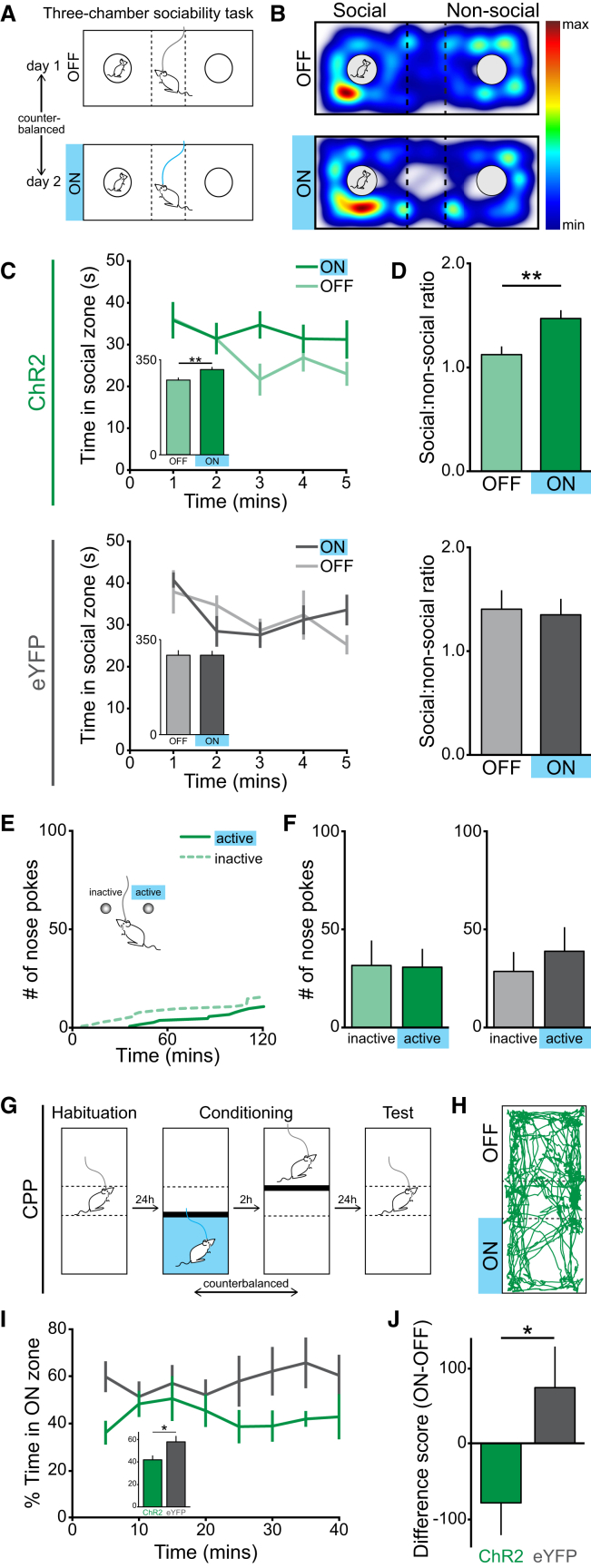
Optical Activation of DRN DA Neurons Elicits Social Preference and Place Avoidance (A) TH::Cre mice were tested for social preference in the three-chamber sociability task. (B) Representative spatial heat maps showing the location of a ChR2-expressing mouse. (C) Time spent by ChR2- and eYFP-expressing mice in the social zone across the first 5 min of the task, with and without photostimulation (inset shows total time). ChR2-expressing, but not eYFP-expressing, mice showed a significant increase in the total time spent in the social zone, relative to the non-social zone, when receiving blue light stimulation (ChR2, inset, paired t test: t_10_ = 3.297, ^∗∗^p = 0.0081, n = 11; eYFP: t_10_ = 0.0100, p = 0.9922; n = 11) and (D) a significant increase in the social:non-social ratio (ChR2, paired t test: t_10_ = 3.843, ^∗∗^p = 0.0032, n = 11; eYFP: t_10_ = 0.1847, p = 0.8572, n = 11). (E) Cumulative activity graph of nose pokes made by a ChR2-expressing mouse at the inactive and active (light-paired) ports during an ICSS task. (F) Optical stimulation of DRN DA neurons did not support ICSS, as revealed by the number of nose pokes made into the inactive and active ports by ChR2- (active versus inactive, paired t test: t_4_ = 0.0811, p = 0.9393, n = 5) and eYFP-expressing mice (active versus inactive, paired t test: t_6_ = 0.732, p = 0.4917, n = 7). (G) CPP paradigm and (H) representative track from a ChR2-expressing mouse during the first 10 min of the test session. (I) Graph showing % time spent in the previously light-paired zone (inset shows first 10 min). ChR2-expressing mice showed significant avoidance of the previously light-paired zone, relative to eYFP mice, as shown by the % time spent in this zone (unpaired t test: t_10_ = 2.393, ^∗^p = 0.0378, n = 6 ChR2, 6 eYFP) and (J) the difference between the time spent in the previously light-paired and unpaired zones (unpaired t test: t_10_ = 2.241, ^∗^p = 0.0489, n = 6 ChR2, 6 eYFP). Data are represented as mean ± SEM. See also Figure S5.

**Figure S5:**
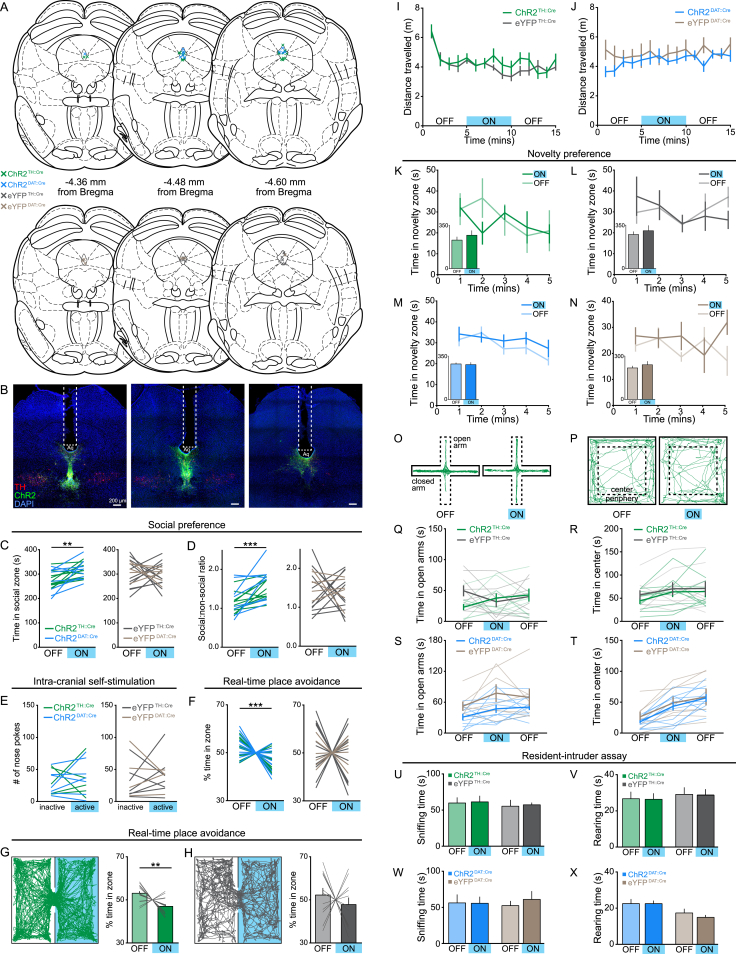
Effect of Photoactivation in TH::Cre and DAT::Cre Mice on Behavioral Measures of Social Preference, Avoidance, Arousal, and Anxiety-Related Behavior, Related to Figures 5 and 7 (A) The position of the optic fiber tip above the DRN of TH::Cre and DAT::Cre mice expressing ChR2 or eYFP used for behavioral analysis. (B) Example confocal images showing the optic fiber track (white dashed line), ChR2-eYFP expression (green), and post hoc immunohistochemistry for TH (red) in the DRN of TH::Cre mice at each anteroposterior position indicated in A. (C) Time spent in the social zone of the three-chamber apparatus and (D) social:non-social ratio of individual TH::Cre and DAT::Cre mice expressing ChR2 (green and blue, respectively) or eYFP (gray and brown, respectively) in DRN DA neurons. There was no significant difference in the behavior of TH::Cre and DAT::Cre mice with blue light stimulation (ChR2: time in social zone – unpaired t test: t_17_ = 0.0565, p = 0.9556; social:non-social ratio – t_17_ = 1.185, p = 0.2523, n = 11 TH::Cre, 8 DAT::Cre; eYFP: time in social zone – unpaired t test: t_15_ = 0.0165, p = 0.9870; social:non-social ratio – t_15_ = 0.3015, p = 0.7672, n = 11 TH::Cre, 6 DAT::Cre) or without stimulation (ChR2: time in social zone – unpaired t test: t_17_ = 0.1162, p = 0.9089; social:non-social ratio – t_17_ = 0.7161, p = 0.4836, n = 11 TH::Cre, 8 DAT::Cre; eYFP: time in social zone – unpaired t test: t_15_ = 0.8873, p = 0.3889; social:non-social ratio – t_15_ = 0.8239, p = 0.4229, n = 11 TH::Cre, 6 DAT::Cre). Pooling data from TH::Cre and DAT::Cre mice revealed that blue light stimulation caused a significant increase in the time spent in the social zone and social:non-social ratio of ChR2- (time in social zone – paired t test: t_18_ = 3.877, ^∗∗^p = 0.0011; social:non-social ratio – t_18_ = 4.527, ^∗∗∗^p = 0.0003, n = 19), but not eYFP-expressing mice (time in social zone – paired t test: t_16_ = 0.4094, p = 0.6876; social:non-social ratio – t_16_ = 0.5364, p = 0.5991, n = 17). (E) In an ICSS task, there was no significant difference in the number of nose pokes made into the active or inactive ports by TH::Cre and DAT::Cre mice expressing ChR2 (active – unpaired t test: t_10_ = 0.2347, p = 0.8192; inactive – unpaired t test: t_10_ = 0.3284, p = 0.7494, n = 5 TH::Cre, 7 DAT::Cre) or eYFP (active – unpaired t test: t_19_ = 0.2205, p = 0.8304; inactive – unpaired t test: t_9_ = 0.7528, p = 0.4708, n = 7 TH::Cre, 4 DAT::Cre). (F) The proportion of time spent in the stimulation zone during an RTPA assay was not significantly difference between TH::Cre and DAT::Cre mice expressing ChR2- (unpaired t test: t_26_ = 0.1678, p = 0.8681, n = 17 TH::Cre, 11 DAT::Cre) or eYFP (unpaired t test: t_15_ = 0.5274, p = 0.6056, n = 11 TH::Cre, 6 DAT::Cre). Pooling data from TH::Cre and DAT::Cre mice revealed that ChR2- but not eYFP-expressing mice spent a significantly greater proportion of time in the unstimulated zone (ChR2 – paired t test: t_27_ = 4.033, ^∗∗∗^p = 0.0004, n = 28; eYFP: paired t test: t_16_ = 0.5849, p = 0.5668, n = 17). (G) Representative track from a ChR2-expressing and (H) eYFP-expressing TH::Cre mouse in the RTPA assay. ChR2- (paired t test: t_16_ = 3.248, ^∗∗^p = 0.005, n = 17), but not eYFP-expressing TH::Cre mice (paired t test: t_10_ = 0.6779, p = 0.5132, n = 11) spent a significantly lower proportion of time in the light-paired zone of the chamber. (I and J) There was no effect of blue light stimulation on locomotion in the open field as measured by total distance traveled in the arena (TH::Cre – two-way ANOVA: group × light interaction: F_2,34_ = 0.6333, p = 0.5370, n = 10 ChR2, 9 eYFP; DAT::Cre – two-way ANOVA: group × light interaction: F_2,30_ = 0.2483, p = 0.7817, n = 10 ChR2, 7 eYFP). (K-N) Novelty preference was assessed in a modified three-chamber task in which a novel object was placed under one inverted cup instead of a juvenile mouse. Optical stimulation of DRN DA neurons did not significantly affect time spent exploring the novelty zone in (K and L) ChR2- (TH::Cre – paired t test: t_4_ = 0.9499, p = 0.3959, n = 5; DAT::Cre – paired t test: t_8_ = 0.1215, p = 0.9063, n = 9) or (M and N) eYFP-expressing mice (TH::Cre – paired t test: t_5_ = 0.8107, p = 0.4544, n = 6; DAT::Cre – paired t test: t_5_ = 0.6382, p = 0.5514, n = 6). (O) Mice were assessed for anxiety-related behavior in the elevated plus maze (EPM) and (P) open field test across a 15 min trial with the middle 5 min paired with blue light stimulation. Representative tracks from a ChR2- (green) and an eYFP-expressing (gray) TH::Cre mouse showing the first ‘light OFF’ and second ‘light ON’ epochs. (Q–T) There was no significant effect of light stimulation on (Q and S) the time spent in the open arms of the EPM in ChR2- relative to eYFP-expressing mice (TH::Cre - two-way ANOVA: group × light interaction: F_2,34_ = 3.016, p = 0.0623, n = 10 ChR2, 9 eYFP; DAT::Cre – two-way ANOVA: group × light interaction: F_2,32_ = 0.3468, p = 0.7095, n = 11 ChR2, 7 eYFP) or (R and T) the time spent in the center of the open field (TH::Cre – two-way ANOVA: group × light interaction: F_2,34_ = 0.1201, p = 0.8872, n = 10 ChR2, 9 eYFP; DAT::Cre – two-way ANOVA: group × light interaction: F_2,30_ = 0.2322, p = 0.7942, n = 10 ChR2, 7 eYFP). (U and V) In a non-exploratory assay for anxiety-related behavior, the resident-intruder task, blue light stimulation had no effect on total time spent sniffing the juvenile intruder mouse (TH::Cre – ChR2: paired t test: t_11_ = 0.1924, p = 0.8510, n = 12; eYFP: t_11_ = 0.2378, p = 0.8164; n = 12; DAT::Cre – ChR2: paired t test: t_6_ = 0.01584, p = 0.9879, n = 7; eYFP: t_4_ = 1.158, p = 0.3113; n = 5) or (W and X) rearing (TH::Cre – ChR2: t_11_ = 0.1012, p = 0.9213; n = 12; eYFP: t_11_ = 0.1133, p = 0.9118, n = 12; DAT::Cre – ChR2: paired t test: t_6_ = 0.02656, p = 0.9797, n = 7; eYFP: t_4_ = 0.9856, p = 0.3801; n = 5). Data are represented as mean ± SEM.

To test for a causal relationship between DRN DA activation and an increase in sociability, we combined ChR2-mediated photostimulation with freely moving behavior (Figures S5A and S5B). We assessed social preference utilizing the three-chamber sociability task, in which time spent on the “social” side of the chamber (containing a juvenile mouse under a wire cup) is used as a measure of sociability (Moy et al., 2004, Silverman et al., 2010). Here, social approach is solely controlled by the experimental animal, as containment of the juvenile mouse removes the potential threat of territorial disputes, permitting an unadulterated measure of social interest (Silverman et al., 2010). We found that in TH::Cre mice expressing ChR2, but not eYFP, optical stimulation resulted in a significant increase in the proportion of time spent in the social zone (Figures 5A–5D), a result we replicated in DAT::Cre mice (Figures S5C and S5D).

This suggests that activation of DRN DA neurons plays a causal role in driving social behavior. Similarly, however, it has been shown that calcium signals can be detected in VTA DA neurons in response to a social target, and optical stimulation of these neurons promotes social interaction (Gunaydin et al., 2014), which has led to the hypothesis that VTA DA neurons represent a neural substrate for social reward. If DRN DA neurons were also encoding social reward, we would expect increased activity within these neurons to be positively reinforcing, similar to VTA activation, which has been shown to support ICSS (Witten et al., 2011) and conditioned place preference (CPP) (Tsai et al., 2009). Conversely, if DRN DA neurons are motivating social approach, due to an unmet need for social contact resulting from isolation, we would expect increased activity in these neurons (in the absence of a social stimulus) to elicit a negative affective state.

To distinguish between these two possibilities, we tested mice on an ICSS paradigm. However, we found that optical stimulation of DRN DA neurons did not support ICSS in TH::Cre (Figures 5E and 5F) or DAT::Cre mice (Figure S5E). Next, we assessed behavior in a real-time place avoidance (RTPA) assay, whereby mice freely explored a chamber in which one half was paired with blue light stimulation. Here, we observed avoidance of the light-paired side of the chamber relative to the unstimulated side in ChR2-, but not eYFP-expressing, TH::Cre and DAT::Cre mice (Figures S5F–S5H). Additionally, to eliminate confounding effects of stimulation-induced arousal, we examined behavior in a CPP paradigm (Figure 5G). During the test session, ChR2-expressing mice showed significant avoidance of the stimulation-associated zone, relative to eYFP-expressing mice (Figures 5H–5J). Furthermore, using additional behavioral assays, we found that optical stimulation of DRN DA neurons had no detectable effect on locomotion, novelty preference, or anxiety-related behavior (Figures S5I–S5X). This suggests that, in stark contrast to VTA DA neurons, optical activation of DRN DA neurons produces an aversive state.

In sum, we find that increasing activity of DRN DA neurons in group-housed mice promotes social preference but also elicits a negative affective state in the absence of a social target. We posit that this recapitulates a loneliness-like state, in which social approach is driven to alleviate the aversive state associated with social isolation.

### Photoinhibition of DRN DA Neurons Reduces Isolation-Induced Sociability

**Figure S6:**
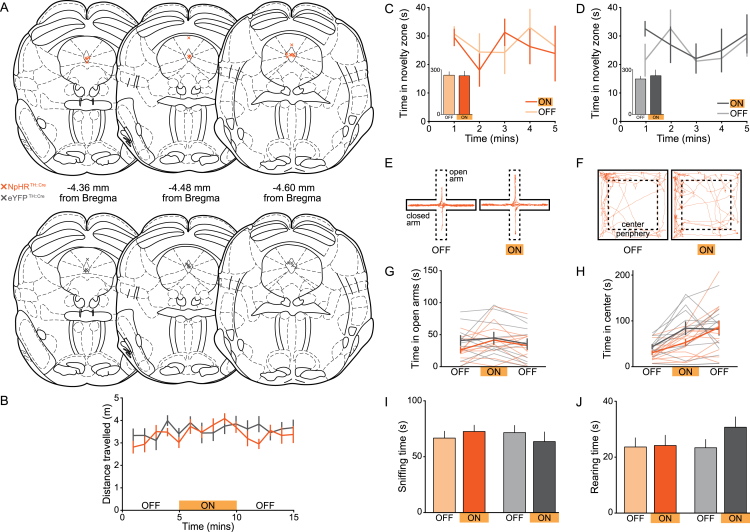
Effect of Photoinhibition in TH::Cre Mice on Behavioral Measures of Arousal and Anxiety-Related Behavior, Related to Figure 6 (A) The position of the optic fiber tip above the DRN of TH::Cre mice expressing NpHR (orange crosses) or eYFP (gray crosses) used for behavioral analysis. (B) No significant difference was detected between NpHR- and eYFP-expressing mice in distance traveled in the open field. (C) In an assay for novelty preference, optical inhibition of DRN DA neurons had no effect on the time spent in the novelty zone in NpHR- (paired t test: t_5_ = 0.08958, p = 0.9321, n = 6) or (D) eYFP-expressing mice (paired t test: t_6_ = 0.4595, p = 0.6620; n = 7). (E) Representative track from a TH::Cre mouse expressing NpHR in DRN DA neurons in the EPM and (F) open field before light stimulation and during delivery of constant yellow light. (G) There was no significant effect of photoinhibition on time spent in the open arms of the EPM (two-way ANOVA: group × light interaction: F_2,38_ = 1.009, p = 0.3742, n = 11 NpHR, 10 eYFP) or (H) time spent in the center of the open field (two-way ANOVA: group × light interaction: F_2,46_ = 2.138, p = 0.1294, n = 13 NpHR, 12 eYFP) in NpHR- relative to eYFP-expressing mice. (I) In the resident-intruder task optical inhibition did not significantly affect time spent sniffing the juvenile (NpHR: paired t test – t_13_ = 0.8025, p = 0.4367, n = 14; eYFP: t_9_ = 1.628, p = 0.1380; n = 10) or (J) time spent rearing (NpHR: t_13_ = 0.0619, p = 0.9516, n = 14; eYFP: t_9_ = 1.679, p = 0.1274; n = 10). Data are represented as mean ± SEM.

Typically, in response to situations of social isolation or loneliness, individuals are motivated to re-establish social contact (Maner et al., 2007) and pay greater attention to social stimuli (Gardner et al., 2005, Pickett et al., 2004). In rodents, even an acute period of social isolation can elicit a rebound increase in social behavior (Niesink and van Ree, 1982, Panksepp and Beatty, 1980). In order to test whether DRN DA neurons are required to promote sociability following social isolation, we expressed a hyperpolarizing opsin (NpHR) in a Cre-dependent manner in the DRN of TH::Cre mice to mediate optical inhibition (Figure S6A).

**Figure 6:**
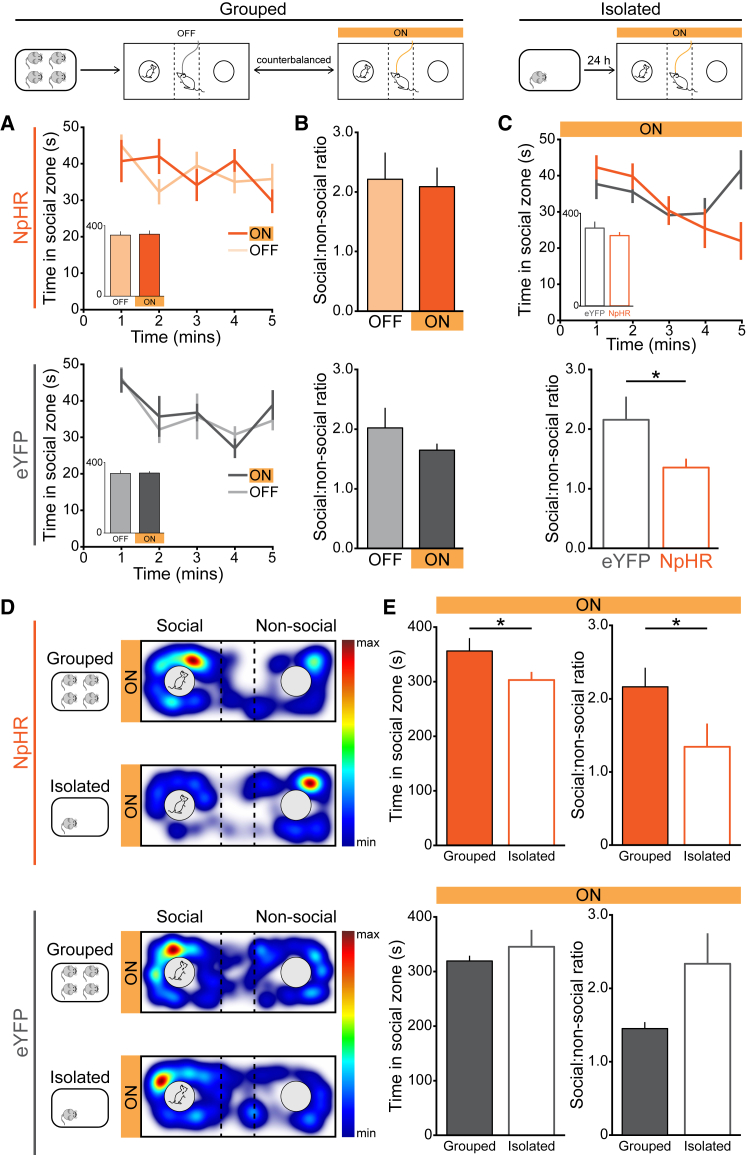
Optical Inhibition of DRN DA Neurons Reduces Social Preference Only Following Isolation (A) Group-housed TH::Cre mice expressing NpHR (upper panels) or eYFP (lower panels) in the DRN, in a Cre-dependent manner, were assessed in the three-chamber sociability task. Time spent in the social zone across the first 5 min of the task (inset shows total time) and (B) the social:non-social ratio. Optical inhibition did not produce a detectable difference in the total time spent in the social zone (NpHR, inset, paired t test: t_12_ = 0.1778 p = 0.8619, n = 13; eYFP: t_9_ = 0.1788, p = 0.8621, n = 10) or the social:non-social ratio (NpHR, paired t test: t_12_ = 0.2414, p = 0.8133, n = 13; eYFP: t_9_ = 1.293, p = 0.2282, n = 10). (C) Mice were isolated from their cagemates for 24 hr and then tested for sociability with optical inhibition. Time spent in the social zone by NpHR- and eYFP-expressing mice across the first 5 min (inset shows total time) and social:non-social ratio following isolation. NpHR-expressing mice spent a significantly lower proportion of time in the social zone after 24 hr of isolation compared with eYFP-expressing mice (social:non-social ratio, unpaired t test: t_16_ = 2.236, ^∗^p = 0.0400, n = 7 eYFP, 11 NpHR). (D) Representative spatial heat maps showing the location of an NpHR- (upper panels) and eYFP-expressing mouse (lower panels) in the first 5 min of the task, when group-housed and following 24 hr of isolation. (E) Time spent in the social zone and social:non-social ratio of mice tested with photoinhibition while group-housed and following social isolation. Photoinhibition in NpHR-expressing mice resulted in a significant reduction in time spent in the social zone (paired t test: t_10_ = 2.740, ^∗^p = 0.0208, n = 11) and social:non-social ratio (paired t test: t_10_ = 2.239, p = ^∗^0.0491, n = 11) following social isolation, compared to when group-housed. Data are represented as mean ± SEM. See also Figure S6.

Our in vivo recordings (Figure 2) revealed that the increase in DRN DA activity, on initial contact with a social target, was significantly greater in isolated, compared with group-housed, mice. First, therefore, we tested whether optical inhibition of DRN DA neurons altered social preference in group-housed mice using the three-chamber sociability task. Consistent with our recording results, neither NpHR- nor eYFP-expressing mice showed a significant difference in the proportion of time spent in the social zone with optical inhibition (Figure 6A and 6B). Furthermore, we did not observe significant effects of optical inhibition on behavioral measures of arousal or anxiety-related behavior (Figures S6B–S6J).

We next socially isolated NpHR- and eYFP-expressing mice for 24 hr and then tested social preference with optical inhibition. This revealed that mice expressing NpHR spent a significantly lower proportion of time in the social zone, compared with eYFP-expressing mice (Figure 6C). We then compared the social preference of mice tested with optical inhibition while group-housed and following social isolation. While eYFP-expressing mice showed the typical trend toward an increase in social preference following isolation, optical inhibition in NpHR-expressing mice resulted in a significant decrease in social preference following isolation (Figures 6D and 6E).

Collectively, these data suggest that inhibition of DRN DA neurons prevents the typical restoration of social contact following a period of isolation. This supports the hypothesis that DRN DA activity is required for motivating sociability in response to the negative state of isolation.

### Prior Social Experience Predicts Functional Role of DRN DA Neurons

**Figure 7:**
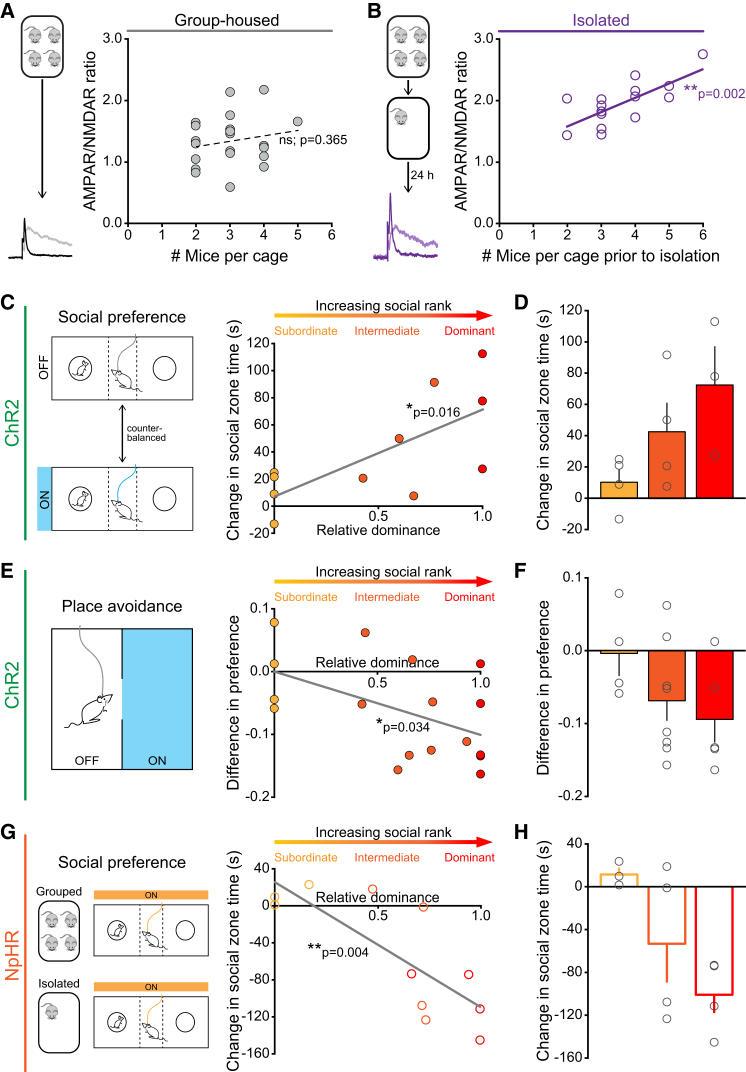
Prior Social Experience Predicts Magnitude of Social Isolation-Induced Synaptic Potentiation and Behavioral Response to Optogenetic Manipulation (A and B) Scatter plot of AMPAR/NMDAR ratios recorded in DRN DA neurons plotted against the number of mice per cage in group-housed mice, and (B) the number of mice previously housed per cage in socially isolated mice. No significant correlation was detected in group-housed mice (Pearson’s correlation: p = 0.3653, r^2^ = 0.0374, n = 24), but a significant positive correlation was found in socially isolated mice (Pearson’s correlation: ^∗∗^p = 0.0021, r^2^ = 0.5282; n = 15). (C) Relative social rank of TH::Cre mice was estimated with a score of 0 indicating the most subordinate and 1 the most dominant mouse in each cage. Relative dominance of ChR2-expressing mice plotted against the change in time spent in the social zone of the three-chamber apparatus (with blue light stimulation − without stimulation). There was a significant positive correlation between relative dominance and change in social zone time (Pearson’s correlation: ^∗^p = 0.0163, r^2^ = 0.4913; n = 11). (D) Mean (+ SEM) change in social-zone time for mice of each social rank. (E) Relative dominance of ChR2-expressing mice plotted against the difference in preference for the light-stimulation zone (proportion of time spent in the stimulation zone − proportion of time spent in the unstimulated zone) in the RTPA assay. There was a significant negative correlation between relative dominance and preference for the stimulation zone (Pearson’s correlation: ^∗^p = 0.0338, r^2^ = 0.2668, n = 17). (F) Mean (− SEM) difference in preference for stimulation zone for mice of each social rank. (G) Relative dominance of NpHR-expressing mice plotted against the change in social-zone time (isolated with yellow light – grouped with yellow light). There was a significant negative correlation between relative dominance and the change in social-zone time following isolation (Pearson’s correlation: ^∗∗^p = 0.0038, r^2^ = 0.6241, n = 11). (H) Mean (± SEM) change in social zone time for mice of each social rank. See also Figure S7.

Social groups vary in terms of their size, complexity, and the nature of interactions between individuals. Given that the size of social networks has been correlated to structural differences in the brain (Sallet et al., 2011), we considered whether the degree of synaptic potentiation induced by social isolation would be related to the previous social group size. We compared data from mice that either remained group-housed or experienced social isolation and found that the magnitude of the AMPAR/NMDAR ratio was positively correlated with the number of previous cagemates in socially isolated, but not continually group-housed, mice (Figures 7A and 7B). This suggests that prior social environment contributes to subsequent isolation-induced synaptic strength.

Mice, like primates, form dominance hierarchies when housed together, which are thought to aid stability of social groups (Drews, 1993, Uhrich, 1938). Dominant behavior in males includes agonistic displays of behavior, priority access to food and resources, territorial urine marking, and winning situations of social conflict (Wang et al., 2014). As a result, the subjective experience of social interaction is likely to differ between members of a social group dependent on their social rank. Thus, we next hypothesized that social rank could influence the behavioral effects elicited by activation or inhibition of DRN DA neurons.

**Figure S7:**
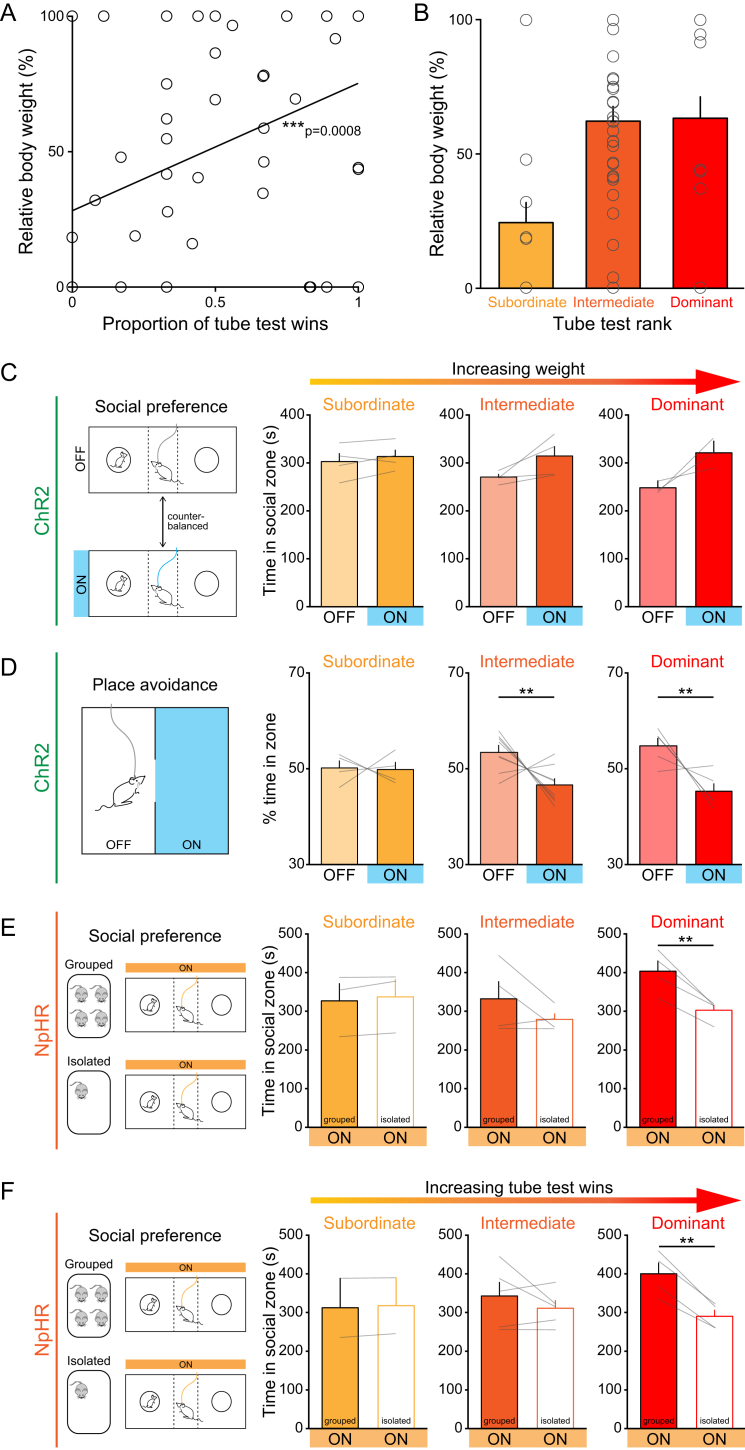
Social Rank Predicts the Behavioral Effect of Photoactivation and Photoinhibition of DRN DA Neurons, Related to Figure 7 (A) Proportion of ‘wins’ in the tube test for social dominance plotted against the relative body weight of TH::Cre mice. There was a significant positive correlation between the proportion of wins and body weight (Spearman’s rank correlation: ^∗∗∗^p = 0.0008, r^2^ = 0.1869; n = 57). (B) Relative body weight of all mice classified as subordinate, intermediate, and dominant based on performance in the tube test. (C) Time spent by ChR2-expressing mice in the social zone of the three-chamber apparatus, with and without blue light stimulation, ranked based on relative body weight. (D) % time spent in the light-paired and unstimulated zones in the RTPA assay, by ChR2-expressing mice ranked based on relative body weight. (E) Time spent by NpHR-expressing mice in the social zone of the three-chamber apparatus when receiving yellow light while group-housed and following social isolation, ranked based on relative weight or (F) proportion of wins in the tube test. Data are represented as mean +SEM. Paired t tests used for within-group comparisons, ^∗^p < 0.05, ^∗∗^p < 0.01.

We estimated relative dominance within each cage (see Supplemental Experimental Procedures and Figures S7A and S7B) and examined the relationship between social rank and the change in social preference elicited by photoactivation of DRN DA neurons (Figure 5C). Intriguingly, we found that relative dominance and change in social preference were positively correlated, such that optical stimulation of DRN DA neurons appeared to be more effective at promoting social preference in dominant mice (Figures 7C, 7D, and S7C). Next, we assessed the relationship between social dominance and preference for the optical stimulation zone in ChR2-expressing mice tested in the RTPA assay (Figure S5G). Here, we observed a negative correlation between relative dominance and preference for the stimulation zone, such that more dominant mice displayed greater avoidance of the light-paired side of the chamber (Figures 7E, 7F, and S7D).

Finally, we examined the change in social preference of NpHR-expressing mice when receiving optical inhibition while group-housed compared to following isolation. Conversely, we observed a significant negative correlation (Figures 7G, 7H, S7E, and S7F), indicating that photoinhibition produced a greater reduction in social preference following isolation in more dominant mice.

Thus, in all cases, dominant mice showed a greater degree of behavioral modulation upon optogenetic manipulation of DRN DA neurons.

## Discussion

### Satisfying the Profile of a Neural Substrate for a Loneliness-like State

The characteristics we have observed in DRN DA neurons bear remarkable similarities to the hypotheses generated from human psychology in describing the “need to belong” (Baumeister and Leary, 1995). First, it has been hypothesized that a “social monitoring system” exists which assimilates information on an individual’s current and desired level of social acceptance (Gardner et al., 2005, Leary et al., 1995). Our finding that acute social isolation induces synaptic plasticity at synapses onto DRN DA neurons (Figure 1) suggests that these neurons either play a role in detecting or in reconciling the disparity between the current and desired social environment.

Second, in a state of loneliness, wherein an individual’s basic need for social connection is unmet (Peplau, 1978), processing of socially relevant information should be prioritized (Baumeister and Leary, 1995). Indeed, in humans, socially excluded individuals display an enhanced memory for social events (Gardner et al., 2000), and more lonely individuals show increased attention toward social cues (Gardner et al., 2005, Pickett et al., 2004). Consistently, in socially isolated mice, we observed a significantly greater increase in DRN DA activity during initial contact with a social target (Figure 2), compared with group-housed mice.

Third, it has been postulated that the motivation for social re-connection should elicit “goal-orientated behavior” (Baumeister and Leary, 1995). With photoactivation, we revealed that activating DRN DA neurons promoted social preference in group-housed mice (Figure 5). However, in the absence of a social target, mice avoided photoactivation of DRN DA neurons, suggesting that stimulation is aversive. This suggests that activation of these neurons may be recapitulating a loneliness-like state, which is marked by a negative-affective state in which the drive to seek social contact is increased.

Fourth, the motivation for social re-connection was hypothesized to be “sensitive to satiation patterns” (Baumeister and Leary, 1995). In line with this, in group-housed mice, we observed limited changes in DRN activity related to initial juvenile contact (Figure 2), and photoinhibition of these neurons did not alter social behavior (Figure 6). Therefore, under “sated” group-housed conditions, this type of motivation may not be playing a major role. In contrast, following social isolation, photoinhibition caused a reduction in social preference (Figure 6). This indicates that the activity within these neurons may only be necessary in situations in which the motivation for social contact is high, such as that experienced after social isolation.

It has been hypothesized that the “need to belong” represents a powerful motivational drive, comparable to the basic need for food in a state of hunger (Baumeister and Leary, 1995). In considering this analogy with feeding behavior, it is intriguing to note that distinct neural circuits are thought to motivate food consumption related to the rewarding value of food (Nieh et al., 2015) and the need to obtain food to alleviate the negative state of hunger (Chen et al., 2015, Sternson et al., 2013). Thus, in a similar manner, social behavior may be driven by distinct neural circuits when motivated by social reward and when motivated by the punishment of social isolation.

### The Subjective Experience of Social Isolation

In humans, a clear distinction can be made between an individual’s subjective (perceived) isolation and their objective isolation. Perceived social isolation (or loneliness) reflects the quality of an individual’s social interactions (Hawkley et al., 2008, Peplau, 1978) rather than their quantity or frequency. In humans, perceived social isolation predicts a poor outcome in numerous physical and mental health-related measures, entirely independent of the level of objective isolation (Adam et al., 2006, Cacioppo et al., 2006, Hawkley et al., 2006, Wen et al., 2006). While “loneliness” per se is difficult to directly test in mice, and consequently, the lack of animal literature on this phenomenon is noted (Cacioppo et al., 2014), social rank offers a useful estimate of an individual’s subjective social experience.

When we overlaid this measure onto our behavioral data, remarkably, we observed a relationship in which dominant mice were more sensitive to the behavioral effects of manipulating DRN DA activity (Figure 7). It might be expected that the quality of social interaction for a dominant animal may be very different from a subordinate, and thus, their subjective experience of social isolation may also differ. Therefore, in their representation of a loneliness-like state, changes in DRN DA activity may only exert a significant effect on the behavior of individuals who are engaged in positively valued social interactions. Importantly, this also suggests that DRN DA neurons are not merely indicating the removal of sensory stimuli by social isolation but actually representing the subjective experience of a loneliness-like state.

It remains to be determined whether underlying neural differences play a causal role in dictating social rank and/or whether social rank itself imposes a change in neuronal properties. In monkeys, the attainment of a dominant social rank increases striatal D_2/3_ receptor availability (Morgan et al., 2002), while in mice, altering synaptic efficacy in the mPFC is sufficient to promote a change in social rank (Wang et al., 2011). Furthermore, the observation that socially isolated and subordinate monkeys show similar D_2/3_ receptor availability (Morgan et al., 2002) supports the notion that subordinate animals may be in a loneliness-like state, even while group-housed. Therefore, this may be one reason why manipulations of DRN DA activity were not as effective in promoting behavioral adaptations in subordinate animals.

### Characterizing Components of the DRN DA Circuit

The relative bias of the DRN DA neurons in their projections to the BNST and CeA (Hasue and Shammah-Lagnado, 2002, Meloni et al., 2006) represents an important distinction from the VTA population and suggests these DA neurons are part of a distinct circuit. The BNST and the CeA have been implicated in diverse behavioral functions (Davis et al., 2010, Janak and Tye, 2015, Kim et al., 2013), and DA receptor signaling in these regions modulates synaptic transmission and activity (Kash et al., 2008, de la Mora et al., 2010). Given that the BNST has been associated with mediating long-term “tonic” behavioral responses to sustained, diffuse, and/or unpredictable threats, whereas the CeA is thought to be more important in the rapid, acute response to threatening stimuli (Davis et al., 2010), it is likely that both of these regions may be important in mediating the observed effects of DRN DA stimulation on behavior.

Indeed, 24 hr of social isolation results in a blunting of long-term potentiation (LTP) in the BNST (Conrad et al., 2011). Given that DA release in the BNST has been shown to facilitate LTP (Kash et al., 2008), we speculate that increased DA release following social isolation may occlude LTP. However, we also demonstrate that glutamate can be released with optical stimulation of the DRN DA neurons, and the neuropeptide vasoactive intestinal peptide (VIP) is co-expressed in a subset of DA neurons (Dougalis et al., 2012). Thus, DA, glutamate, VIP, or the coordinated activity of these three neurotransmitters/neuromodulators may be important in facilitating the output of the DRN DA neurons.

### Conclusion

Continued dissection of the neural mechanisms which govern social behavior is vital for the understanding and treatment of social impairments, which characterize many debilitating neuropsychiatric disorders. Our data present an additional element for consideration in the control of social behavior, and support a novel role for a relatively unstudied population of DA neurons in representing the experience of social isolation.

## Experimental Procedures

### Ex Vivo Electrophysiology

Brain slices (220 μm thick) containing the DRN or VTA were prepared from male heterozygous TH-GFP or Pitx3-GFP mice in order to target DA neurons. Whole-cell patch-clamp recordings were performed in voltage-clamp using a Multiclamp 700B amplifier (Molecular Devices) and Clampex 10.2 software (Molecular Devices). Afferent fibers were stimulated using a bipolar stimulating electrode (FHC) and glutamatergic currents were isolated by addition of picrotoxin (100 μM) to the ACSF.

### Fiber Photometry

TH::Cre mice received an injection of AAV_5_-CAG-FLEX-GCaMP6m into the DRN, and an optic fiber, held in a stainless steel ferrule, was implanted in the region. The photometry system was constructed similar to previously described (Gunaydin et al., 2014). GCaMP6m fluorescence was recorded while mice were in their home cage for 5 min before and after addition of a juvenile mouse or novel object.

### Fast-Scan Cyclic Voltammetry (FSCV)

Anesthetized in vivo FSCV experiments were conducted similar to those previously described (Tsai et al., 2009). TH::Cre mice, which had received an injection of AAV_5_-DIO-ChR2-eYFP into the DRN, were anesthetized with urethane and placed in a stereotaxic frame. Voltammetric recordings were collected, from either BNST or CeA, at 10 Hz by applying a triangular waveform (−0.4 V to +1.3 V to −0.4 V, 400 V/s) to a carbon-fiber electrode lowered into the region, versus an Ag/AgCl reference electrode.

### Behavioral Testing

TH::Cre and DAT::Cre mice received an injection of AAV_5_-DIO-ChR2-eYFP, AAV_5_-DIO-NpHR3.0-eYFP, or AAV_5_-DIO-eYFP into the DRN and were allowed at least 4 weeks for viral expression before behavioral testing. Mice were housed on a 12 hr:12 hr reverse light/dark cycle (lights off at 9:00 am) and tested during their active dark phase. Optical activation or inhibition was achieved by delivery of 473 nm light (30 Hz train of 8 pulses of 5 ms pulse width) or 593 nm light (constant), respectively, via an optic fiber secured in a stainless steel ferrule implanted over the DRN. For details of specific behavioral assays, see Supplemental Experimental Procedures.

## Author Contributions

G.A.M., M.A.U., and K.M.T. designed the experiments and wrote the paper. G.A.M. performed ex vivo electrophysiology, E.H.N. conducted photometry recordings, and C.M.V.W. conducted FSCV recordings. G.A.M. and E.H.N. performed stereotaxic surgery. G.A.M., S.A.H., A.S.Y., R.V.P, and G.D.L. conducted behavioral experiments. G.A.M., C.M.V.W., R.E.T., G.D.L., A.S.Y., G.F.G., E.M.I., R.V.P., and C.P.W. performed immunohistochemistry and analyzed data.

## Supplemental Information

Document S1. Supplemental Experimental Procedures

Movie S1. DRN DA Activity Increases upon Initial Social Contact after Social Isolation, Related to Figure 2 A TH::Cre mouse, expressing GCaMP6m in DRN DA neurons, was isolated for 24 hr and then recorded in his home cage during introduction of a novel juvenile mouse. The corresponding GCaMP6m fluorescence trace from fiber photometry recording shows increased activity in response to initial contact with the juvenile mouse.

Document S2. Article plus Supplemental Information
