# Supplementary material for: Dorsal Raphe Dopamine Neurons Represent the Experience of Social Isolation
Source: Cell. 2016 Feb 11;164(4):617–31. doi: 10.1016/j.cell.2015.12.040 (PMC4752823; doi:10.1016/j.cell.2015.12.040)
Supplement: Document S1. Supplemental Experimental Procedures [file mmc1.pdf]

Cell, Volume *164*

Supplemental Information

Dorsal Raphe Dopamine Neurons

# Represent the Experience of Social Isolation

Gillian A. Matthews, Edward H. Nieh, Caitlin M. Vander Weele, Sarah A. Halbert, Roma V. Pradhan, Ariella S. Yosafat, Gordon F. Glober, Ehsan M. Izadmehr, Rain E. Thomas, Gabrielle D. Lacy, Craig P. Wildes, Mark A. Ungless, and Kay M. Tye

SUPPLEMENTAL EXPERIMENTAL PROCEDURES

Methods (for all *ex vivo* recordings in Figures 1 and S1)

Animals and Manipulations

All breeding and experimental procedures were conducted under a project license approved by the Home Office, in accordance with the Animals (Scientific Procedures) Act of 1986 (United Kingdom). 1-4 month-old male, heterozygous tyrosine hydroxylase (TH)-GFP (Sawamoto et al., 2001) or pituitary homeobox 3 (Pitx3)-GFP (Zhao et al., 2004) mice were used to target dopamine (DA) neurons (see (Dougalis et al., 2012) for validation of these mice to target DA neurons in the dorsal raphe nucleus (DRN)). A subset of mice received a single dose of saline or vehicle (0.9% saline with 0.3% Tween-80), administered via intraperitoneal (IP) injection at a volume of 10 mL/kg. Mice were housed in a normal 12 h:12 h light-dark cycle (lights on at 7.00 am) and cage movements and/or injections were conducted at 10.00 am, 24 h before brain slice preparation.

Brain slice preparation

Mice were sacrificed by isoflurane anesthesia followed by decapitation. The brain was rapidly removed out of the cranial cavity and bathed in ice-cold (0-4 °C) artificial cerebrospinal fluid (ACSF, composition in mM, NaCl 120, KCl 3.5, NaH_2PO_4 1.25, $NaHCO_3$ 25, Glucose 10, $MgCl_2$ 1, $CaCl_2$ 2) fully equilibrated with carbogen gas (95% oxygen and 5% carbon dioxide). Two or three coronal brain slices (220 µm thickness) encompassing the DRN were obtained using a vibratome (Leica VT1000S, Leica

Microsystems, Germany). Slices were then maintained in a standard custom-made chamber and were gently and continuously aerated with carbogen gas for at least 1.5 h at room temperature (20-22 °C) before use for electrophysiology. For VTA recordings, two or three horizontal slices were prepared (220 μm thickness).

Electrophysiological recordings

Slices were transferred to a submersion recording chamber and were continuously perfused at a rate of 2-4 mL/min with fully oxygenated ACSF (containing 100 μM picrotoxin) at 30-32 °C. Neurons were visualized using infra-red differential interference contrast (IR-DIC) under an upright microscope (Olympus BXWI 51, Japan) equipped with a 40X objective (0.8 numerical aperture (NA)), an IR filter, DIC optics and a charge coupled device (CCD) video camera (Hamamatsu Photonics, Germany). Neurons were identified as GFP+ using fluorescence illumination (Xcite120 unit, EXFO, UK) coupled to a GFP excitation filter.

Whole-cell patch-clamp recordings were performed with a Multiclamp 700B amplifier (Molecular Devices, CA, USA) and Clampex 10.2 software (Molecular Devices, CA, USA) using glass microelectrodes (5–7 MΩ in resistance) filled with an internal solution containing (in mM): $CsCH_3SO_3$ 125, NaCl 2.8, HEPES 20, EGTA 0.4, TEA-Cl 5, MgATP 2, LiGTP 0.5, and 0.1% neurobiotin. Electrodes were pulled from thin-walled borosilicate glass capillary tubing (GC150F-10, Harvard Apparatus, UK) using a two-stage vertical puller (model PC-10, Narashige, Japan). Recordings were low-pass filtered at 1 kHz and digitized at 3-5 kHz. Series resistance (R_s) and input resistance (R_{in}) were frequently monitored throughout the experiments via a 10 mV, 250 ms

hyperpolarizing step. Any large changes in holding current or noise characteristics were taken as early signs of cell loss and recordings were terminated. Experiments were also terminated if R_s exceeded 35 MΩ or if R_{in} changed more than 15% after break in to whole-cell mode. All potentials cited here have not been corrected for liquid junction potentials (estimated using pClamp calculator as 9.2 mV).

In order to evoke postsynaptic currents, a bipolar stimulating electrode (FHC, USA) was placed 100-300 µm ventrolateral (in coronal slices) or rostral (in horizontal slices) to the recorded neuron, and used to stimulate afferents at 0.03 Hz. Stimulus intensity was controlled using an ISO-flex stimulus isolator (AMPI, Jerusalem, Israel) and adjusted to evoke monosynaptic events. Therefore stimulation only elicited currents with a single peak, and fast rise and decay kinetics. At least 12 stable sweeps were recorded for each protocol, with averaged responses calculated and analyzed using Clampfit 10.2 (Molecular Devices, CA, USA). To determine the AMPAR/NMDAR ratio, the neuron was voltage-clamped at +40 mV and a stable mixed AMPAR- and NMDAR-mediated excitatory postsynaptic current (EPSC) recorded. The NMDAR antagonist d-AP5 (50 µM; Tocris Bioscience, UK) was then applied to the slice, and the pure AMPAR current recorded. This was then digitally subtracted from the mixed current using ClampFit 10.2 to give the pure NMDAR current, and the AMPAR/NMDAR ratio was calculated by dividing the peak amplitude of the average AMPAR-mediated EPSC by the peak amplitude of the NMDAR-mediated EPSC. For analysis of correlation between AMPAR/NMDAR ratio and number of mice per cage, an average AMPAR/NMDAR ratio was calculated for each mouse.

For paired-pulse experiments an inter-stimulus interval of 50 ms was used, and paired stimuli were delivered every 30 s. The paired-pulse ratio (PPR) was calculated by dividing the average peak amplitude of the EPSC generated by the second stimulus by the average peak amplitude generated by the first. For rectification indices (RIs), and a subset of the PPRs, 0.1 mM spermine was included in the internal solution. To calculate the RI, 50 μM d-AP5 was added to the ACSF and the AMPAR current recorded while the neuron was voltage-clamped at -70 mV, 0 mV, and +40 mV. The RI was calculated by dividing the average peak of the AMPAR current recorded at -70 mV by the average peak recorded at +40 mV. NASPM (25 μM; Sigma, UK) was applied to the slice while stimulating EPSCs at -50 mV every 30 s. A stable baseline was recorded for at least 5 min followed by a 5 min application of NASPM. The average amplitude of the EPSC was calculated during the 5 min immediately prior to NASPM application (baseline) and the 5 min immediately following drug application, with the EPSC amplitude normalized to the baseline period. The decay kinetics of the NMDAR-mediated current recorded at +40 mV was determined by fitting the decay phase of the current with a double exponential function using Spike 2 (Cambridge Electronic Design, UK; Figure S1H). The following formula was used to calculate the weighted decay time constant (τ_W) of the NMDAR-mediated current:

$$\tau_W = [(A_1 \text{ x } \tau_1) + (A_2 \text{ x } \tau_2)]/[(A_1 + A_2)]$$

A_1 and A_2 are the amplitudes, and τ_1 and τ_2 the decay time constants of the fast and slow components, respectively (Lammel et al., 2011; Vicini et al., 1998).

Immunohistochemistry

Following recording brain slices were incubated in 4% w/v paraformaldehyde (PFA), in phosphate-buffered saline (PBS) overnight at 4 ºC (or 45 min at room temperature). Fixed free-floating sections were subsequently washed in PBS containing 0.2% Triton X-100 (PBS-T 0.2%), then blocked in PBS-T 0.2% with 6% normal donkey serum (NDS; Jackson ImmunoResearch, USA) for 30-60 min at room temperature. They were then incubated overnight at room temperature in PBS-T 0.2% containing 2% NDS and primary antibodies: anti-TH polyclonal chicken antibody (1:1000, Abcam ab76442, USA); anti-VIP (vasoactive-intestinal polypeptide) rabbit antibody (1:500, Immunostar 20077, USA). See (Dougalis et al., 2012) and references therein for discussion of validation of these antibodies. Brain slices were then washed 4 times in PBS-T 0.2% and incubated for 90 min at room temperature in PBS-T 0.2% containing 2% NDS and appropriate secondary antibodies: Cy3-conjugated anti-rabbit (1:1000, Jackson ImmunoResearch 711-165-152, USA) and Alexa Fluor 633-conjugated anti-chicken (1:1000, Invitrogen A21103, USA). AMCA-conjugated streptavidin (1:1000, Jackson ImmunoResearch 016-150-084, USA) was used to reveal neurobiotin labeling. Brain slices were finally washed 3 times in PBS-T 0.2%, followed by twice in PBS, then mounted onto glass microscope slides, and coverslipped using VectaShield mounting medium (Vector Laboratories, USA). Confocal laser scanning microscopy was performed using a Leica SP5 II confocal microscope (Leica Microsystems, Germany) through a 20X / 0.7 NA dry HC Plan-Apochromat CS DIC objective, with 1.5X digital zoom applied during image capture (30X total magnification). GFP was excited by a 488 nm line of an Argon laser, Cy3 by a 561 nm line of a DPSS laser, Alexa Fluor 633 by a

633 nm line of a HeNE laser, and AMCA by a 405 nm line of a diode laser. Images were captured at a resolution of 2048 x 2048 and were processed with general brightness and contrast curve adjustments in Adobe Photoshop CS5 (Adobe Systems Incorporated, CA, USA).

We observed a significant increase in the AMPAR/NMDAR ratio following 24 h of social isolation in GFP+ DRN neurons from TH-GFP and Pitx3-GFP mice (Figure S1A), and also in immunohistochemically-identified GFP+/TH+ and GFP+/TH- neurons (Figure S1B). We also found that both VIP+ and VIP- DRN DA neurons (Dougalis et al., 2012) displayed a significantly greater AMPAR/NMDAR ratio following social isolation compared with group-housed mice (Figures S1D and S1E). Furthermore, 24 h of social isolation significantly increased the AMPAR/NDMAR ratio in both adolescent (<postnatal day (PN)50) and adult (>PN50) mice (Figure S1C). Consequently, data from all GFP+ neurons, in 1-4 month-old mice from both transgenic lines, was pooled. Data presented in Figure S1A-E and Figure 7A-B includes all mice which either remained group-housed, or were socially isolated, including a subset of mice which received an IP injection of saline or vehicle 24 h prior to recording.

Statistics

Statistical analysis was performed using GraphPad Prism (GraphPad Software, Inc., CA, USA), and values are reported as mean ± standard error of the mean (S.E.M.). Two experimental groups were compared using an unpaired t-test (non-directional) or Mann-Whitney U test, and three or more groups were compared using a one-way ANOVA with Newman-Keuls post-hoc tests to control for multiple comparisons. The Pearson’s product-moment correlation coefficient was used to analyze correlation between two variables. Group sizes were based those used in previous studies to obtain significant differences using similar measures.

Methods (for all stereotaxic surgeries, FSCV, *ex vivo* recordings, behavioral testing, and fiber photometry in Figures 2-7 and S2-S7)

Animals

All experiments involving the use of animals were in accordance with NIH guidelines and approved by the MIT Institutional Animal Care and Use Committee. Male heterozygous TH::IRES-Cre (Lindeberg et al., 2004), DAT::IRES-Cre (Bäckman et al., 2006), VGLUT2::IRES-Cre (Vong et al., 2011), VGAT::IRES-Cre (Vong et al., 2011), and wild-type C57BL/6 mice (Jackson Laboratory, ME, USA) were housed on a 12 h: 12 h reverse light/dark cycle (lights off at 09.00 am) with food and water available *ad libitum*.

Stereotaxic Surgery

Surgery was performed on mice between 6-8 weeks of age. Mice were anesthetized with isoflurane (4% for induction, 1.5-2% thereafter for maintenance) and then placed in a small animal stereotax (David Kopf Instruments, CA, USA). All skull measurements were made relative to Bregma, unless otherwise noted. Viral injection into the DRN was performed using a beveled 33 gauge microinjection needle (facing medially) with a 10 µL microsyringe (Nanofil; WPI, FL, USA) delivering the virus at a rate of 0.1 µL/min using a microsyringe pump (UMP3; WPI, FL, USA) and controller (Micro4; WPI, FL, USA). Following viral injection, the needle was held at the injection site for 1-2 min, then raised up by 0.05 mm and held for a further 10 min to allow diffusion of the virus. The needle was then slowly withdrawn.

For behavioral experiments, 0.3-0.5 µL of the anterogradely travelling adeno-associated virus serotype 5 (AAV_5), encoding channelrhodopsin-2 (ChR2)-eYFP, under a double-floxed inverted open-reading frame construct (DIO) (AAV_5-EF1α-DIO-ChR2(H134R)-eYFP), or enhanced halorhodopsin 3.0 (AAV_5-EF1α-DIO-eNpHR3.0-eYFP) (Gradinaru et al., 2008), or eYFP alone (AAV_5-EF1α-DIO-eYFP) was injected into the DRN (at a 20° angle from the right side to avoid the aqueduct; AP: -4.10; ML: 1.25; DV: -2.90). For fiber photometry recordings, an AAV_5 encoding the calcium indicator, GCaMP6m, under a FLEX construct and the CAG promoter (AAV_5-CAG-FLEX-GCaMP6m) was injected into the DRN. In TH::Cre and DAT::Cre mice this resulted in eYFP expression in TH+ neurons within the DRN, ventrolateral periaqueductal grey, caudal linear and rostral linear nuclei. However, for *in vivo* manipulations or recording the optic fiber was always positioned over the DRN (Figures S2, S5, and S6).

In order to examine downstream release of glutamate in brain slices *ex vivo* and release of DA *in vivo*, TH::Cre and DAT::Cre mice received an injection of 0.3-0.5 µL AAV_5-EF1α-DIO-ChR2-eYFP into the DRN (AP: -4.10; ML: 1.25; DV: -2.90; at a 20° angle from the right side). Additionally, to determine co-expression of VGLUT2 or VGAT with TH in the DRN, 0.5 µL AAV_5-EF1α-DIO-ChR2-eYFP was injected into the DRN (AP: -4.10; ML: 1.25; DV: 2.90; at a 20° angle from the right side) of VGLUT2::Cre and VGAT::Cre mice.

For *in vivo* optogenetic manipulations, a manually-constructed optic fiber (300 µm core, NA=0.37 (Thorlabs, NJ, USA) (Sparta et al., 2012)) held in a stainless steel ferrule (Precision Fiber Products, CA, USA) was implanted directly above the DRN (AP: -4.05

to -4.10; ML: 0.00; DV: -2.40 to -2.45). For photometry experiments, an optic fiber was implanted within the DRN (AP: -3.90 to -4.20; ML: 0.00; DV: -2.50 to -2.80). A layer of adhesive cement (C&B Metabond; Parkell Inc., NY, USA) followed by cranioplastic cement (Ortho-Jet; Lang, IL, USA) was used to secure the optic fiber to the skull, which was allowed to dry completely before closure of the incision with nylon sutures. Mice were maintained under a heat lamp until they had fully recovered from anesthesia.

Viral constructs

Recombinant AAV vectors containing ChR2 or NpHR were serotyped with AAV_5 coat proteins and packaged by the University of North Carolina Vector Core (Chapel Hill, NC, USA). Viruses carrying GCaMP6m were packaged by the University of Pennsylvania Vector Core (Philadelphia, PA, USA).

Behavioral Experiments with Optogenetic Manipulations

Behavioral testing was performed at least 4 weeks following viral injection to allow sufficient time for transgene expression. Mice were tested during their active dark phase (09.00 am – 5:00 pm) and were given at least 1 h in the behavioral testing room to acclimate before experiments commenced. For experiments involving social isolation, mice were isolated between 9.00 am – 10.00 am and behavioral testing was performed 24-28 h later. Mice were isolated in their home cage or in a clean cage with some of the previous home cage bedding. Optic fiber implants were connected to a patch cable using a ceramic sleeve (PFP, CA, USA), which in turn was connected to a commutator (rotary joint; Doric, Québec, Canada) by means of an FC/PC adapter to allow

unrestricted movement. A second patch cable, with an FC/PC connector at either end (Doric, Québec, Canada), connected the commutator to a 473 nm or 593 nm diode-pumped solid state (DPSS) laser (OEM Laser Systems, UT, USA). A Master-8 pulse stimulator (A.M.P.I., Jerusalem, Israel) was used to control the output of the 473 nm laser using a light power of 20 mW, and the 593 nm laser using a light power of 1-5 mW.

Three-Chamber Sociability Test: Based on the three-chamber task design previously described (Felix-Ortiz and Tye, 2014; Moy et al., 2004), each experimental mouse was placed in a (57.15 x 22.5 x 30.5 cm) chamber consisting of four transparent Plexiglas walls and grey plastic floors. Within the chamber there were three compartments which the mice could move freely between: left and right compartments (each 24.5 x 22.5 cm) and a smaller center compartment (8 x 22.5 cm). The left and right compartments each contained an inverted wire cup. Mice were first subjected to a 10 min habituation period to acquaint them to the apparatus. Mice which displayed a strong preference for one side of the chamber (>70%) during this period were excluded from the analysis. Following habituation, the test mouse was contained within the center compartment for 1-2 min while a juvenile male C57BL/6 mouse (3.5-5 weeks of age) was placed under one of the two inverted wire cups (counterbalanced). The test mouse was then allowed to freely move between the compartments for a further 10 min. This test was repeated 24 h later using a different juvenile mouse and with the chamber rotated 90° to change external spatial cues. The 10 min phase in which the juvenile mouse was present was paired with optical stimulation (ChR2: 473 nm, 8 pulses of 5 ms pulse-width, at 30 Hz, every 5 s; NpHR: 593 nm, constant) on one of the two days

(counterbalanced across groups). A video camera located directly above the arena recorded each phase with mouse movement tracked using EthoVision XT (Noldus, Wageningen, Netherlands). Heat maps were generated in Ethovision XT with pseudo-color representing the relative time spent by the mouse at each position, with the maximum and minimum calculated within each trial. The social:non-social ratio was calculated by dividing the total time spent in the social zone (containing the juvenile mouse) by the time spent in the non-social zone.

Three-Chamber Novelty Preference Test: To test for novelty preference the three-chamber task was conducted (as described above for sociability), except instead of a juvenile mouse, a novel object was placed under one of the two inverted cups. The 10 min phase in the presence of the novel object was paired with optical stimulation (ChR2: 473 nm, 8 pulses of 5 ms pulse-width, at 30 Hz, every 5 s; NpHR: 593 nm, constant) on one of the two days (counter-balanced across groups).

Intra-Cranial Self-Stimulation: Mice were removed from *ad libitum* food 14-18 h prior to testing to facilitate behavioral responding. Single mice were placed in a Med Associates operant chamber (Med Associates, VT, USA) containing an active and inactive nose poke port, which were both illuminated. Low volume white noise was played into the chamber to mask extraneous sounds. A nose poke into either port was accompanied by illumination of a cue-light (positioned above the nose poke port) and a distinct 1 s tone (1 or 1.5 kHz) played into the box. A nose poke into the active port also resulted in delivery of a 30 Hz train of 5 ms pulse-width 473 nm light for 3 s, whereas a nose poke into the inactive port resulted in no stimulation. The physical location and associated tone of the nose poke ports were counterbalanced between groups. Mice

were allowed to explore the chamber for 2 h, with nose poke activity recorded with MedPC software (Med Associates, VT, USA), and subsequently analyzed using MATLAB (Mathworks, MA, USA). On the first training day, both operanda were baited with identical palatable odor cues to encourage investigation. On the second test day, the operanda were not baited.

Real-Time Place Aversion: Individual mice were placed in a transparent Plexiglas chamber (50 x 53 cm divided into left and right compartments, or 57.15 x 22.5 x 30.5 cm divided into left, right, and center compartments). They were allowed to freely move between compartments for 1 h, during which entry into one of the two sides resulted in photostimulation (473 nm light, 15 pulses of 5 ms pulse-width, at 30 Hz, every 5 s). The side paired with photostimulation was counterbalanced between animals. A video camera positioned directly above the arena was used to track mouse movement (EthoVision XT, Noldus, Wageningen, Netherlands). The first 30 min of exploration was used for analysis.

Conditioned-Place Preference: Single mice were placed in a (57.15 x 22.5 x 30.5 cm) chamber consisting of left and right compartments (each 24.5 x 22.5 cm) with rough or smooth grey plastic floors and a center compartment (8 x 22.5 cm) with a punched metal floor. Left and right compartment walls were either thin black and white vertical stripes or thick black and white horizontal stripes (counterbalanced across animals). On day 1, mice were exposed to a 15 min habituation phase in which they were allowed to freely explore the chamber. Mice with a strong initial preference for either side of the chamber (>70%) were excluded from the analysis. On day 2, mice were exposed to two 30 min conditioning sessions (separated by at least 2 h) during which they were

confined to one side of the chamber and received optical stimulation (15 pulses of 5 ms pulse-width 473 nm light, at 30 Hz, every 5 s) or no stimulation (counterbalanced for order and side across animals). On day 3, mice were placed in the chamber and allowed to freely explore in the absence of optical stimulation for 45 min, with the first 10 min used for analysis. A video camera positioned directly above the arena was used to track mouse movement (EthoVision XT, Noldus, Wageningen, Netherlands). Side preference was calculated by dividing the time spent in the compartment paired with optical stimulation by the total time spent in both compartments.

Open Field Test: Individual mice were placed in a 50 x 53 cm arena composed of four transparent Plexiglas walls. They were allowed to freely move throughout the arena for 15 min, with light stimulation occurring during the middle 5 min epoch (ChR2: 473 nm, 8 pulses of 5 ms pulse-width, at 30 Hz, every 5 s; NpHR: 593 nm, constant). A video camera positioned directly above the arena was used to track the movement of each mouse (EthoVision XT, Noldus, Wageningen, Netherlands). In order to assess anxiety-related behavior (Carola et al., 2002), the chamber was divided into a center (36 x 37 cm) and periphery region.

Elevated Plus Maze: The maze was elevated from the ground by 75 cm and consisted of two closed arms (30 x 5 x 30 cm) and two open arms (30 x 5 cm), emanating at 90° from each other from a central platform (5 x 5 cm), all made of grey plastic. Mice were placed on one of the open arms of the maze and allowed to freely explore for 15 min, with light stimulation occurring during the middle 5 min epoch (ChR2: 473 nm, 8 pulses of 5 ms pulse-width, at 30 Hz, every 5 s; NpHR: 593 nm,

constant). A video camera positioned directly above the arena was used to track the movement of each mouse (EthoVision XT, Noldus, Wageningen, Netherlands).

Resident-Intruder Assay: Mice were individually recorded in their home cage for a total of 9 min. After the first 2 min, photostimulation (ChR2: 473 nm, 8 pulses of 5 ms pulse-width, at 30 Hz, every 5 s; NpHR: 593 nm, constant) commenced. After a further 3 min, a juvenile mouse (3.5-4.5 weeks of age) was placed in the cage with the test mouse. Following 3 min of interaction photostimulation ceased and 1 min later the juvenile was removed. The behavior of the test mouse across the 3 min immediately following introduction of the juvenile intruder was manually scored twice by two different experimenters, blind to the experimental conditions, using ODLog behavioral analysis software (Macropod Inc., USA). The resident-intruder assay was performed twice, 24 h apart, with a different juvenile mouse used on each day, and with one day paired with photostimulation (order counterbalanced across groups). Notably, this assay was designed to examine anxiety-related behavior (File and Seth, 2003) rather than social interest, as the addition of an intruder to the home cage of a male may be interpreted as a threat.

Estimated Social Rank: In ChR2-expressing TH::Cre mice, relative social dominance was estimated based on relative body weight within the cage, whereby the heaviest mouse within a given cage was assigned a relative dominance score of ‘1’, the lightest mouse a score of ‘0’, and intermediate mice a score between 0-1 based on their relative weight. In addition, in a subset of mice, the tube test for social dominance was performed, similar to previously described (Lindzey et al., 1961; Wang et al., 2011). A transparent Plexiglas tube, 30 cm in length and 3.2 cm or 3.8 cm inside diameter

(dependent on mouse size) was used, which was sufficient to allow a single mouse to pass through uninhibited. Mice were exposed to 4 days of training, with eight trials on days 1-2 and 3 trials on days 3-4. For training trials, mice were released into the tube, at alternating ends, and allowed to run through and exit at the opposite end. Mice which were reluctant to pass all the way through, or attempted to reverse, were encouraged with a plastic stick gently pressing on their hind region. Following training, mice were then tested against each of their cagemates, in a round-robin design, each day for three consecutive days. For each test trial, two mice were simultaneously released at either end of the tube, and the mouse which was the first to retreat from the tube was designated as the 'loser', while his opponent was designated as the 'winner'. Trials in which the mice did not meet in the center of the tube were repeated. The order in which mice were tested against each other and the side from which they were released were counterbalanced across each day of trials. The relative proportion of 'wins' was calculated for each mouse across the three days of testing, by taking the number of 'wins' divided by the total number of contests. For NpHR-expressing mice, and a subset of ChR2-expressing mice, the proportion of tube test 'wins' was taken into account, in addition to relative body weight, so that their relative dominance score reflected an average of both of these measures.

Fiber Photometry

For the photometry system, blue light from a 473 nm DPSS laser (60-160 µW; OEM Laser Systems, UT, USA) was filtered through a neutral density filter (1.0 optical density, Thorlabs, NJ, USA) held in a filter wheel (FW1A, Thorlabs, NJ, USA), chopped

at 400±10 Hz (Model SR540 Chopper Controller, Stanford Research Systems, CA, USA) through a filter (LD01-473, Semrock, NY, USA) and reflected off of a dichroic mirror (FF495, Semrock, NY, USA) and coupled through a fiber collimation package (F240FC-A, Thorlabs, NJ, USA) into a patch cable connected to the implanted ferrule via a ceramic sleeve (PFP, CA, USA). GCaMP6m fluorescence was collected through a 525 nm bandpass filter (FF03-525, Semrock, NY, USA) into a photodetector (Model 2151, Newport, CA, USA). The signal was passed through a lock-in amplifier (100 ms, 12 dB, 500 mV, Model SR810, Stanford Research Systems, CA, USA) and digitized and collected with a LabJack U6-PRO (250 Hz sampling frequency, LabJack, CO, USA). The raw signal over the entire session was divided by a linear fit to normalize the baseline over the trial. Z-scores were taken using the period -5 to -2 s prior to the event detections as baseline.

Ethovision XT (Noldus, Wageningen, Netherlands) was used to collect video footage of the test mouse, and a TTL pulse was sent to the LabJack U6-PRO to time-lock the video with the photometry data. Mice (either group-housed or socially isolated for 24 h) were placed in their home cage and fluorescence was recorded for 5 min, after which a juvenile intruder or novel object was placed into the cage. Bouts of interaction with either the juvenile intruder or novel object were scored manually by experimenters blind to the experimental conditions.

Fast-Scan Cyclic Voltammetry (FSCV)

TH::Cre mice, which had received an injection of 0.3-0.5 µL AAV_5-DIO-ChR2-eYFP in the DRN, were given at least 8 weeks for viral expression before recording experiments. Anesthetized *in vivo* FSCV experiments were conducted similar to those

previously described (Tsai et al., 2009). Briefly, mice were anesthetized with urethane (1.5 g/kg; IP) and placed in a stereotaxic frame. Craniotomies were performed above the BNST (AP: +0.20 mm, ML: +1.00 mm), CeA (AP: -0.75 mm, ML: +2.35 mm), DRN (AP: -4.15, ML: 0.00 mm), and contralateral cortex. An Ag/AgCl reference electrode was implanted in the contralateral cortex and a 300 µm optical fiber was implanted above the DRN (DV: - 2.45 mm). Both implants were secured to the skull with adhesive cement (C&B Metabond; Parkell, NY, USA). A glass-encased carbon fiber electrode (~120 µm in length, epoxied seal) was lowered into the BNST (DV: -3.00 mm from brain surface) or CeA (DV: -3.20 mm from brain surface) for electrochemical recordings. Electrodes were allowed to equilibrate for 20 min at 60 Hz and 10 min at 10 Hz. Voltammetric recordings were collected at 10 Hz by applying a triangular waveform (-0.4 V to +1.3 V to -0.4 V, 400 V/s) to the carbon-fiber electrode versus the Ag/AgCl reference. DA release was evoked by optical stimulation of the DRN using 150 pulses of 473 nm light (25 mW, 5 ms pulse duration) at 20, 30, or 50 Hz, delivered via a DPSS laser (OEM Laser Systems, UT, USA) and controlled using a Master-8 pulse stimulator (A.M.P.I., Jerusalem, Israel). Data were collected using Tarheel CV (NC, USA) in 15 s files with the stimulation onset occurring 5 s into the file. Files were collected every 60 s and background subtracted at the lowest current value prior to stimulation onset. Light-evoked signals maintained characteristic cyclic voltammograms for DA (unless otherwise noted in text), with oxidation and reduction peaks at ~+0.65 V and ~-0.2 V, respectively.

A group of 10 carbon-fiber electrodes were calibrated in known concentrations of DA (250 nM, 500 nM, and 1 µM) and mean calibration data were used to convert *in vivo*

signals to changes in DA concentration using chemometric, principal component regression, and residual analyses using a custom LabView program (provided by R. Keithley). Following recordings, mice were transcardially perfused with 4% PFA (described below) and processed using immunohistochemistry to confirm viral expression and placement of the optic fiber and recording electrodes. Stimulation recordings collected at 10 Hz were individually binned in 0.5 s bins for visualization. Evoked DA release was quantified by calculating the area under the curve and peak evoked release for each recording.

Ex Vivo Electrophysiological Recordings in the BNST and CeA

TH::Cre or DAT::Cre mice received an injection of 0.3-0.5 µL AAV_5-DIO-ChR2-eYFP into the DRN and at least 8 weeks was allowed for viral expression. Mice were deeply anesthetized with sodium pentobarbital (200 mg/kg; IP) then transcardially perfused with 20 mL ice-cold modified ACSF (composition in mM: NaCl 87, KCl 2.5, NaH2PO4*H20 1.3, MgCl2*6H2O 7, NaHCO3 25, sucrose 75, ascorbate 5, CaCl2*2H2O 0.5, in ddH20; osmolarity 320-330 mOsm, pH 7.30-7.40) saturated with carbogen gas (95% oxygen, 5% carbon dioxide). The brain was rapidly removed from the cranial cavity and sectioned on a vibrating-blade microtome (Leica VT1000S, Leica Microsystems, Germany). Coronal 300 µm brain slices were prepared containing the DRN, the BNST, and the CeA. Slices were given at least 1.5 h to recover in a holding chamber containing ACSF (composition in mM: NaCl 126, KCl 2.5; NaH2PO4*H20 1.25, MgCl2*6H2O 1, NaHCO3 26, glucose 10, CaCl2*2H2O 2.4, in ddH20; osmolarity 299-301 mOsm; pH 7.30-7.40) saturated with carbogen gas at 32 °C before being transferred to the recording chamber for electrophysiology. In the recording chamber,

slices were continuously perfused at a rate of 2 mL/min with fully oxygenated ACSF at 30-32 °C.

For whole-cell patch-clamp electrophysiology, electrodes were pulled from thin-walled borosilicate glass capillary tubing on a P-97 puller (Sutter Instrument, CA, USA) and had resistances of 4-7 MΩ when filled with internal solution (composition in mM: $CsCH_3SO_3$ 117, NaCl 2.8, HEPES 20, EGTA 0.4, TEA-Cl 5, MgATP 4, Na-GTP 0.3, QX-314 5, spermine 0.1, and 0.3% biocytin, in ddH20; osmolarity 287 mOsm; pH 7.30). Recordings were made using a Multiclamp 700B amplifier and Clampex 10.4 software (Molecular Devices, CA, USA). Signals were low-pass filtered at 1 kHz and digitized at 10 kHz using a Digidata 1550 (Molecular Devices, CA, USA). Throughout recording, capacitance, R_s, and R_{in} were frequently measured to monitor cell health. Neurons were visualized via a 40X water-immersion objective on an upright microscope (Scientifica, UK) equipped with IR-DIC optics and a Q-imaging Retiga Exi camera (Q Imaging, Canada). The region of terminal expression in the BNST and CeA was identified by brief illumination through a 470 nm LED light source (pE-100; CoolLED, NY, USA). Neurons were recorded in voltage-clamp mode with a holding potential of -70 mV and 0 mV to elicit EPSCs and IPSCs, respectively. ChR2 was activated by a 5 ms pulse of 470 nm LED light, delivered through the objective, every 20 s. In order to confirm the existence of a monosynaptic connection, in a subset of cells, the optically-evoked current was recorded in the presence of tetrodotoxin (TTX; 1 μM) and 4-aminopyridine (4AP; 0.5-1 mM) (Petreanu et al., 2009).

For recording ChR2-expressing neurons within the DRN, an internal solution was used containing (in mM): potassium gluconate 125, NaCl 10, HEPES 20, MgATP 3, and

0.3% biocytin, in ddH20 (osmolarity 287 mOsm; pH 7.33). Neurons were recorded in current-clamp mode and ChR2 was activated by 8 pulses of 470 nm LED light (5 ms pulse duration) delivered at 30 Hz every 5 s.

Subsequent analysis was performed using Clampfit 10.4 software (Molecular Devices, CA, USA) with at least 12 sweeps used to calculate the average light-evoked EPSC. Amplitude and latency were estimated in Clampfit, with latency taken as the time from light onset to the initial downward deflection of the EPSC.

Immunohistochemistry

Mice were deeply anesthetized with sodium pentobarbital (200 mg/kg; IP) and then transcardially perfused with 20 mL ice-cold (~4 °C) Ringers solution followed by 20 mL ice-cold 4% PFA in PBS. The brain was then dissected out of the cranial cavity and placed in 4% PFA solution at 4 °C for 5-18 h before being transferred to 30% sucrose solution for at least 36 h at 4 °C. Brains were sectioned at 40 µm thickness on a freezing microtome (HM430; Thermo Fisher Scientific, MA, USA) and sections were subsequently stored in PBS at 4 °C. For immunohistochemistry sections were blocked in PBS-T 0.3% with 3% NDS (Jackson ImmunoResearch, USA) for 1 h at room temperature followed by incubation in primary antibody solution: chicken anti-TH (1:1000; Millipore, USA) and rabbit anti-5-HT (1:2000; Immunostar, WI, USA) in PBS-T 0.3% with 3% NDS for 48 h at 4 °C. Sections were subsequently washed 4 times in PBS (for 10 min each) and then transferred to secondary antibody solution: Alexa 647-conjugated donkey anti-chicken (1:500-1:1000), Cy3-conjugated donkey anti-rabbit (1:1000), and a DNA-specific fluorescent probe (DAPI; 1:50,000) in PBS-T 0.1% with

3% NDS for 2 h at room temperature. Sections were again washed 4 times in PBS (for 10 min each) before being mounted onto glass slides and coverslipped using polyvinyl alcohol mounting medium with DABCO (Sigma, MO, USA).

Confocal Microscopy

Fluorescent images were captured using a confocal laser scanning microscope (Olympus FV1000), with FluoView software (Olympus, PA, USA), under a 10X / 0.40 NA dry objective or a 40X /1.30 NA oil immersion objective. The location of viral expression at the injection site, the lesion from the optic fiber placement, and the position of carbon-fiber recording electrodes were determined by taking serial z-stack images through the 10X objective across a depth of at least 20 μm, with an optical slice thickness of 5 μm. High magnification images of eYFP-expressing cells under the optic fiber tip were obtained through the 40X objective using serial z-stack images with an optical slice thickness of 2 μm. Images were subsequently processed in Adobe Photoshop CS6 (Adobe Systems Incorporated, CA, USA).

Immunohistochemical Quantification

At least 4 weeks following viral injection, VGLUT2::Cre and VGAT::Cre mice were transcardially perfused-fixed and the brains processed using immunohistochemistry (as described above). High magnification images of eYFP+ cells in the DRN were obtained, within the region containing TH+ neurons. Two VGLUT2::Cre and two VGAT::Cre mice were analyzed using 2 sections per animal. The numbers of TH+ and eYFP+/TH+ neurons within the region were counted by an experimenter blind to the experimental condition.

In TH::Cre and DAT::Cre mice the proportion of eYFP+ cells co-labeled with TH in the DRN was determined from counts performed on 1-2 sections per animal, in which the lesion from the tip of the optic fiber was visible (n=8 mice for TH::Cre and n=6 mice for DAT::Cre). In order to determine eYFP/5-HT co-expression one anterior and one posterior section containing the DRN was counted from each animal (n=3 TH::Cre and n=3 DAT::Cre). Cell counting was performed using custom written software in MATLAB (Mathworks, Natick, MA, USA).

Statistics

Statistical analyses were performed using using GraphPad Prism (GraphPad Software, Inc., CA, USA), OriginPro 8.6 (OriginLab, MA, USA), and MATLAB (Mathworks, MA, USA). Group comparisons were made using two-way repeated measures ANOVA followed by Bonferroni post-hoc tests to control for multiple comparisons. Paired and unpaired t-tests, as well as one-way repeated measures ANOVAs were used to make single-variable comparisons, and the Chi-squared test was used to compare populations. The Pearson's product-moment correlation coefficient or the Spearman's rank correlation coefficient was used to analyze correlation between two variables.

SUPPLEMENTAL REFERENCES

Bäckman, C.M., Malik, N., Zhang, Y., Shan, L., Grinberg, A., Hoffer, B.J., Westphal, H., and Tomac, A.C. (2006). Characterization of a mouse strain expressing Cre recombinase from the 3' untranslated region of the dopamine transporter locus. Genes. N. Y. N 2000 *44*, 383–390.

Carola, V., D'Olimpio, F., Brunamonti, E., Mangia, F., and Renzi, P. (2002). Evaluation of the elevated plus-maze and open-field tests for the assessment of anxiety-related behaviour in inbred mice. Behav. Brain Res. *134*, 49–57.

Dougalis, A.G., Matthews, G.A.C., Bishop, M.W., Brischoux, F., Kobayashi, K., and Ungless, M.A. (2012). Functional properties of dopamine neurons and co-expression of vasoactive intestinal polypeptide in the dorsal raphe nucleus and ventro-lateral periaqueductal grey. Eur. J. Neurosci. *36*, 3322–3332.

Felix-Ortiz, A.C., and Tye, K.M. (2014). Amygdala inputs to the ventral hippocampus bidirectionally modulate social behavior. J. Neurosci. Off. J. Soc. Neurosci. *34*, 586–595.

File, S.E., and Seth, P. (2003). A review of 25 years of the social interaction test. Eur. J. Pharmacol. *463*, 35–53.

Gradinaru, V., Thompson, K.R., and Deisseroth, K. (2008). eNpHR: a Natronomonas halorhodopsin enhanced for optogenetic applications. Brain Cell Biol. *36*, 129–139.

Lammel, S., Ion, D.I., Roeper, J., and Malenka, R.C. (2011). Projection-specific modulation of dopamine neuron synapses by aversive and rewarding stimuli. Neuron *70*, 855–862.

Lindeberg, J., Usoskin, D., Bengtsson, H., Gustafsson, A., Kylberg, A., Söderström, S., and Ebendal, T. (2004). Transgenic expression of Cre recombinase from the tyrosine hydroxylase locus. Genes. N. Y. N 2000 *40*, 67–73.

Lindzey, G., Winston, H., and Manosevitz, M. (1961). Social Dominance in Inbred Mouse Strains. Nature *191*, 474–476.

Moy, S.S., Nadler, J.J., Perez, A., Barbaro, R.P., Johns, J.M., Magnuson, T.R., Piven, J., and Crawley, J.N. (2004). Sociability and preference for social novelty in five inbred strains: an approach to assess autistic-like behavior in mice. Genes Brain Behav. *3*, 287–302.

Sawamoto, K., Nakao, N., Kobayashi, K., Matsushita, N., Takahashi, H., Kakishita, K., Yamamoto, A., Yoshizaki, T., Terashima, T., Murakami, F., et al. (2001). Visualization, direct isolation, and transplantation of midbrain dopaminergic neurons. Proc. Natl. Acad. Sci. *98*, 6423–6428.

Sparta, D.R., Stamatakis, A.M., Phillips, J.L., Hovelsø, N., van Zessen, R., and Stuber, G.D. (2012). Construction of implantable optical fibers for long-term optogenetic manipulation of neural circuits. Nat. Protoc. *7*, 12–23.

Tsai, H.-C., Zhang, F., Adamantidis, A., Stuber, G.D., Bonci, A., de Lecea, L., and Deisseroth, K. (2009). Phasic firing in dopaminergic neurons is sufficient for behavioral conditioning. Science *324*, 1080–1084.

Vicini, S., Wang, J.F., Li, J.H., Zhu, W.J., Wang, Y.H., Luo, J.H., Wolfe, B.B., and Grayson, D.R. (1998). Functional and pharmacological differences between recombinant N-methyl-D-aspartate receptors. J. Neurophysiol. *79*, 555–566.

Vong, L., Ye, C., Yang, Z., Choi, B., Chua, S., and Lowell, B.B. (2011). Leptin Action on GABAergic Neurons Prevents Obesity and Reduces Inhibitory Tone to POMC Neurons. Neuron *71*, 142–154.

Wang, F., Zhu, J., Zhu, H., Zhang, Q., Lin, Z., and Hu, H. (2011). Bidirectional Control of Social Hierarchy by Synaptic Efficacy in Medial Prefrontal Cortex. Science *334*, 693–697.

Zhao, S., Maxwell, S., Jimenez-Beristain, A., Vives, J., Kuehner, E., Zhao, J., O'Brien, C., de Felipe, C., Semina, E., and Li, M. (2004). Generation of embryonic stem cells and transgenic mice expressing green fluorescence protein in midbrain dopaminergic neurons. Eur. J. Neurosci. *19*, 1133–1140.
