# Supplementary figures and images for: Dorsal Raphe Dopamine Neurons Represent the Experience of Social Isolation
Source: Cell. 2016 Feb 11;164(4):617–31. doi: 10.1016/j.cell.2015.12.040 (PMC4752823; doi:10.1016/j.cell.2015.12.040)

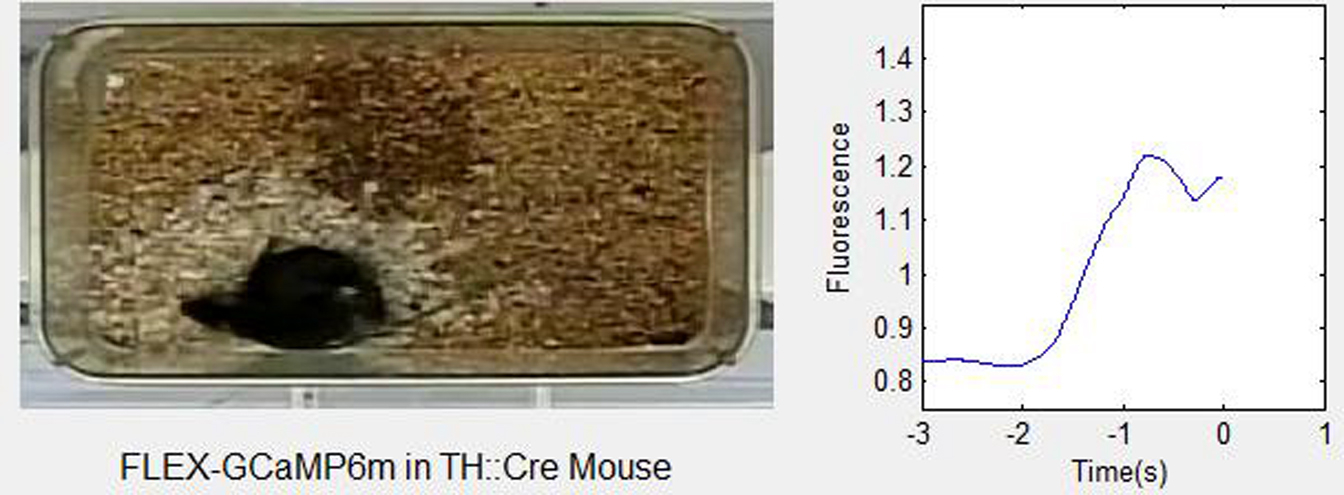

Supplement: Movie S1. DRN DA Activity Increases upon Initial Social Contact after Social Isolation, Related to Figure 2 — A TH::Cre mouse, expressing GCaMP6m in DRN DA neurons, was isolated for 24 hr and then recorded in his home cage during introduction of a novel juvenile mouse. The corresponding GCaMP6m fluorescence trace from fiber photometry recording shows increased activity in response to initial contact with the juvenile mouse. [file mmc2.jpg]
