# Supplementary material for: Dorsal Raphe Dopamine Neurons Represent the Experience of Social Isolation
Source: Cell. 2016 Feb 11;164(4):617–31. doi: 10.1016/j.cell.2015.12.040 (PMC4752823; doi:10.1016/j.cell.2015.12.040)
Supplement: Document S2. Article plus Supplemental Information [file mmc3.pdf]

Article

Cell

Dorsal Raphe Dopamine Neurons Represent the Experience of Social Isolation

Graphical Abstract

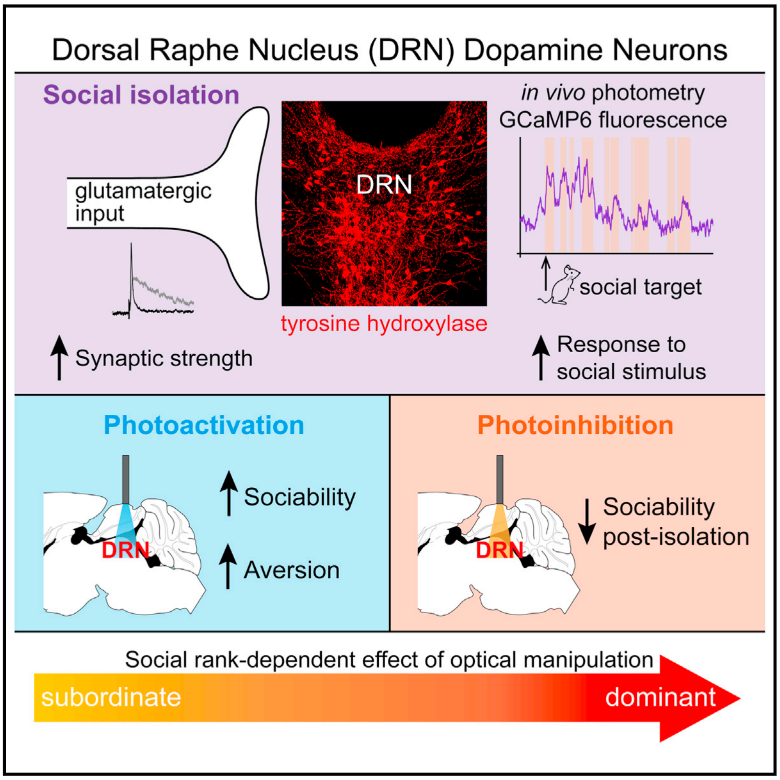

Highlights

- Dorsal raphe nucleus (DRN) dopamine neurons are sensitive to acute social isolation
- DRN dopamine neurons release dopamine and glutamate in downstream structures
- Optical activation induces, whereas inhibition suppresses, a “loneliness-like” state
- Social rank predicts the behavioral effect induced by optical manipulations

Authors

Gillian A. Matthews, Edward H. Nieh, Caitlin M. Vander Weele, ..., Craig P. Wildes, Mark A. Ungless, Kay M. Tye

Correspondence

mark.ungless@imperial.ac.uk (M.A.U.), kaytye@mit.edu (K.M.T.)

In Brief

Dopamine neurons within the dorsal raphe nucleus are sensitive to acute social isolation, and are able to modulate a “loneliness-like” state upon optical stimulation. These neurons may underlie the subjective experience of social isolation as well as the motivational drive to re-engage in social connections.

Matthews et al., 2016, Cell *164*, 617–631
February 11, 2016 ©2016 The Authors
http://dx.doi.org/10.1016/j.cell.2015.12.040

CellPress

Cell

Dorsal Raphe Dopamine Neurons Represent the Experience of Social Isolation

Gillian A. Matthews,[1,2,3] Edward H. Nieh,[1,3] Caitlin M. Vander Weele,[1,3] Sarah A. Halbert,[1] Roma V. Pradhan,[1] Ariella S. Yosafat,[1] Gordon F. Glober,[1] Ehsan M. Izadmehr,[1] Rain E. Thomas,[1] Gabrielle D. Lacy,[1] Craig P. Wildes,[1] Mark A. Ungless,[2,4,*] and Kay M. Tye[1,4,*]
[1]The Picower Institute for Learning and Memory, Department of Brain and Cognitive Sciences, Massachusetts Institute of Technology, Cambridge, MA 02139, USA
[2]Medical Research Council Clinical Sciences Centre, Imperial College London, Hammersmith Hospital, Du Cane Road, W12 0NN London, UK
[3]Co-first author
[4]Co-senior author
*Correspondence: mark.ungless@imperial.ac.uk (M.A.U.), kaytye@mit.edu (K.M.T.)
http://dx.doi.org/10.1016/j.cell.2015.12.040
This is an open access article under the CC BY license (http://creativecommons.org/licenses/by/4.0/).

SUMMARY

The motivation to seek social contact may arise from either positive or negative emotional states, as social interaction can be rewarding and social isolation can be aversive. While ventral tegmental area (VTA) dopamine (DA) neurons may mediate social reward, a cellular substrate for the negative affective state of loneliness has remained elusive. Here, we identify a functional role for DA neurons in the dorsal raphe nucleus (DRN), in which we observe synaptic changes following acute social isolation. DRN DA neurons show increased activity upon social contact following isolation, revealed by in vivo calcium imaging. Optogenetic activation of DRN DA neurons increases social preference but causes place avoidance. Furthermore, these neurons are necessary for promoting rebound sociability following an acute period of isolation. Finally, the degree to which these neurons modulate behavior is predicted by social rank, together supporting a role for DRN dopamine neurons in mediating a loneliness-like state.

INTRODUCTION

The establishment and maintenance of social bonds is crucial for survival of a social species. A social group offers safety and security, supports offspring survival, reduces the need for energy expenditure, and provides a stage for social reward (Eisenberger, 2012). The motivation to initiate and maintain social bonds may be rooted in emotional states of either positive or negative valence. Social interactions can be rewarding and thereby recruit components of the brain's reward circuitry, including the ventral tegmental area (VTA) dopamine (DA) neurons and the nucleus accumbens (NAc) (Dölen et al., 2013; Gunaydin et al., 2014; Robinson et al., 2002).

Conversely, the absence of social contact also triggers a strong desire to seek social interaction. Social isolation, social exclusion, or feelings of social disconnection can lead to loneliness, which is a strongly aversive emotional state in humans and detrimental to physical and mental well-being (Cacioppo et al., 2006, 2014; Holt-Lunstad et al., 2010; House et al., 1988). The aversive nature of this state is emphasized by the controversial use of solitary confinement as a form of punishment (Browne et al., 2011; Walker et al., 2014). Therefore, the negative state of isolation can trigger the motivation to seek and engage in social contact (Baumeister and Leary, 1995; Maner et al., 2007; Williams and Sommer, 1997), perhaps as an evolutionarily conserved mechanism to maintain social connections (Buss, 1990).

Social isolation is also aversive to rodents. Rodents are innately social creatures and prefer social rather than isolate housing (Loo et al., 2001). Even an acute period of isolation in rodents increases motivation to seek out and engage with conspecifics (Niesink and van Ree, 1982; Panksepp and Beatty, 1980). However, little is known about how this isolation-induced state is represented at a neural level.

Given that the mesolimbic DA system has been implicated in social behavior (Gunaydin et al., 2014; Puglisi-Allegra and Cabib, 1997; Robinson et al., 2002) and that perturbations in DA signaling have been reported following chronic social isolation (Hall et al., 1998), we initially considered VTA DA neurons as a candidate neural substrate for social isolation. However, since optogenetic activation of VTA DA neurons increases social interaction (Gunaydin et al., 2014) and supports positive reinforcement (Tsai et al., 2009; Witten et al., 2011), they are thought to play a causal role in social reward.

Given that DA neurons are functionally heterogeneous (Brischoux et al., 2009; Lammel et al., 2012), we investigated a relatively neglected subpopulation of DA neurons in the dorsal raphe nucleus (DRN). Amid the sparse existing knowledge of the functional role of DRN DA neurons, optical stimulation of these neurons does not support intra-cranial self-stimulation (ICSS) (McDevitt et al., 2014), in contrast to the VTA (Witten et al., 2011), suggesting that DRN and VTA DA neurons may be functionally distinct.

Here, we investigated the functional role of DRN DA neurons, which we found to possess the properties expected of a neural substrate for a "loneliness-like" state. Specifically, the strength

CrossMark

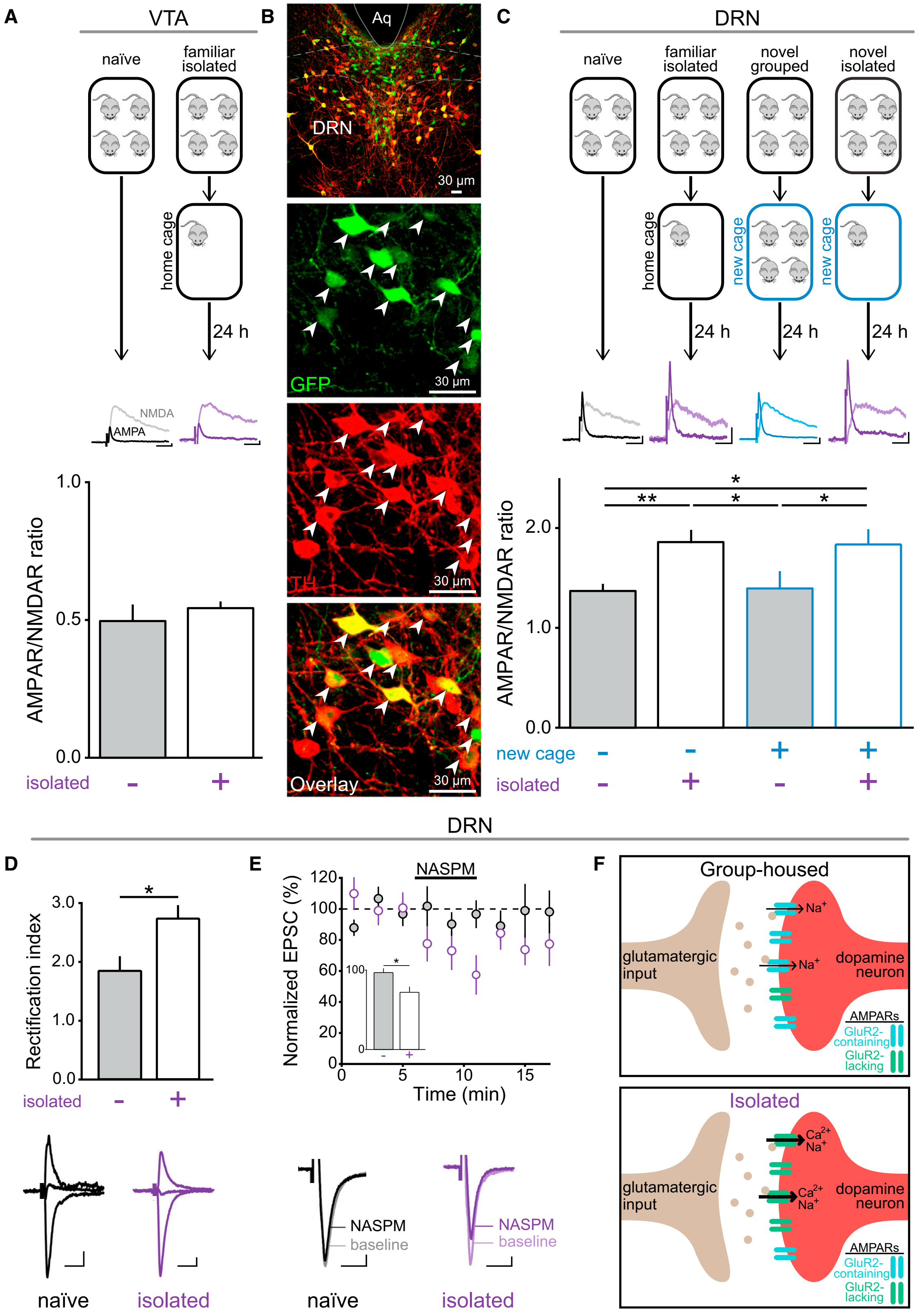

(legend on next page)

of excitatory inputs onto DRN DA neurons and their naturally occurring activity in vivo were sensitive to social isolation. Optical activation of these neurons recapitulated a loneliness-like state, while optical inhibition prevented the sociability typically observed following a period of isolation. Furthermore, the magnitude of these effects was predicted by an individual's social rank, which indicates the importance of prior social experience in determining the behavioral effect governed by these neurons. Taken together, we propose that DRN DA neurons represent a neural substrate for the subjective experience of social isolation and serve to promote a response to alleviate this aversive state.

RESULTS

Acute Social Isolation Potentiates Synapses onto DA Neurons in the DRN, but Not the VTA

In order to probe the effect of social isolation on glutamatergic synaptic strength, we used whole-cell patch-clamp electrophysiology in brain slices prepared from male mice expressing GFP in DA neurons (Figures S1A–S1E; Supplemental Experimental Procedures) and measured the α-amino-3-hydroxy-5-methyl-4-isoxazolepropionic acid receptor (AMPAR)/N-methyl-D-aspartate receptor (NMDAR) ratio. Glutamatergic synapses onto VTA DA neurons undergo rapid changes in synaptic strength within 24 hr of an acute appetitive (Ungless et al., 2001) or aversive experience (Lammel et al., 2011; Saal et al., 2003). We therefore considered whether 24 hr of social isolation could induce potentiation at these synapses. However, we did not detect a difference in AMPAR/NMDAR ratio between group-housed and socially isolated mice in VTA DA neurons (Figure 1A).

Among the relatively unexplored subpopulations of DAergic neurons residing outside of the VTA, an intriguing group lies within the DRN (Figure 1B) (Hökfelt et al., 1976), which is highly conserved across species (Saper and Petito, 1982). Strikingly, DRN DA neurons in mice that were socially isolated for 24 hr exhibited a significantly greater AMPAR/NMDAR ratio than group-housed, naive mice (Figure 1C). In order to confirm this effect was related to the experience of social isolation, rather than a nonspecific salient environmental manipulation, we also examined movement into a new cage. We found that transfer into a new cage as a group had no detectable effect on AMPAR/NMDAR ratio, but social isolation in a new cage also increased the AMPAR/NMDAR ratio (Figure 1C).

Social Isolation Changes Receptor Composition at Synapses onto DRN DA Neurons

To examine the mechanism of social isolation-induced plasticity in DRN DA neurons, we used additional electrophysiological measures. Postsynaptically, AMPAR subunit composition can influence neuron excitability and synaptic efficacy (Liu and Zukin, 2007). In contrast to GluR2-containing AMPARs, GluR2-lacking receptors are Ca^{2+}-permeable and exhibit higher single-channel conductance (Hollmann et al., 1991; Swanson et al., 1997). We found that the rectification index (RI) of the AMPAR current was significantly greater in socially isolated mice (Figure 1D), suggesting an increase in GluR2-lacking AMPARs, which exhibit a characteristic inwardly rectifying current at positive potentials (Bellone and Lüscher, 2006; Liu and Zukin, 2007). At resting membrane potentials, the polyamine spermine can partially block GluR2-lacking AMPARs (Bowie and Mayer, 1995), but opening of the receptor temporarily relieves this blockade, which results in a greater response to subsequent stimulation (Rozov and Burnashev, 1999). This therefore promotes paired-pulse facilitation at GluR2-lacking synapses (Liu and Zukin, 2007). Indeed, we found that the paired-pulse ratio (PPR) in the presence of spermine was greater in socially isolated mice (Figures S1F and S1G). Contrastingly, we did not detect a significant difference in decay time constant of the NMDAR current between group-housed and socially-isolated mice (Figure S1H). Finally, to confirm an increase in GluR2-lacking AMPARs at these synapses, we applied 1-naphthyl acetyl spermine (NASPM, a selective blocker of GluR2-lacking AMPARs) to brain slices prepared from group-housed or socially isolated mice. This reduced the amplitude of the evoked AMPAR current recorded in DRN DA neurons from socially isolated, but not group-housed, mice (Figure 1E). Taken together, this suggests that social isolation induces a relative increase in GluR2-lacking AMPARs at glutamatergic synapses onto DRN DA neurons (Figure 1F).

Activity of DRN DA Neurons In Vivo Increases upon Initial Social Contact after Social Isolation

We next considered whether acute social isolation affected the naturally occurring activity within DRN DA neurons. To address this question, we utilized a genetically encodable fluorescent calcium indicator, GCaMP6m (Chen et al., 2013), combined with fiber photometry to enable real-time recording of fluctuations in

Figure 1. 24 Hours of Social Isolation Induces Synaptic Potentiation onto DRN DA Neurons

(A) AMPAR/NMDAR ratios recorded from VTA DA neurons in mice socially isolated for 24 hr (n = 12) were not significantly different from group-housed mice (n = 8; unpaired t test: $t_{18} = 0.73$, $p = 0.47$).

(B) Low- (upper panel) and high-magnification (lower panels) confocal images of the DRN from a TH-GFP mouse showing GFP-expressing (green) and post hoc immunohistochemically verified TH-expressing (red) DA neurons with white arrows indicating co-labeled neurons.

(C) AMPAR/NMDAR ratios recorded from DRN DA neurons in mice socially isolated for 24 hr, either in a familiar cage or a novel cage (familiar isolated or novel isolated, respectively), were significantly greater than group-housed mice in familiar or novel cages (one-way ANOVA: $F_{3,47} = 5.910$, $^{**}p = 0.0017$; Newman-Keuls post hoc tests: $^{*}p < 0.05$, $^{**}p < 0.01$; n = 19 naive, 17 familiar isolated, 9 novel grouped, and 6 novel isolated). Scale bars, 20 pA, 20 ms.

(D) The AMPAR rectification index in DRN DA neurons was significantly greater in socially isolated mice, relative to naive mice (unpaired t test: $t_{21} = 2.417$, $^{*}p = 0.0248$, n = 9 naive, 14 isolated).

(E) Normalized AMPAR-mediated EPSC amplitude during bath application of NASPM, and representative averaged EPSCs from a naive and socially isolated mouse (inset shows % change in EPSC amplitude following NASPM, relative to baseline). NASPM significantly reduced EPSC amplitude in socially isolated mice (n = 7), relative to naive mice (n = 8; unpaired t test: $t_{13} = 2.853$, $^{*}p = 0.0136$). Scale bars, 10 pA, 10 ms.

(F) Proposed model of AMPARs at synapses onto DRN DA neurons in group-housed mice and following social isolation.

Data are represented as mean ± SEM. See also Figure S1.

neural activity (Cui et al., 2013; Gunaydin et al., 2014). We targeted expression of GCaMP6m to DRN DA neurons by injection of an adeno-associated viral vector (AAV_5) carrying GCaMP6m (AAV_5-CAG-FLEX-GCaMP6m) into the DRN of tyrosine hydroxylase (TH)::IRES-Cre mice, which facilitated GCaMP6m expression in a Cre-dependent manner. An optic fiber implanted over the DRN allowed simultaneous delivery of 473 nm excitation light and collection of GCaMP6m emission by means of a dichroic and a photodetector (Figures 2A, 2B, and S2A).

To assess the effect of a social target on DRN DA activity, mice were recorded in their home cage during the introduction of a novel juvenile mouse. We compared the fluorescence signal, in response to initial contact with the juvenile mouse, in mice that had either been previously group-housed or socially isolated for 24 hr. Strikingly, in socially isolated mice, we observed a significant increase in the fluorescence signal in response to first contact with the juvenile mouse, compared with group-housed mice (Figures 2C–2F; Movie S1). Furthermore, in isolated mice, the activity in response to initial social contact was significantly greater than in response to initial interaction with a novel object (Figures 2C–2F).

This suggests that, following social isolation, the presence of a social stimulus is associated with a significant increase in DRN DA activity in vivo. This is consistent with our finding that synaptic inputs onto DRN DA neurons are potentiated following social isolation.

DRN DA Neurons Release DA and Glutamate

We next sought to characterize the neurotransmitter content of DRN DA neurons, establish their sites of release, and validate parameters for subsequent causal experimentation. The TH+ DRN neurons have been confirmed as DAergic as they express aromatic L-amino decarboxylase (AADC), the enzyme that catalyzes conversion of L-3,4-dihydroxyphenylalanine, the product of TH, to DA (Lu et al., 2006) and the DA transporter (DAT) (Dougalis et al., 2012). Furthermore, DRN TH+ neurons lack dopamine-β-hydroxylase, which is necessary to convert DA to norepinephrine (Nagatsu et al., 1979), and do not express 5-hydroxytryptamine (5-HT) (Stratford and Wirtshafter, 1990).

Still, it remained to be demonstrated where these neurons synapse, which neurotransmitters they release, and whether their activation is sufficient to elicit detectable neurotransmitter release in vivo. To address these questions, we expressed Channelrhodopsin-2 fused to the enhanced yellow fluorescent protein (ChR2-eYFP) in the DRN in a Cre-dependent manner (Figures 3A and 3B). In TH::Cre mice we found that 77.7% of eYFP+ neurons co-labeled with TH using immunohistochemistry (Figures S2B, S2D, and S2F). This is consistent with a previous study (McDevitt et al., 2014) and is similar to overlap reported in other mouse lines used to selectively target DA neurons, including TH-GFP and Pitx3-GFP (Dougalis et al., 2012). For comparison, we also examined the DRN of DAT::IRES-Cre mice and found a similar proportion (79.8%) of eYFP+ neurons were colabeled with TH (Figure S2C, S2E, and S2G). As previously suggested (Hökfelt et al., 1976; Rogers, 1992), it is possible that some DA neurons within this region express low levels of TH, which may be below the detection threshold for immunohistochemistry and, thus, result in a relatively high proportion of seemingly eYFP+/TH− neurons. Importantly, eYFP expression did not overlap with 5-HT+ (serotonergic) neurons in either TH::Cre or DAT::Cre mice (Figures S2B–S2G).

In order to confirm optically induced firing in DRN DA neurons, we recorded from ChR2-expressing neurons using whole-cell patch-clamp electrophysiology in brain slices (Figure 3C). We delivered 473 nm light in a train of eight pulses of 5 ms pulse-width at 30 Hz every 5 s, a pattern used for VTA stimulation to elicit DA release and promote behavioral changes (Gunaydin et al., 2014; Tsai et al., 2009). In the DRN, ChR2-expressing neurons reliably followed these photostimulation parameters (Figure 3D).

Consistent with previous reports, eYFP expression in DRN DA somata resulted in terminal expression in several regions including the medial prefrontal cortex (mPFC), bed nucleus of the stria terminalis (BNST), lateral hypothalamus, central amygdala (CeA), entorhinal cortex, and basolateral amygdala (Hasue and Shammah-Lagnado, 2002; Meloni et al., 2006; Swanson, 1982; Yoshida et al., 1989). We observed particularly dense terminal expression within the dorsolateral BNST (dlBNST) (Figure 3E) and the lateral part of the CeA (Figure 3F) and, therefore, tested the effects of optical activation of DRN DA neurons on these regions.

To confirm DA release in anesthetized TH::Cre mice, with Cre-dependent expression of ChR2 in the DRN, we performed in vivo fast-scan cyclic voltammetry (FSCV) (Figures S3A–S3C). Optical stimulation of DRN DA neurons elicited DA release in both the dlBNST (Figure 3G) and the CeA (Figure 3H). The peak-evoked DA release was greater in the dlBNST than the CeA at 30 Hz and 50 Hz (Figures S3D–S3F), suggesting possible differences in the dynamics of DA release and reuptake in these two regions. In response to eight pulses of 30 Hz stimulation, delivered every 5 s, DA transients were consistently recorded in the dlBNST. However, in the CeA, transients were inconsistent and signals did not adequately resolve as DA, perhaps suggesting DA release just below the FSCV detection threshold (Figures 3G and 3H).

To determine whether DRN DA neurons co-release glutamate and/or GABA in downstream targets, we prepared brain slices containing the dlBNST (Figure 4A) or CeA (Figure 4B) from TH::Cre and DAT::Cre mice expressing ChR2 in a Cre-dependent manner in the DRN and recorded from neurons within the region of terminal expression (Figures S4A–S4D). Photostimulation of ChR2-expressing DA terminals elicited a short-latency fast AMPAR-mediated excitatory postsynaptic current (EPSC) in 25/30 neurons recorded in the dlBNST and 17/21 neurons in the CeA (Figure 4C-F). There was no significant difference in the proportion of neurons responding with an EPSC in TH::Cre and DAT::Cre mice, so these data were pooled (Figures S4E–S4G). Furthermore, the EPSCs persisted in the presence of tetradotoxin (TTX) and 4-aminopyridine (4AP), suggesting that they represent monosynaptic glutamate release from DRN DA terminals (Petreanu et al., 2007).

In contrast, optical stimulation of DAergic terminals did not elicit a short-latency $GABA_A$-mediated inhibitory postsynaptic current (IPSC) in the dlBNST or CeA (Figures 4C and 4D). However, in 3/23 dlBNST neurons and 4/24 CeA neurons we observed IPSC responses with a long and variable latency (Figures 4G and 4H), suggesting that terminal stimulation can activate GABAergic neurons to elicit a polysynaptic IPSC.

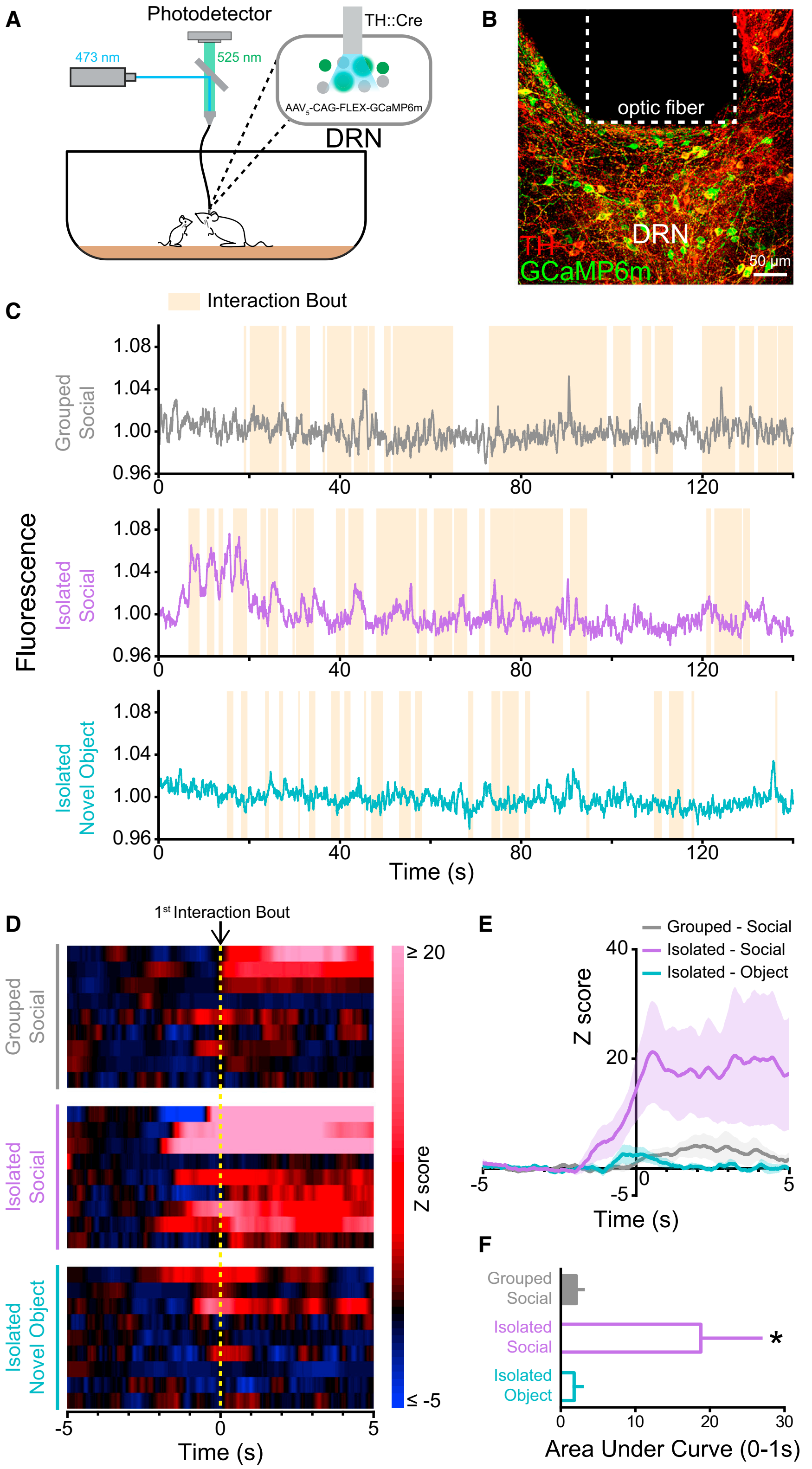

Figure 2. DRN DA Neurons Track Initial Social Contact Following Social Isolation

(A) Schematic for recording activity of GCaMP6m-expressing neurons.

(B) Image showing Cre-dependent expression of GCaMP6m in the DRN of a TH::Cre mouse, with optic fiber placement indicated.

(C) Representative traces of bulk fluorescence signal from DRN DA neurons, with shaded areas indicating interaction bouts. Mice were recorded under three conditions: group-housed mice presented with a juvenile mouse (gray), socially isolated mice presented with a juvenile mouse (lilac), or socially isolated mice presented with a novel object (teal).

(D) Heat maps showing the individual Z scores in response to the first interaction bout for each animal under each condition.

(E) Population Z score plots showing the averaged response to the first interaction bout.

(F) DRN DA neurons in socially isolated mice showed a significantly greater increase in activity upon first contact with the juvenile mouse, compared with group-housed mice or response to a novel object (n = 9; one-way ANOVA: $F_{2,16}$ = 4.978, *p = 0.0208; Bonferroni post hoc analysis: *p < 0.05 for both comparisons).

Data are represented as mean ± SEM. See also Figure S2 and Movie S1.

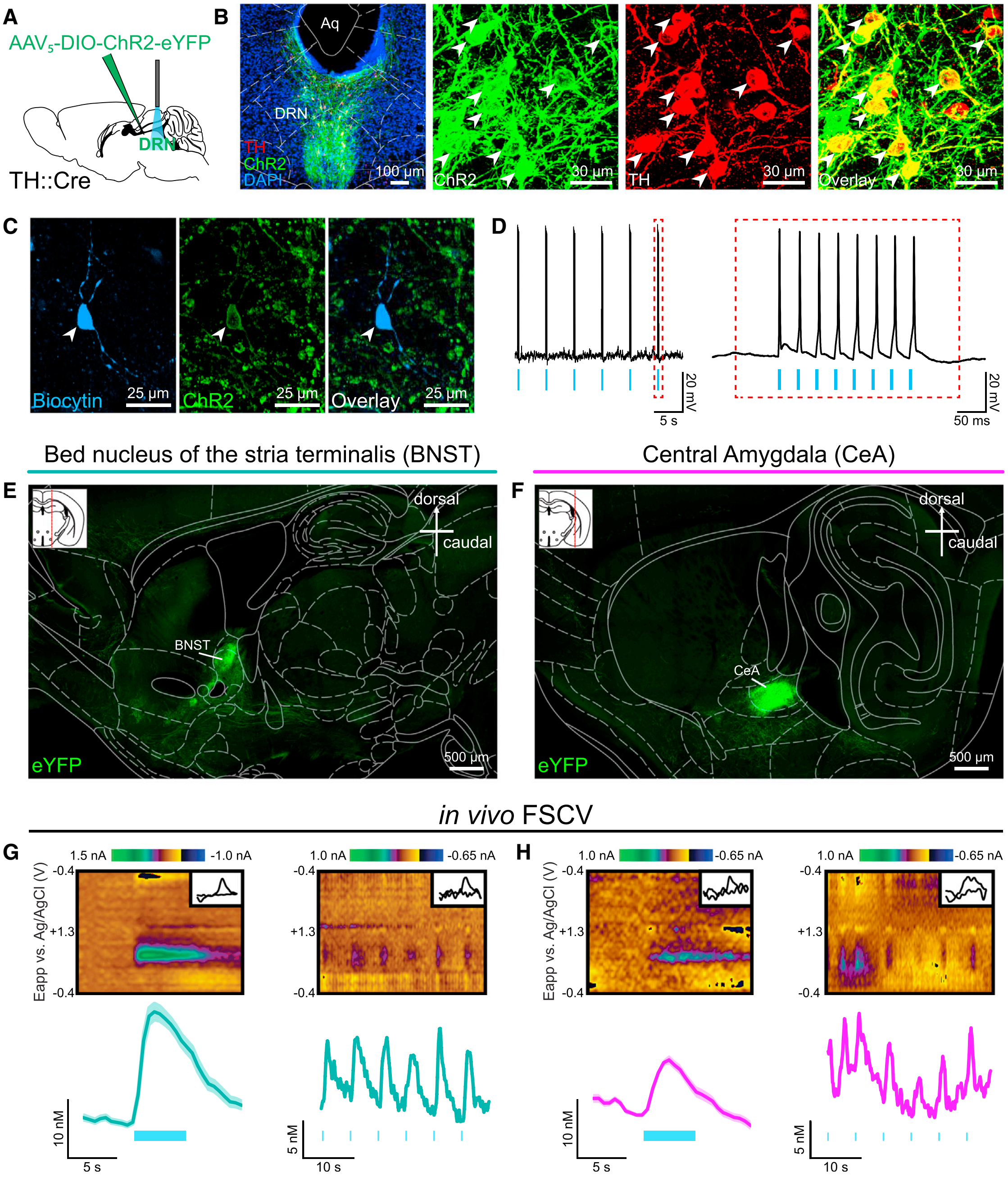

Figure 3. Photostimulation of DRN DA Neurons Elicits DA Release in the BNST and CeA

(A) AAV_5-DIO-ChR2-eYFP was injected into the DRN of TH::Cre mice to facilitate (B) ChR2 (green) expression in TH+ (red) DRN neurons. White arrows indicate selected co-labeled neurons.

(C) Example of a biocytin-filled, ChR2-expressing, DRN neuron recorded using ex vivo electrophysiology.

(D) ChR2 was activated using eight 5 ms pulses of blue light delivered every 5 s, which elicited a train of action potentials in the ChR2-expressing neuron.

(E) Sagittal brain sections showing dense terminal expression in the BNST and (F) CeA following AAV_5-DIO-eYFP injection into the DRN of a TH::Cre mouse.

(G) In vivo FSCV was performed in anesthetized TH::Cre mice following Cre-dependent expression of ChR2 in the DRN. Example color plots and average traces (± SEM) from the BNST (n = 5 mice; 7 recording sites) and (H) CeA (n = 4 mice; 5 recording sites) showing DA release evoked by 150 5 ms pulses of blue light delivered at 30 Hz (left panels) and a representative trace showing eight 5 ms pulses delivered every 5 s (right panels). Eapp, applied potential. Insets show cyclic voltammograms from representative color plots.

See also Figures S2 and S3.

We next confirmed the presence of a glutamatergic, but not GABAergic, marker in DRN DA neurons using two lines of transgenic mice: vesicular glutamate transporter 2 (VGLUT2)::IRES-Cre and vesicular GABA transporter (VGAT)::IRES-Cre. With Cre-dependent expression of eYFP in the DRN, a subset of TH+ neurons co-expressed VGLUT2 (Figures 4I and 4J), yet there was almost no overlap with VGAT (Figures 4K and 4L). This is consistent with the observed co-expression of VGLUT2 in more caudal and medially located DA neurons (Kawano et al., 2006).

Taken together, this demonstrates that optical stimulation of ChR2-expressing DRN DA neurons is sufficient to trigger rapid DA and glutamate release in two major downstream targets.

Optogenetic Activation of DRN DA Neurons Mimics a Loneliness-like State

To test for a causal relationship between DRN DA activation and an increase in sociability, we combined ChR2-mediated photostimulation with freely moving behavior (Figures S5A and S5B). We assessed social preference utilizing the three-chamber sociability task, in which time spent on the "social" side of the chamber (containing a juvenile mouse under a wire cup) is used as a measure of sociability (Moy et al., 2004; Silverman et al., 2010). Here, social approach is solely controlled by the experimental animal, as containment of the juvenile mouse removes the potential threat of territorial disputes, permitting an unadulterated measure of social interest (Silverman et al., 2010). We found that in TH::Cre mice expressing ChR2, but not eYFP, optical stimulation resulted in a significant increase in the proportion of time spent in the social zone (Figures 5A–5D), a result we replicated in DAT::Cre mice (Figures S5C and S5D).

This suggests that activation of DRN DA neurons plays a causal role in driving social behavior. Similarly, however, it has been shown that calcium signals can be detected in VTA DA neurons in response to a social target, and optical stimulation of these neurons promotes social interaction (Gunaydin et al., 2014), which has led to the hypothesis that VTA DA neurons represent a neural substrate for social reward. If DRN DA neurons were also encoding social reward, we would expect increased activity within these neurons to be positively reinforcing, similar to VTA activation, which has been shown to support ICSS (Witten et al., 2011) and conditioned place preference (CPP) (Tsai et al., 2009). Conversely, if DRN DA neurons are motivating social approach, due to an unmet need for social contact resulting from isolation, we would expect increased activity in these neurons (in the absence of a social stimulus) to elicit a negative affective state.

To distinguish between these two possibilities, we tested mice on an ICSS paradigm. However, we found that optical stimulation of DRN DA neurons did not support ICSS in TH::Cre (Figures 5E and 5F) or DAT::Cre mice (Figure S5E). Next, we assessed behavior in a real-time place avoidance (RTPA) assay, whereby mice freely explored a chamber in which one half was paired with blue light stimulation. Here, we observed avoidance of the light-paired side of the chamber relative to the unstimulated side in ChR2-, but not eYFP-expressing, TH::Cre and DAT::Cre mice (Figures S5F–S5H). Additionally, to eliminate confounding effects of stimulation-induced arousal, we examined behavior in a CPP paradigm (Figure 5G). During the test session, ChR2-expressing mice showed significant avoidance of the stimulation-associated zone, relative to eYFP-expressing mice (Figures 5H–5J). Furthermore, using additional behavioral assays, we found that optical stimulation of DRN DA neurons had no detectable effect on locomotion, novelty preference, or anxiety-related behavior (Figures S5I–S5X). This suggests that, in stark contrast to VTA DA neurons, optical activation of DRN DA neurons produces an aversive state.

In sum, we find that increasing activity of DRN DA neurons in group-housed mice promotes social preference but also elicits a negative affective state in the absence of a social target. We posit that this recapitulates a loneliness-like state, in which social approach is driven to alleviate the aversive state associated with social isolation.

Photoinhibition of DRN DA Neurons Reduces Isolation-Induced Sociability

Typically, in response to situations of social isolation or loneliness, individuals are motivated to re-establish social contact (Maner et al., 2007) and pay greater attention to social stimuli (Gardner et al., 2005; Pickett et al., 2004). In rodents, even an acute period of social isolation can elicit a rebound increase in social behavior (Niesink and van Ree, 1982; Panksepp and Beatty, 1980). In order to test whether DRN DA neurons are required to promote sociability following social isolation, we expressed a hyperpolarizing opsin (NpHR) in a Cre-dependent manner in the DRN of TH::Cre mice to mediate optical inhibition (Figure S6A).

Our in vivo recordings (Figure 2) revealed that the increase in DRN DA activity, on initial contact with a social target, was significantly greater in isolated, compared with group-housed, mice. First, therefore, we tested whether optical inhibition of DRN DA neurons altered social preference in group-housed mice using the three-chamber sociability task. Consistent with our recording results, neither NpHR- nor eYFP-expressing mice showed a significant difference in the proportion of time spent in the social zone with optical inhibition (Figure 6A and 6B). Furthermore, we did not observe significant effects of optical inhibition on behavioral measures of arousal or anxiety-related behavior (Figures S6B–S6J).

We next socially isolated NpHR- and eYFP-expressing mice for 24 hr and then tested social preference with optical inhibition. This revealed that mice expressing NpHR spent a significantly lower proportion of time in the social zone, compared with eYFP-expressing mice (Figure 6C). We then compared the social preference of mice tested with optical inhibition while group-housed and following social isolation. While eYFP-expressing mice showed the typical trend toward an increase in social preference following isolation, optical inhibition in NpHR-expressing mice resulted in a significant decrease in social preference following isolation (Figures 6D and 6E).

Collectively, these data suggest that inhibition of DRN DA neurons prevents the typical restoration of social contact following a period of isolation. This supports the hypothesis that DRN DA activity is required for motivating sociability in response to the negative state of isolation.

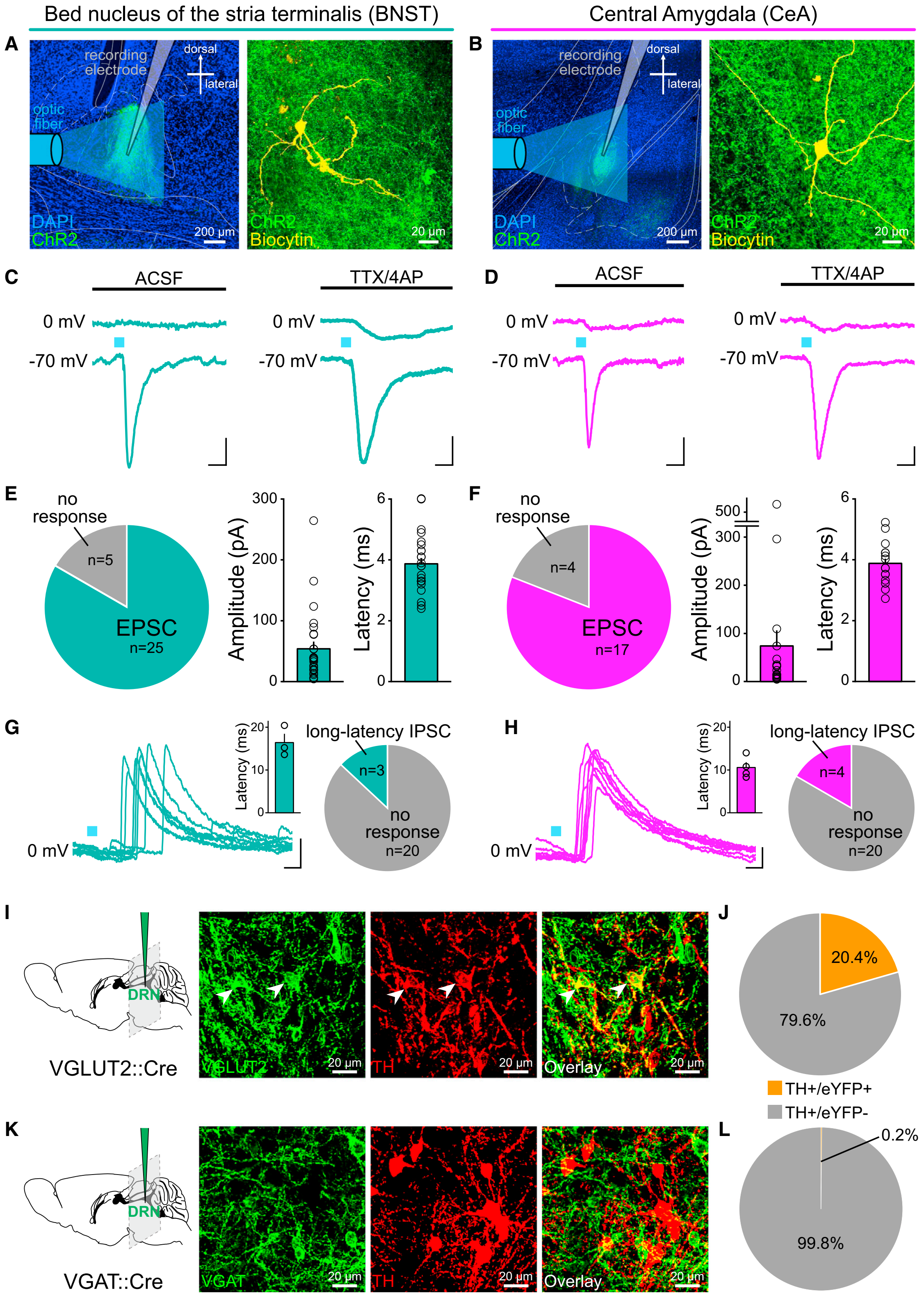

Figure 4. Optical Stimulation of DRN DA Neurons Elicits Monosynaptic Glutamate Release in the BNST and CeA

(A) Ex vivo electrophysiology was performed in the BNST and (B) CeA following Cre-dependent expression of ChR2 in the DRN of TH::Cre and DAT::Cre mice.

(legend continued on next page)

Prior Social Experience Predicts Functional Role of DRN DA Neurons

Social groups vary in terms of their size, complexity, and the nature of interactions between individuals. Given that the size of social networks has been correlated to structural differences in the brain (Sallet et al., 2011), we considered whether the degree of synaptic potentiation induced by social isolation would be related to the previous social group size. We compared data from mice that either remained group-housed or experienced social isolation and found that the magnitude of the AMPAR/NMDAR ratio was positively correlated with the number of previous cagemates in socially isolated, but not continually group-housed, mice (Figures 7A and 7B). This suggests that prior social environment contributes to subsequent isolation-induced synaptic strength.

Mice, like primates, form dominance hierarchies when housed together, which are thought to aid stability of social groups (Drews, 1993; Uhrich, 1938). Dominant behavior in males includes agonistic displays of behavior, priority access to food and resources, territorial urine marking, and winning situations of social conflict (Wang et al., 2014). As a result, the subjective experience of social interaction is likely to differ between members of a social group dependent on their social rank. Thus, we next hypothesized that social rank could influence the behavioral effects elicited by activation or inhibition of DRN DA neurons.

We estimated relative dominance within each cage (see Supplemental Experimental Procedures and Figures S7A and S7B) and examined the relationship between social rank and the change in social preference elicited by photoactivation of DRN DA neurons (Figure 5C). Intriguingly, we found that relative dominance and change in social preference were positively correlated, such that optical stimulation of DRN DA neurons appeared to be more effective at promoting social preference in dominant mice (Figures 7C, 7D, and S7C). Next, we assessed the relationship between social dominance and preference for the optical stimulation zone in ChR2-expressing mice tested in the RTPA assay (Figure S5G). Here, we observed a negative correlation between relative dominance and preference for the stimulation zone, such that more dominant mice displayed greater avoidance of the light-paired side of the chamber (Figures 7E, 7F, and S7D).

Finally, we examined the change in social preference of NpHR-expressing mice when receiving optical inhibition while group-housed compared to following isolation. Conversely, we observed a significant negative correlation (Figures 7G, 7H, S7E, and S7F), indicating that photoinhibition produced a greater reduction in social preference following isolation in more dominant mice.

Thus, in all cases, dominant mice showed a greater degree of behavioral modulation upon optogenetic manipulation of DRN DA neurons.

DISCUSSION

Satisfying the Profile of a Neural Substrate for a Loneliness-like State

The characteristics we have observed in DRN DA neurons bear remarkable similarities to the hypotheses generated from human psychology in describing the "need to belong" (Baumeister and Leary, 1995). First, it has been hypothesized that a "social monitoring system" exists which assimilates information on an individual's current and desired level of social acceptance (Gardner et al., 2005; Leary et al., 1995). Our finding that acute social isolation induces synaptic plasticity at synapses onto DRN DA neurons (Figure 1) suggests that these neurons either play a role in detecting or in reconciling the disparity between the current and desired social environment.

Second, in a state of loneliness, wherein an individual's basic need for social connection is unmet (Peplau, 1978), processing of socially relevant information should be prioritized (Baumeister and Leary, 1995). Indeed, in humans, socially excluded individuals display an enhanced memory for social events (Gardner et al., 2000), and more lonely individuals show increased attention toward social cues (Gardner et al., 2005; Pickett et al., 2004). Consistently, in socially isolated mice, we observed a significantly greater increase in DRN DA activity during initial contact with a social target (Figure 2), compared with group-housed mice.

Third, it has been postulated that the motivation for social re-connection should elicit "goal-orientated behavior" (Baumeister and Leary, 1995). With photoactivation, we revealed that activating DRN DA neurons promoted social preference in group-housed mice (Figure 5). However, in the absence of a social target, mice avoided photoactivation of DRN DA neurons, suggesting that stimulation is aversive. This suggests that activation of these neurons may be recapitulating a loneliness-like state, which is marked by a negative-affective state in which the drive to seek social contact is increased.

Fourth, the motivation for social re-connection was hypothesized to be "sensitive to satiation patterns" (Baumeister and Leary, 1995). In line with this, in group-housed mice, we observed limited changes in DRN activity related to initial juvenile contact (Figure 2), and photoinhibition of these neurons did not alter social behavior (Figure 6). Therefore, under "sated" group-housed conditions, this type of motivation may not be playing a major role. In contrast, following social isolation, photoinhibition caused a reduction in social preference (Figure 6). This indicates that the activity within these neurons may only be necessary in situations in which the motivation for social contact is high, such as that experienced after social isolation.

It has been hypothesized that the "need to belong" represents a powerful motivational drive, comparable to the basic need for

(C and D) Optical stimulation of ChR2-expressing terminals with a 5 ms blue light pulse elicited a short-latency, fast EPSC (measured in voltage-clamp at −70 mV), which persisted in the presence of TTX/4AP, but no detectable short-latency IPSC (measured at 0 mV). Scale bars, 10 pA, 10 ms.
(E) The proportion of recorded neurons in the BNST and (F) CeA that responded with an EPSC (in the absence of TTX/4AP) and the amplitude and latency of each.
(G and H) Individual traces showing the long-latency IPSCs elicited by successive 5 ms pulses of blue light, delivered every 20 s. Insets show average IPSC latency; pie charts show the proportion of cells which responded with an IPSC. Scale bars, 50 pA, 10 ms.
(I and J) Confocal images showing eYFP-expressing neurons (green) in the DRN with post hoc immunohistochemistry for TH (red), following Cre-dependent expression of eYFP, in VGLUT2::Cre and (K and L) VGAT::Cre mice. White arrows indicate selected co-labeled cells. A significantly greater proportion of TH+ neurons co-expressed VGLUT2 (n = 119/582 neurons) compared with VGAT (n = 1/577 neurons; Chi-square = 128.30, $p < 0.0001$).
Data are represented as mean ± SEM. See also Figure S4.

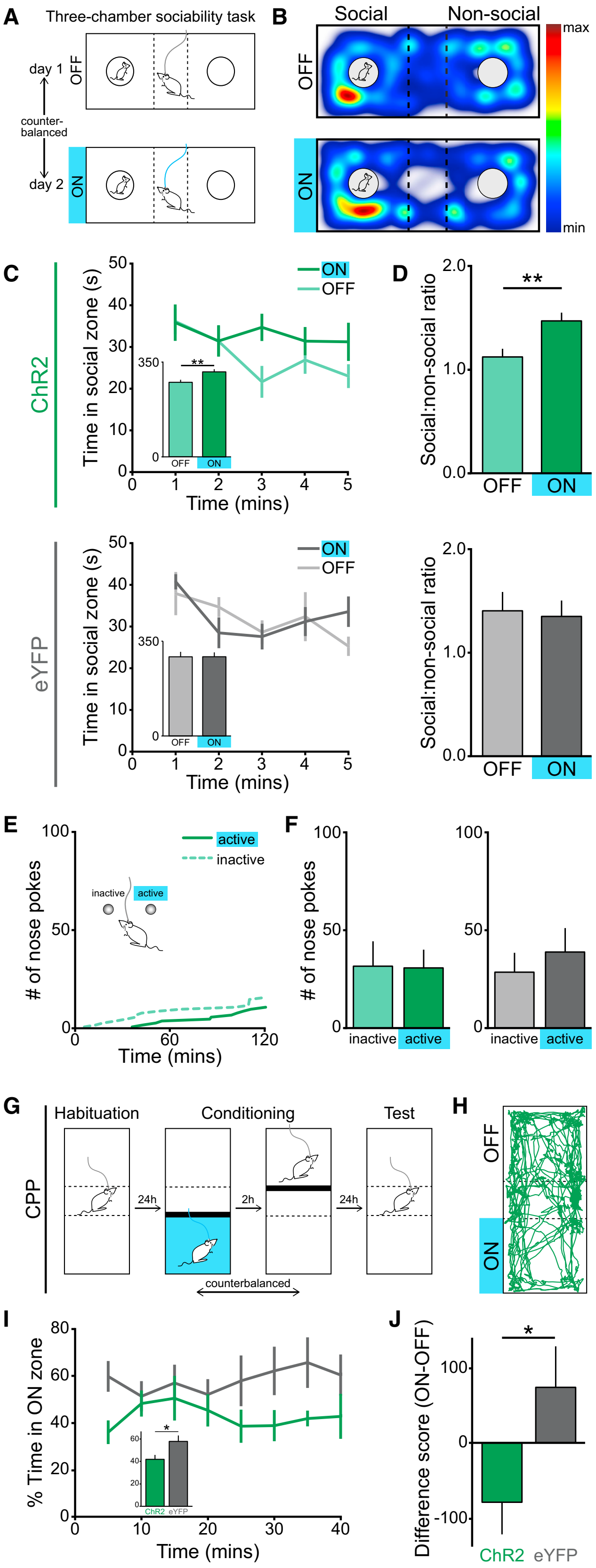

Figure 5. Optical Activation of DRN DA Neurons Elicits Social Preference and Place Avoidance

(A) TH::Cre mice were tested for social preference in the three-chamber sociability task.

(B) Representative spatial heat maps showing the location of a ChR2-expressing mouse.

(C) Time spent by ChR2- and eYFP-expressing mice in the social zone across the first 5 min of the task, with and without photostimulation (inset shows total time). ChR2-expressing, but not eYFP-expressing, mice showed a significant increase in the total time spent in the social zone, relative to the non-social zone, when receiving blue light stimulation (ChR2, inset, paired t test: t_{10} = 3.297, **p = 0.0081, n = 11; eYFP: t_{10} = 0.0100, p = 0.9922; n = 11) and (D) a significant increase in the social:non-social ratio (ChR2, paired t test: t_{10} = 3.843, **p = 0.0032, n = 11; eYFP: t_{10} = 0.1847, p = 0.8572, n = 11).

(E) Cumulative activity graph of nose pokes made by a ChR2-expressing mouse at the inactive and active (light-paired) ports during an ICSS task.

(F) Optical stimulation of DRN DA neurons did not support ICSS, as revealed by the number of nose pokes made into the inactive and active ports by ChR2- (active versus inactive, paired t test: t_4 = 0.0811, p = 0.9393, n = 5) and eYFP-expressing mice (active versus inactive, paired t test: t_6 = 0.732, p = 0.4917, n = 7).

(G) CPP paradigm and (H) representative track from a ChR2-expressing mouse during the first 10 min of the test session.

(I) Graph showing % time spent in the previously light-paired zone (inset shows first 10 min). ChR2-expressing mice showed significant avoidance of the previously light-paired zone, relative to eYFP mice, as shown by the % time spent in this zone (unpaired t test: t_{10} = 2.393, *p = 0.0378, n = 6 ChR2, 6 eYFP) and (J) the difference between the time spent in the previously light-paired and unpaired zones (unpaired t test: t_{10} = 2.241, *p = 0.0489, n = 6 ChR2, 6 eYFP). Data are represented as mean ± SEM. See also Figure S5.

food in a state of hunger (Baumeister and Leary, 1995). In considering this analogy with feeding behavior, it is intriguing to note that distinct neural circuits are thought to motivate food consumption related to the rewarding value of food (Nieh et al., 2015) and the need to obtain food to alleviate the negative state of hunger (Chen et al., 2015; Sternson et al., 2013). Thus, in a similar manner, social behavior may be driven by distinct neural circuits when motivated by social reward and when motivated by the punishment of social isolation.

The Subjective Experience of Social Isolation

In humans, a clear distinction can be made between an individual's subjective (perceived) isolation and their objective isolation. Perceived social isolation (or loneliness) reflects the quality of an individual's social interactions (Hawkley et al., 2008; Peplau, 1978) rather than their quantity or frequency. In humans, perceived social isolation predicts a poor outcome in numerous physical and mental health-related measures, entirely independent of the level of objective isolation (Adam et al., 2006; Cacioppo et al., 2006; Hawkley et al., 2006; Wen et al., 2006). While "loneliness" per se is difficult to directly test in mice, and consequently, the lack of animal literature on this phenomenon is noted (Cacioppo et al., 2014), social rank offers a useful estimate of an individual's subjective social experience.

When we overlaid this measure onto our behavioral data, remarkably, we observed a relationship in which dominant mice were more sensitive to the behavioral effects of manipulating DRN DA activity (Figure 7). It might be expected that the quality of social interaction for a dominant animal may be very different from a subordinate, and thus, their subjective experience of social isolation may also differ. Therefore, in their representation

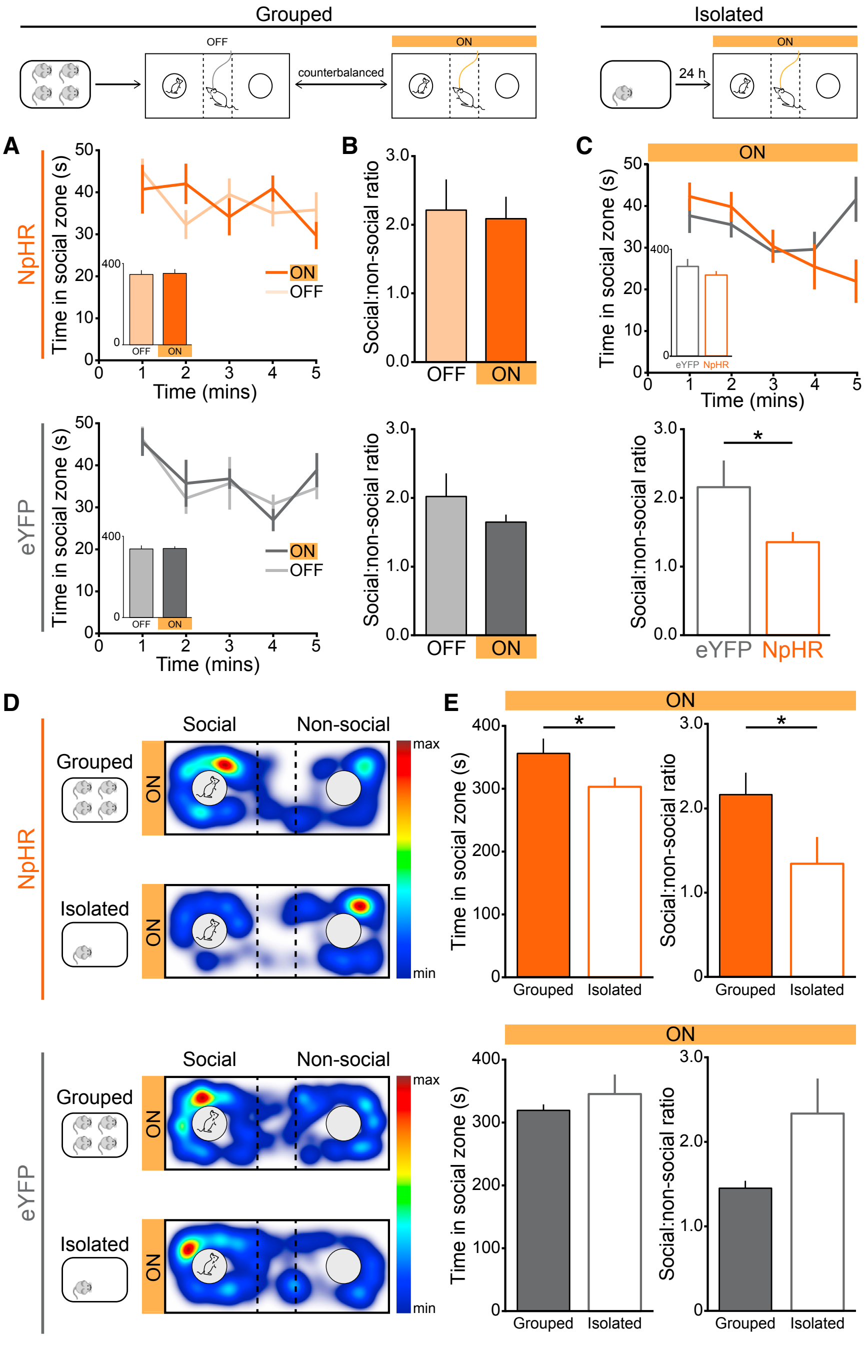

Figure 6. Optical Inhibition of DRN DA Neurons Reduces Social Preference Only Following Isolation

(A) Group-housed TH::Cre mice expressing NpHR (upper panels) or eYFP (lower panels) in the DRN, in a Cre-dependent manner, were assessed in the three-chamber sociability task. Time spent in the social zone across the first 5 min of the task (inset shows total time) and (B) the social:non-social ratio. Optical inhibition did not produce a detectable difference in the total time spent in the social zone (NpHR, inset, paired t test: $t_{12} = 0.1778$ p = 0.8619, n = 13; eYFP: $t_9 = 0.1788$, p = 0.8621, n = 10) or the social:non-social ratio (NpHR, paired t test: $t_{12} = 0.2414$, p = 0.8133, n = 13; eYFP: $t_9 = 1.293$, p = 0.2282, n = 10).

(C) Mice were isolated from their cagemates for 24 hr and then tested for sociability with optical inhibition. Time spent in the social zone by NpHR- and eYFP-expressing mice across the first 5 min (inset shows total time) and social:non-social ratio following isolation. NpHR-expressing mice spent a significantly lower proportion of time in the social zone after 24 hr of isolation compared with eYFP-expressing mice (social:non-social ratio, unpaired t test: $t_{16} = 2.236$, *p = 0.0400, n = 7 eYFP, 11 NpHR).

(D) Representative spatial heat maps showing the location of an NpHR- (upper panels) and eYFP-expressing mouse (lower panels) in the first 5 min of the task, when group-housed and following 24 hr of isolation.

(E) Time spent in the social zone and social:non-social ratio of mice tested with photoinhibition while group-housed and following social isolation. Photoinhibition in NpHR-expressing mice resulted in a significant reduction in time spent in the social zone (paired t test: $t_{10} = 2.740$, *p = 0.0208, n = 11) and social:non-social ratio (paired t test: $t_{10} = 2.239$, p = *0.0491, n = 11) following social isolation, compared to when group-housed.

Data are represented as mean ± SEM. See also Figure S6.

of a loneliness-like state, changes in DRN DA activity may only exert a significant effect on the behavior of individuals who are engaged in positively valued social interactions. Importantly, this also suggests that DRN DA neurons are not merely indicating the removal of sensory stimuli by social isolation but actually representing the subjective experience of a loneliness-like state.

It remains to be determined whether underlying neural differences play a causal role in dictating social rank and/or whether social rank itself imposes a change in neuronal properties. In monkeys, the attainment of a dominant social rank increases striatal $D_{2/3}$ receptor availability (Morgan et al., 2002), while in mice, altering synaptic efficacy in the mPFC is sufficient to promote a change in social rank (Wang et al., 2011). Furthermore, the observation that socially isolated and subordinate monkeys show similar $D_{2/3}$ receptor availability (Morgan et al., 2002) supports the notion that subordinate animals may be in a loneliness-like state, even while group-housed. Therefore, this may be one reason why manipulations of DRN DA activity were not as effective in promoting behavioral adaptations in subordinate animals.

Characterizing Components of the DRN DA Circuit

The relative bias of the DRN DA neurons in their projections to the BNST and CeA (Hasue and Shammah-Lagnado, 2002; Meloni et al., 2006) represents an important distinction from the VTA

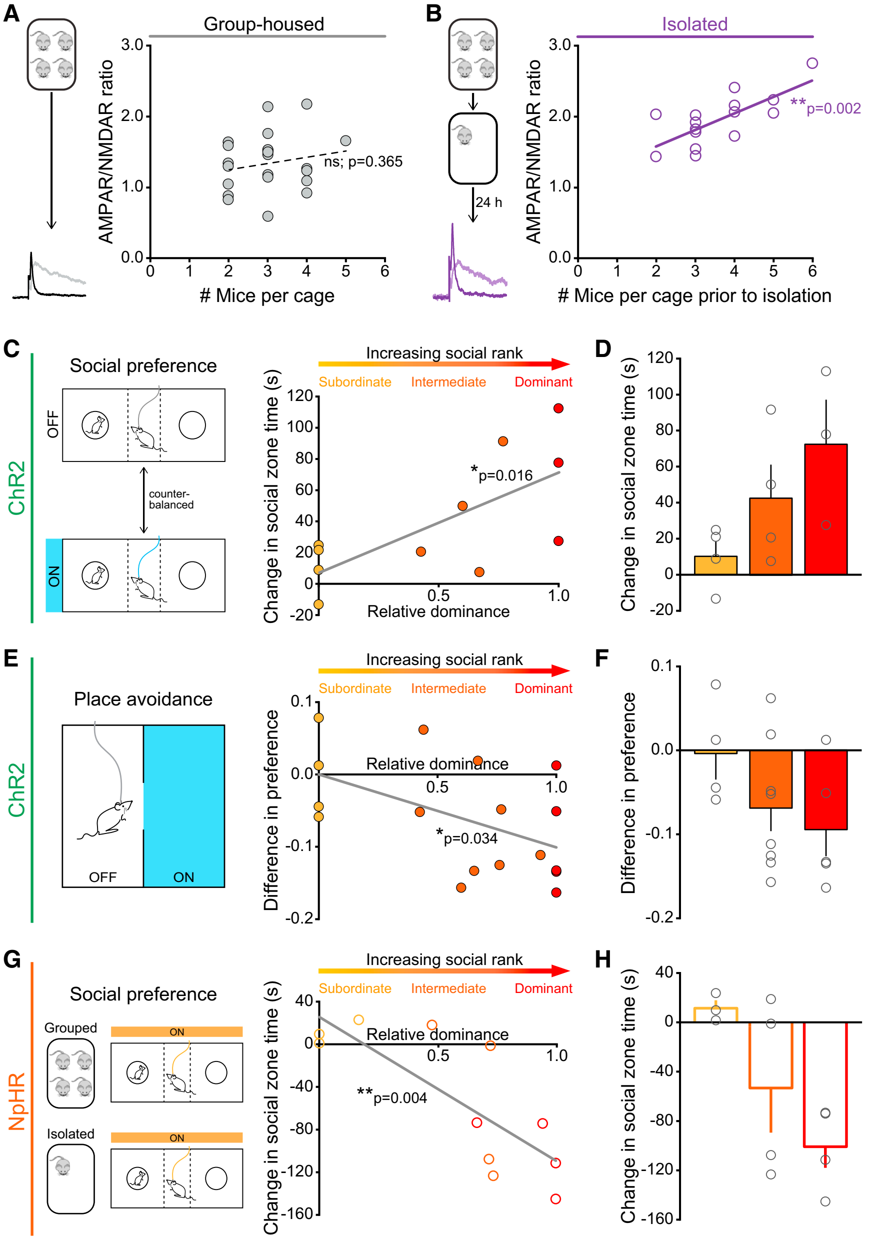

Figure 7. Prior Social Experience Predicts Magnitude of Social Isolation-Induced Synaptic Potentiation and Behavioral Response to Optogenetic Manipulation

(A and B) Scatter plot of AMPAR/NMDAR ratios recorded in DRN DA neurons plotted against the number of mice per cage in group-housed mice, and (B) the number of mice previously housed per cage in socially isolated mice. No significant correlation was detected in group-housed mice (Pearson's correlation: p = 0.3653, r^2 = 0.0374, n = 24), but a significant positive correlation was found in socially isolated mice (Pearson's correlation: **p = 0.0021, r^2 = 0.5282; n = 15).

(C) Relative social rank of TH::Cre mice was estimated with a score of 0 indicating the most subordinate and 1 the most dominant mouse in each cage. Relative dominance of ChR2-expressing mice plotted against the change in time spent in the social zone of the three-chamber apparatus (with blue light stimulation – without stimulation). There was a significant positive correlation between relative dominance and change in social zone time (Pearson's correlation: *p = 0.0163, r^2 = 0.4913; n = 11).

(D) Mean (+ SEM) change in social-zone time for mice of each social rank.

(E) Relative dominance of ChR2-expressing mice plotted against the difference in preference for the light-stimulation zone (proportion of time spent in the stimulation zone – proportion of time spent in the unstimulated zone) in the RTPA assay. There was a significant negative correlation between relative dominance and preference for the stimulation zone (Pearson's correlation: *p = 0.0338, r^2 = 0.2668, n = 17).

(F) Mean (– SEM) difference in preference for stimulation zone for mice of each social rank.

(G) Relative dominance of NpHR-expressing mice plotted against the change in social-zone time (isolated with yellow light – grouped with yellow light). There was a significant negative correlation between relative dominance and the change in social-zone time following isolation (Pearson's correlation: **p = 0.0038, r^2 = 0.6241, n = 11).

(H) Mean (± SEM) change in social zone time for mice of each social rank.

See also Figure S7.

population and suggests these DA neurons are part of a distinct circuit. The BNST and the CeA have been implicated in diverse behavioral functions (Davis et al., 2010; Janak and Tye, 2015; Kim et al., 2013), and DA receptor signaling in these regions modulates synaptic transmission and activity (Kash et al., 2008; de la Mora et al., 2010). Given that the BNST has been associated with mediating long-term "tonic" behavioral responses to sustained, diffuse, and/or unpredictable threats, whereas the CeA is thought to be more important in the rapid, acute response to threatening stimuli (Davis et al., 2010), it is likely that both of these regions may be important in mediating the observed effects of DRN DA stimulation on behavior.

Indeed, 24 hr of social isolation results in a blunting of long-term potentiation (LTP) in the BNST (Conrad et al., 2011). Given that DA release in the BNST has been shown to facilitate LTP (Kash et al., 2008), we speculate that increased DA release following social isolation may occlude LTP. However, we also demonstrate that glutamate can be released with optical stimulation of the DRN DA neurons, and the neuropeptide vasoactive intestinal peptide (VIP) is co-expressed in a subset of DA neurons (Dougalis et al., 2012). Thus, DA, glutamate, VIP, or the coordinated activity of these three neurotransmitters/neuromodulators may be important in facilitating the output of the DRN DA neurons.

Conclusion

Continued dissection of the neural mechanisms which govern social behavior is vital for the understanding and treatment of

social impairments, which characterize many debilitating neuropsychiatric disorders. Our data present an additional element for consideration in the control of social behavior, and support a novel role for a relatively unstudied population of DA neurons in representing the experience of social isolation.

EXPERIMENTAL PROCEDURES

Ex Vivo Electrophysiology

Brain slices (220 μm thick) containing the DRN or VTA were prepared from male heterozygous TH-GFP or Pitx3-GFP mice in order to target DA neurons. Whole-cell patch-clamp recordings were performed in voltage-clamp using a Multiclamp 700B amplifier (Molecular Devices) and Clampex 10.2 software (Molecular Devices). Afferent fibers were stimulated using a bipolar stimulating electrode (FHC) and glutamatergic currents were isolated by addition of picrotoxin (100 μM) to the ACSF.

Fiber Photometry

TH::Cre mice received an injection of AAV_5-CAG-FLEX-GCaMP6m into the DRN, and an optic fiber, held in a stainless steel ferrule, was implanted in the region. The photometry system was constructed similar to previously described (Gunaydin et al., 2014). GCaMP6m fluorescence was recorded while mice were in their home cage for 5 min before and after addition of a juvenile mouse or novel object.

Fast-Scan Cyclic Voltammetry (FSCV)

Anesthetized in vivo FSCV experiments were conducted similar to those previously described (Tsai et al., 2009). TH::Cre mice, which had received an injection of AAV_5-DIO-ChR2-eYFP into the DRN, were anesthetized with urethane and placed in a stereotaxic frame. Voltammetric recordings were collected, from either BNST or CeA, at 10 Hz by applying a triangular waveform (−0.4 V to +1.3 V to −0.4 V, 400 V/s) to a carbon-fiber electrode lowered into the region, versus an Ag/AgCl reference electrode.

Behavioral Testing

TH::Cre and DAT::Cre mice received an injection of AAV_5-DIO-ChR2-eYFP, AAV_5-DIO-NpHR3.0-eYFP, or AAV_5-DIO-eYFP into the DRN and were allowed at least 4 weeks for viral expression before behavioral testing. Mice were housed on a 12 hr:12 hr reverse light/dark cycle (lights off at 9:00 am) and tested during their active dark phase. Optical activation or inhibition was achieved by delivery of 473 nm light (30 Hz train of 8 pulses of 5 ms pulse width) or 593 nm light (constant), respectively, via an optic fiber secured in a stainless steel ferrule implanted over the DRN. For details of specific behavioral assays, see Supplemental Experimental Procedures.

SUPPLEMENTAL INFORMATION

Supplemental Information includes Supplemental Experimental Procedures, seven figures, and one movie and can be found with this article online at http://dx.doi.org/10.1016/j.cell.2015.12.040.

AUTHOR CONTRIBUTIONS

G.A.M., M.A.U., and K.M.T. designed the experiments and wrote the paper. G.A.M. performed ex vivo electrophysiology, E.H.N. conducted photometry recordings, and C.M.V.W. conducted FSCV recordings. G.A.M. and E.H.N. performed stereotaxic surgery. G.A.M., S.A.H., A.S.Y., R.V.P, and G.D.L. conducted behavioral experiments. G.A.M., C.M.V.W., R.E.T., G.D.L., A.S.Y., G.F.G., E.M.I., R.V.P., and C.P.W. performed immunohistochemistry and analyzed data.

ACKNOWLEDGMENTS

We would like to acknowledge M. Warden and C. Seo for advice regarding photometry, S.A. Hires and the entire Tye and Ungless Laboratories for helpful discussion, and A. Beyeler, R. Wichmann, P. Namburi, M. Bishop, and F. Brischoux for technical assistance. K.M.T. is a New York Stem Cell Foundation - Robertson Investigator and McKnight Scholar. This work was supported by funding from the JPB Foundation, PIIF, PNDRF, JFDP, Whitehall Foundation, Klingenstein Foundation, NARSAD Young Investigator Award, Alfred P Sloan Foundation, New York Stem Cell Foundation, McKnight Foundation, Whitehead Career Development Chair, R01-MH102441-01 (NIMH), RF1-AG047661-01 (NIA), and NIH Director's New Innovator Award DP2-DK-102256-01 (NIDDK). M.A.U. is supported by grant MC-A654-5QB70 from the U.K. Medical Research Council and a University Research Fellowship from The Royal Society. G.A.M. was supported by a Postdoctoral Fellowship from the Simons Center for the Social Brain at MIT. E.H.N. was supported by the NSF Graduate Research Program Fellowship, the Integrative Neuronal Systems Fellowship, and the Training Program in the Neurobiology of Learning and Memory. C.M.V.W. was supported by the Integrative Neuronal Systems Training Grant (NIGMS T32 GM007484) and an NSF Graduate Research Program Fellowship. A.S.Y. and R.V.P. were funded by the Undergraduate Research Opportunities Program at MIT. G.D.L. was supported by the MIT Summer Research Program and R.E.T. by the Northeastern Cooperative Education Program.

Received: March 20, 2015
Revised: September 28, 2015
Accepted: December 21, 2015
Published: February 11, 2016

REFERENCES

Adam, E.K., Hawkley, L.C., Kudielka, B.M., and Cacioppo, J.T. (2006). Day-to-day dynamics of experience–cortisol associations in a population-based sample of older adults. Proc. Natl. Acad. Sci. USA *103*, 17058–17063.

Baumeister, R.F., and Leary, M.R. (1995). The need to belong: desire for interpersonal attachments as a fundamental human motivation. Psychol. Bull. *117*, 497–529.

Bellone, C., and Lüscher, C. (2006). Cocaine triggered AMPA receptor redistribution is reversed in vivo by mGluR-dependent long-term depression. Nat. Neurosci. *9*, 636–641.

Bowie, D., and Mayer, M.L. (1995). Inward rectification of both AMPA and kainate subtype glutamate receptors generated by polyamine-mediated ion channel block. Neuron *15*, 453–462.

Brischoux, F., Chakraborty, S., Brierley, D.I., and Ungless, M.A. (2009). Phasic excitation of dopamine neurons in ventral VTA by noxious stimuli. Proc. Natl. Acad. Sci. USA *106*, 4894–4899.

Browne, A., Cambier, A., and Agha, S. (2011). Prisons Within Prisons: The Use of Segregation in the United States. Fed. Sentencing Report. *24*, 46–49.

Buss, D.M. (1990). The Evolution of Anxiety and Social Exclusion. J. Soc. Clin. Psychol. *9*, 196–201.

Cacioppo, J.T., Hughes, M.E., Waite, L.J., Hawkley, L.C., and Thisted, R.A. (2006). Loneliness as a specific risk factor for depressive symptoms: cross-sectional and longitudinal analyses. Psychol. Aging *21*, 140–151.

Cacioppo, S., Capitanio, J.P., and Cacioppo, J.T. (2014). Toward a neurology of loneliness. Psychol. Bull. *140*, 1464–1504.

Chen, T.-W., Wardill, T.J., Sun, Y., Pulver, S.R., Renninger, S.L., Baohan, A., Schreiter, E.R., Kerr, R.A., Orger, M.B., Jayaraman, V., et al. (2013). Ultrasensitive fluorescent proteins for imaging neuronal activity. Nature *499*, 295–300.

Chen, Y., Lin, Y.-C., Kuo, T.-W., and Knight, Z.A. (2015). Sensory detection of food rapidly modulates arcuate feeding circuits. Cell *160*, 829–841.

Conrad, K.L., Louderback, K.M., Gessner, C.P., and Winder, D.G. (2011). Stress-induced alterations in anxiety-like behavior and adaptations in plasticity in the bed nucleus of the stria terminalis. Physiol. Behav. *104*, 248–256.

Cui, G., Jun, S.B., Jin, X., Pham, M.D., Vogel, S.S., Lovinger, D.M., and Costa, R.M. (2013). Concurrent activation of striatal direct and indirect pathways during action initiation. Nature *494*, 238–242.

Davis, M., Walker, D.L., Miles, L., and Grillon, C. (2010). Phasic vs sustained fear in rats and humans: role of the extended amygdala in fear vs anxiety. Neuropsychopharmacology *35*, 105–135.

de la Mora, M.P., Gallegos-Cari, A., Arizmendi-García, Y., Marcellino, D., and Fuxe, K. (2010). Role of dopamine receptor mechanisms in the amygdaloid modulation of fear and anxiety: Structural and functional analysis. Prog. Neurobiol. *90*, 198–216.

Dölen, G., Darvishzadeh, A., Huang, K.W., and Malenka, R.C. (2013). Social reward requires coordinated activity of nucleus accumbens oxytocin and serotonin. Nature *501*, 179–184.

Dougalis, A.G., Matthews, G.A.C., Bishop, M.W., Brischoux, F., Kobayashi, K., and Ungless, M.A. (2012). Functional properties of dopamine neurons and co-expression of vasoactive intestinal polypeptide in the dorsal raphe nucleus and ventro-lateral periaqueductal grey. Eur. J. Neurosci. *36*, 3322–3332.

Drews, C. (1993). The Concept and Definition of Dominance in Animal Behaviour. Behaviour *125*, 283–313.

Eisenberger, N.I. (2012). The pain of social disconnection: examining the shared neural underpinnings of physical and social pain. Nat. Rev. Neurosci. *13*, 421–434.

Gardner, W.L., Pickett, C.L., and Brewer, M.B. (2000). Social Exclusion and Selective Memory: How the Need to belong Influences Memory for Social Events. Pers. Soc. Psychol. Bull. *26*, 486–496.

Gardner, W.L., Pickett, C.L., Jefferis, V., and Knowles, M. (2005). On the outside looking in: loneliness and social monitoring. Pers. Soc. Psychol. Bull. *31*, 1549–1560.

Gunaydin, L.A., Grosenick, L., Finkelstein, J.C., Kauvar, I.V., Fenno, L.E., Adhikari, A., Lammel, S., Mirzabekov, J.J., Airan, R.D., Zalocusky, K.A., et al. (2014). Natural neural projection dynamics underlying social behavior. Cell *157*, 1535–1551.

Hall, F.S., Wilkinson, L.S., Humby, T., Inglis, W., Kendall, D.A., Marsden, C.A., and Robbins, T.W. (1998). Isolation rearing in rats: pre- and postsynaptic changes in striatal dopaminergic systems. Pharmacol. Biochem. Behav. *59*, 859–872.

Hasue, R.H., and Shammah-Lagnado, S.J. (2002). Origin of the dopaminergic innervation of the central extended amygdala and accumbens shell: a combined retrograde tracing and immunohistochemical study in the rat. J. Comp. Neurol. *454*, 15–33.

Hawkley, L.C., Masi, C.M., Berry, J.D., and Cacioppo, J.T. (2006). Loneliness is a unique predictor of age-related differences in systolic blood pressure. Psychol. Aging *21*, 152–164.

Hawkley, L.C., Hughes, M.E., Waite, L.J., Masi, C.M., Thisted, R.A., and Cacioppo, J.T. (2008). From social structural factors to perceptions of relationship quality and loneliness: the Chicago health, aging, and social relations study. J. Gerontol. B Psychol. Sci. Soc. Sci. *63*, S375–S384.

Hökfelt, T., Johansson, O., Fuxe, K., Goldstein, M., and Park, D. (1976). Immunohistochemical studies on the localization and distribution of monoamine neuron systems in the rat brain. I. Tyrosine hydroxylase in the mes- and diencephalon. Med. Biol. *54*, 427–453.

Hollmann, M., Hartley, M., and Heinemann, S. (1991). Ca2+ permeability of KA-AMPA–gated glutamate receptor channels depends on subunit composition. Science *252*, 851–853.

Holt-Lunstad, J., Smith, T.B., and Layton, J.B. (2010). Social relationships and mortality risk: a meta-analytic review. PLoS Med. *7*, e1000316.

House, J.S., Landis, K.R., and Umberson, D. (1988). Social relationships and health. Science *241*, 540–545.

Janak, P.H., and Tye, K.M. (2015). From circuits to behaviour in the amygdala. Nature *517*, 284–292.

Kash, T.L., Nobis, W.P., Matthews, R.T., and Winder, D.G. (2008). Dopamine enhances fast excitatory synaptic transmission in the extended amygdala by a CRF-R1-dependent process. J. Neurosci. *28*, 13856–13865.

Kawano, M., Kawasaki, A., Sakata-Haga, H., Fukui, Y., Kawano, H., Nogami, H., and Hisano, S. (2006). Particular subpopulations of midbrain and hypothalamic dopamine neurons express vesicular glutamate transporter 2 in the rat brain. J. Comp. Neurol. *498*, 581–592.

Kim, S.-Y., Adhikari, A., Lee, S.Y., Marshel, J.H., Kim, C.K., Mallory, C.S., Lo, M., Pak, S., Mattis, J., Lim, B.K., et al. (2013). Diverging neural pathways assemble a behavioural state from separable features in anxiety. Nature *496*, 219–223.

Lammel, S., Ion, D.I., Roeper, J., and Malenka, R.C. (2011). Projection-specific modulation of dopamine neuron synapses by aversive and rewarding stimuli. Neuron *70*, 855–862.

Lammel, S., Lim, B.K., Ran, C., Huang, K.W., Betley, M.J., Tye, K.M., Deisseroth, K., and Malenka, R.C. (2012). Input-specific control of reward and aversion in the ventral tegmental area. Nature *491*, 212–217.

Leary, M.R., Tambor, E.S., Terdal, S.K., and Downs, D.L. (1995). Self-esteem as an interpersonal monitor: The sociometer hypothesis. J. Pers. Soc. Psychol. *68*, 518–530.

Liu, S.J., and Zukin, R.S. (2007). Ca2+-permeable AMPA receptors in synaptic plasticity and neuronal death. Trends Neurosci. *30*, 126–134.

Loo, P.L.P.V., de Groot, A.C., Zutphen, B.F.M.V., and Baumans, V. (2001). Do Male Mice Prefer or Avoid Each Other's Company? Influence of Hierarchy, Kinship, and Familiarity. J. Appl. Anim. Welf. Sci. *4*, 91–103.

Lu, J., Jhou, T.C., and Saper, C.B. (2006). Identification of wake-active dopaminergic neurons in the ventral periaqueductal gray matter. J. Neurosci. *26*, 193–202.

Maner, J.K., DeWall, C.N., Baumeister, R.F., and Schaller, M. (2007). Does social exclusion motivate interpersonal reconnection? Resolving the "porcupine problem". J. Pers. Soc. Psychol. *92*, 42–55.

McDevitt, R.A., Tiran-Cappello, A., Shen, H., Balderas, I., Britt, J.P., Marino, R.A.M., Chung, S.L., Richie, C.T., Harvey, B.K., and Bonci, A. (2014). Serotonergic versus nonserotonergic dorsal raphe projection neurons: differential participation in reward circuitry. Cell Rep. *8*, 1857–1869.

Meloni, E.G., Gerety, L.P., Knoll, A.T., Cohen, B.M., and Carlezon, W.A., Jr. (2006). Behavioral and anatomical interactions between dopamine and corticotropin-releasing factor in the rat. J. Neurosci. *26*, 3855–3863.

Morgan, D., Grant, K.A., Gage, H.D., Mach, R.H., Kaplan, J.R., Prioleau, O., Nader, S.H., Buchheimer, N., Ehrenkaufer, R.L., and Nader, M.A. (2002). Social dominance in monkeys: dopamine D2 receptors and cocaine self-administration. Nat. Neurosci. *5*, 169–174.

Moy, S.S., Nadler, J.J., Perez, A., Barbaro, R.P., Johns, J.M., Magnuson, T.R., Piven, J., and Crawley, J.N. (2004). Sociability and preference for social novelty in five inbred strains: an approach to assess autistic-like behavior in mice. Genes Brain Behav. *3*, 287–302.

Nagatsu, I., Inagaki, S., Kondo, Y., Karasawa, N., and Nagatsu, T. (1979). Immunofluorescent studies on the localization of tyrosine hydroxylase and dopamine-beta-hydroxylase in the mes-, di-, and telencephalon of the rat using unperfused fresh frozen sections. Acta Histochem. Cytochem. *12*, 20–37.

Nieh, E.H., Matthews, G.A., Allsop, S.A., Presbrey, K.N., Leppla, C.A., Wichmann, R., Neve, R., Wildes, C.P., and Tye, K.M. (2015). Decoding neural circuits that control compulsive sucrose seeking. Cell *160*, 528–541.

Niesink, R.J., and van Ree, J.M. (1982). Short-term isolation increases social interactions of male rats: a parametric analysis. Physiol. Behav. *29*, 819–825.

Panksepp, J., and Beatty, W.W. (1980). Social deprivation and play in rats. Behav. Neural Biol. *30*, 197–206.

Peplau, L.A. (1978). Loneliness: A bibliography of research and theory (American Psychological Association).

Petreanu, L., Huber, D., Sobczyk, A., and Svoboda, K. (2007). Channelrhodopsin-2-assisted circuit mapping of long-range callosal projections. Nat. Neurosci. *10*, 663–668.

Pickett, C.L., Gardner, W.L., and Knowles, M. (2004). Getting a cue: the need to belong and enhanced sensitivity to social cues. Pers. Soc. Psychol. Bull. *30*, 1095–1107.

Puglisi-Allegra, S., and Cabib, S. (1997). Psychopharmacology of dopamine: the contribution of comparative studies in inbred strains of mice. Prog. Neurobiol. *51*, 637–661.

Robinson, D.L., Heien, M.L.A.V., and Wightman, R.M. (2002). Frequency of dopamine concentration transients increases in dorsal and ventral striatum of male rats during introduction of conspecifics. J. Neurosci. *22*, 10477–10486.

Rogers, J.H. (1992). Immunohistochemical markers in rat brain: colocalization of calretinin and calbindin-D28k with tyrosine hydroxylase. Brain Res. *587*, 203–210.

Rozov, A., and Burnashev, N. (1999). Polyamine-dependent facilitation of postsynaptic AMPA receptors counteracts paired-pulse depression. Nature *401*, 594–598.

Saal, D., Dong, Y., Bonci, A., and Malenka, R.C. (2003). Drugs of abuse and stress trigger a common synaptic adaptation in dopamine neurons. Neuron *37*, 577–582.

Sallet, J., Mars, R.B., Noonan, M.P., Andersson, J.L., O'Reilly, J.X., Jbabdi, S., Croxson, P.L., Jenkinson, M., Miller, K.L., and Rushworth, M.F.S. (2011). Social network size affects neural circuits in macaques. Science *334*, 697–700.

Saper, C.B., and Petito, C.K. (1982). Correspondence of melanin-pigmented neurons in human brain with A1-A14 catecholamine cell groups. Brain *105*, 87–101.

Silverman, J.L., Yang, M., Lord, C., and Crawley, J.N. (2010). Behavioural phenotyping assays for mouse models of autism. Nat. Rev. Neurosci. *11*, 490–502.

Sternson, S.M., Nicholas Betley, J., and Cao, Z.F. (2013). Neural circuits and motivational processes for hunger. Curr. Opin. Neurobiol. *23*, 353–360.

Stratford, T.R., and Wirtshafter, D. (1990). Ascending dopaminergic projections from the dorsal raphe nucleus in the rat. Brain Res. *511*, 173–176.

Swanson, L.W. (1982). The projections of the ventral tegmental area and adjacent regions: a combined fluorescent retrograde tracer and immunofluorescence study in the rat. Brain Res. Bull. *9*, 321–353.

Swanson, G.T., Kamboj, S.K., and Cull-Candy, S.G. (1997). Single-channel properties of recombinant AMPA receptors depend on RNA editing, splice variation, and subunit composition. J. Neurosci. *17*, 58–69.

Tsai, H.-C., Zhang, F., Adamantidis, A., Stuber, G.D., Bonci, A., de Lecea, L., and Deisseroth, K. (2009). Phasic firing in dopaminergic neurons is sufficient for behavioral conditioning. Science *324*, 1080–1084.

Uhrich, J. (1938). The social hierarchy in albino mice. J. Comp. Psychol. *25*, 373–413.

Ungless, M.A., Whistler, J.L., Malenka, R.C., and Bonci, A. (2001). Single cocaine exposure in vivo induces long-term potentiation in dopamine neurons. Nature *411*, 583–587.

Walker, J., Illingworth, C., Canning, A., Garner, E., Woolley, J., Taylor, P., and Amos, T. (2014). Changes in mental state associated with prison environments: a systematic review. Acta Psychiatr. Scand. *129*, 427–436.

Wang, F., Zhu, J., Zhu, H., Zhang, Q., Lin, Z., and Hu, H. (2011). Bidirectional control of social hierarchy by synaptic efficacy in medial prefrontal cortex. Science *334*, 693–697.

Wang, F., Kessels, H.W., and Hu, H. (2014). The mouse that roared: neural mechanisms of social hierarchy. Trends Neurosci. *37*, 674–682.

Wen, M., Hawkley, L.C., and Cacioppo, J.T. (2006). Objective and perceived neighborhood environment, individual SES and psychosocial factors, and self-rated health: an analysis of older adults in Cook County, Illinois. Soc. Sci. Med. *63*, 2575–2590.

Williams, K.D., and Sommer, K.L. (1997). Social Ostracism by Coworkers: Does Rejection Lead to Loafing or Compensation? Pers. Soc. Psychol. Bull. *23*, 693–706.

Witten, I.B., Steinberg, E.E., Lee, S.Y., Davidson, T.J., Zalocusky, K.A., Brodsky, M., Yizhar, O., Cho, S.L., Gong, S., Ramakrishnan, C., et al. (2011). Recombinase-driver rat lines: tools, techniques, and optogenetic application to dopamine-mediated reinforcement. Neuron *72*, 721–733.

Yoshida, M., Shirouzu, M., Tanaka, M., Semba, K., and Fibiger, H.C. (1989). Dopaminergic neurons in the nucleus raphe dorsalis innervate the prefrontal cortex in the rat: a combined retrograde tracing and immunohistochemical study using anti-dopamine serum. Brain Res. *496*, 373–376.

Cell

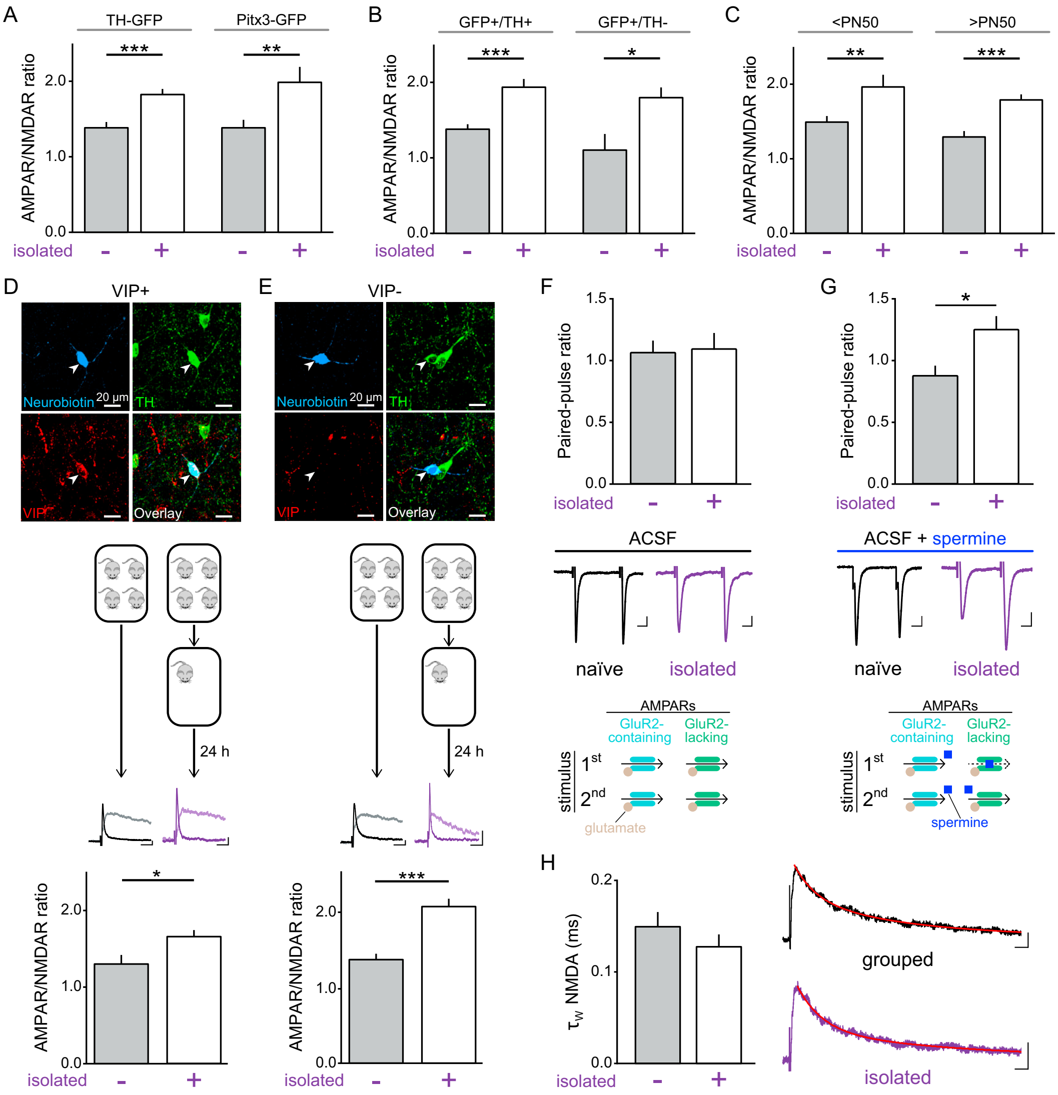

Figure S1. Electrophysiological Measures of AMPAR and NMDAR Current Properties in DRN DA Neurons of Group-Housed and Socially Isolated Mice, Related to Figure 1

(A) AMPAR/NMDAR ratios recorded in DRN DA neurons from both TH-GFP and Pitx3-GFP mice showed a significant increase following 24 hr social isolation in comparison to group-housed mice (TH-GFP – unpaired t test: $t_{48} = 4.100$, ***p = 0.0002; n = 25 grouped, 25 isolated; Pitx3-GFP – unpaired t test: $t_{27} = 2.813$, **p = 0.0090; n = 17 grouped, 12 isolated).

(B) AMPAR/NMDAR ratios recorded from GFP+/TH+ and GFP+/TH- DRN neurons, in both transgenic mouse lines, were significantly greater in socially isolated compared with group-housed mice (GFP+/TH+: unpaired t test: $t_{46} = 4.149$, ***p = 0.0001; n = 23 grouped, 25 isolated; GFP+/TH-: unpaired t test: $t_8 = 2.695$, *p = 0.0273; n = 5 grouped, 5 isolated).

(C) Both adolescent (< PN50) and adult (> PN50) mice showed a significant increase in the AMPAR/NMDAR ratio following social isolation (< PN50 – unpaired t test: $t_{40} = 3.011$, **p = 0.0045; n = 23 grouped, 19 isolated; ≥ PN50 – unpaired t test: $t_{33} = 4.226$, ***p = 0.0002; n = 18 grouped, 17 isolated).

(D and E) Example high-magnification confocal images of neurobiotin-filled (blue) neurons immunohistochemically identified as TH+ (green) and (D) VIP+ (red) or (E) VIP−. Illustration of mouse housing manipulations, example AMPAR/NMDAR ratios recorded in DRN DA neurons, and bar charts showing the mean ratio for each condition. Both VIP+ and VIP− DRN DA neurons showed a significantly greater AMPAR/NMDAR ratio following social isolation (VIP+: unpaired t test: $t_{18} = 2.210$, *p = 0.0403; n = 10 grouped, 10 isolated; VIP−: unpaired t test: $t_{36} = 4.78$, ***p < 0.0001; n = 18 grouped, 20 isolated). Scale bars, 20 pA, 20 ms.

(F) In ACSF, the AMPAR PPR was not significantly different between naive (n = 14) and socially isolated mice (n = 11; unpaired t test: $t_{23} = 0.0441$, p = 0.952).

(G) In the presence of the polyamine spermine, the AMPAR PPR was significantly greater in socially isolated relative to naive mice (unpaired t test: $t_{31} = 2.269$, *p = 0.0304, n = 11 naive, 22 isolated). Scale bars, 10 pA, 10 ms.

(H) The decay phase of the NMDAR current was fitted with a double exponential function to calculate the weighted decay time constant (τ_W). No significant difference was found in the decay time constant of the NMDAR current between group-housed and socially isolated mice (Mann-Whitney U = 248, p = 0.7002, n = 28 grouped, 19 isolated). Scale bars, 20 pA, 20 ms.

Data are represented as mean + SEM.

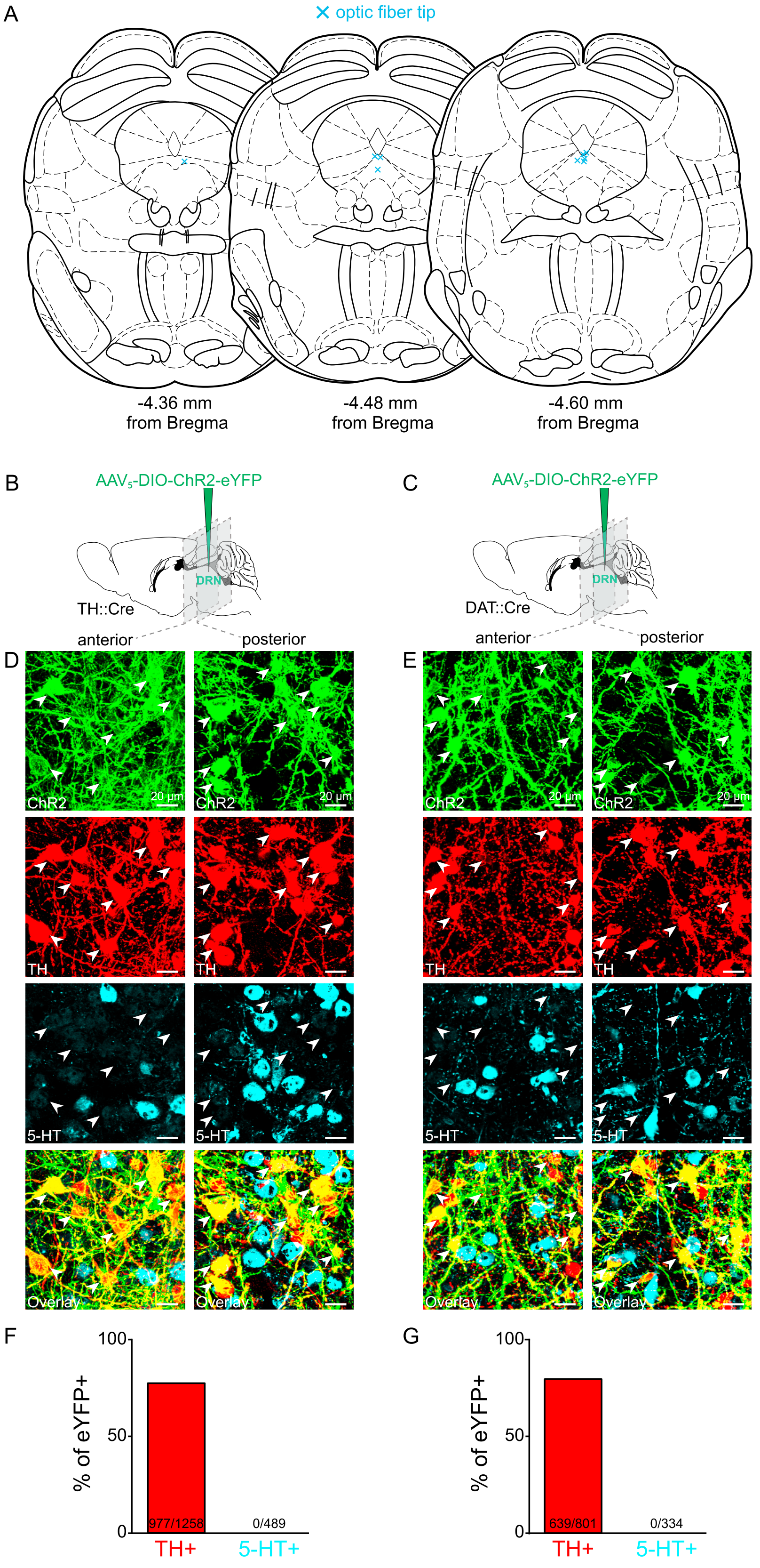

(legend on next page)

Figure S2. Placement of Optic Fiber in the DRN for Photometry Recordings and Immunohistochemical Characterization of ChR2-eYFP Expression in TH::Cre and DAT::Cre Mice, Related to Figures 2 and 3

(A) The position of the tip of the optic fiber (blue cross) in the DRN of TH::Cre mice expressing GCaMP6m used for photometry recordings.

(B) TH::Cre and (C) DAT::Cre mice received an injection of AAV_5-DIO-ChR2-eYFP into the DRN and coronal brain sections were prepared after at least 4 weeks of expression.

(D) High-magnification confocal images of eYFP+ neurons, within one anterior and one posterior section of the DRN, showing co-expression of eYFP with TH (red) but not 5-HT (cyan) in a TH::Cre and (E) a DAT::Cre mouse. White arrows indicate selected eYFP+/TH+ neurons.

(F) The proportion of eYFP+ neurons in the DRN co-labeled with TH and 5-HT in TH::Cre and (G) DAT::Cre mice. There was no significant difference in the proportion of eYFP+ neurons co-labeled with TH in TH::Cre and DAT::Cre mice (Chi-square = 1.2931, $p = 0.2555$, $n = 977/1258$ neurons for TH::Cre, $n = 639/801$ neurons for DAT::Cre).

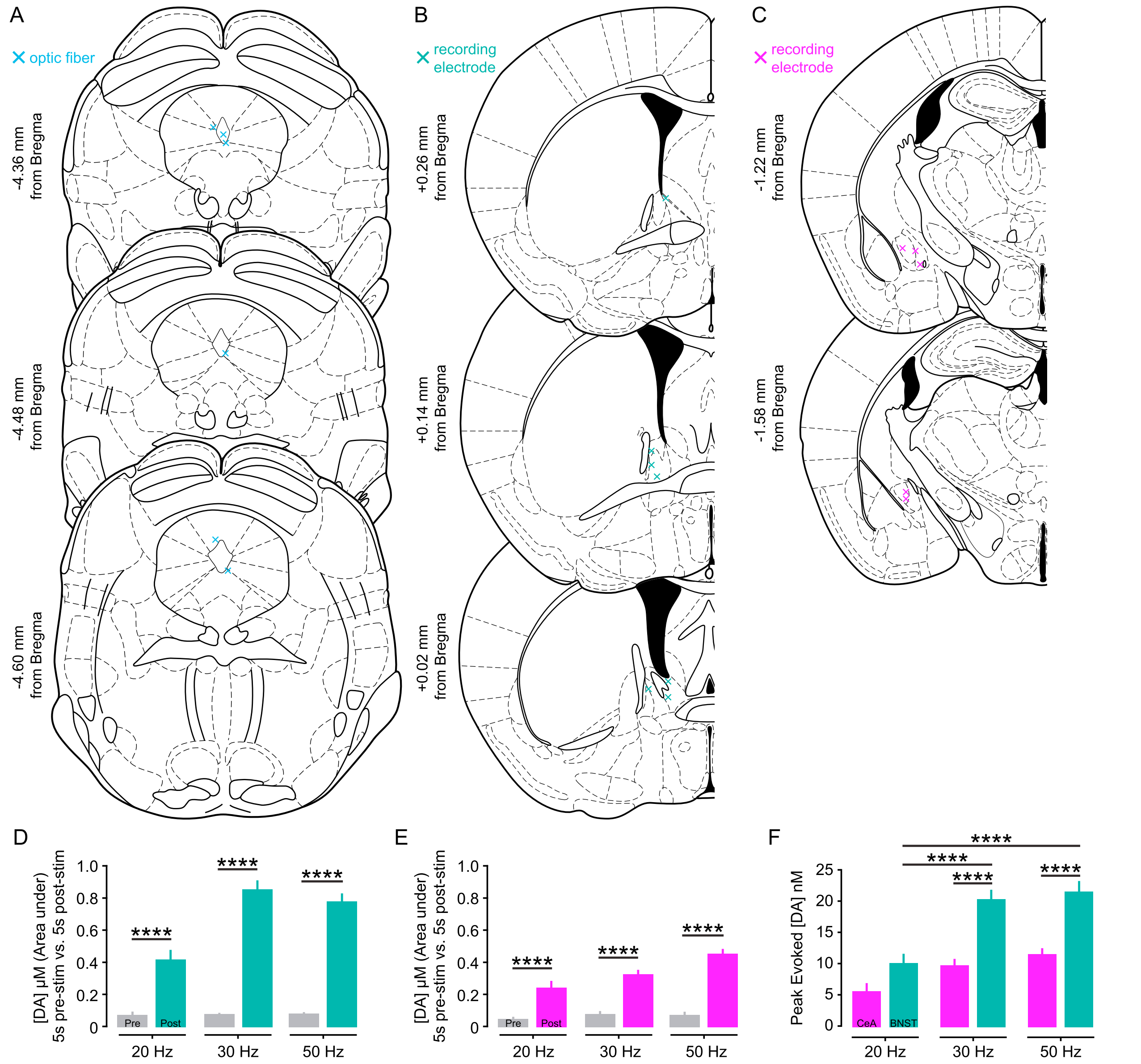

Figure S3. Placement of Optic Fiber Tip and Recording Sites for In Vivo FSCV and Evoked DA Release at Different Stimulation Frequencies, Related to Figure 3

(A) The position of the optic fiber tip (blue cross) in the DRN and (B) the recording electrode in the BNST (teal cross) and (C) CeA (magenta cross) of TH::Cre mice used for in vivo FSCV recordings.

(D–E) Extracellular DA concentration ([DA]), evoked by 150 pulses of blue light (5 ms duration) at 20, 30, and 50 Hz, was quantified by calculating the area under each trace during the time window 5 s prior to stimulation onset compared to the time window 5 s post-stimulation onset. (D) Optical stimulation of DRN DA neurons evoked a significant increase in [DA] in the BNST compared to pre-stimulation levels at 20 Hz (paired t test: $t_{44} = 5.761$, ****$p < 0.0001$), 30 Hz (paired t test: $t_{106} = 13.74$, ****$p < 0.0001$), and 50 Hz (paired t test; $t_{105} = 13.57$, ****$p < 0.0001$). (E) Optical stimulation of DRN DA neurons evoked a significant increase in [DA] in the CeA compared to pre-stimulation levels at 20 Hz (paired t test: $t_{51} = 4.914$, ****$p < 0.0001$), 30 Hz (paired t test: $t_{70} = 9.911$, ****$p < 0.0001$), and 50 Hz (paired t test: $t_{65} = 10.64$, ****$p < 0.0001$).

(F) As a group, peak [DA] was increased in a frequency-dependent manner (two-way ANOVA: $F_{2,441} = 22.19$, ****$p < 0.0001$). In the BNST, peak [DA] evoked by 30 Hz and 50 Hz stimulation was greater than that evoked by 20 Hz (Bonferroni multiple comparisons: 20 Hz versus 30 Hz, ****$p < 0.0001$; 20 Hz versus 50 Hz, ****$p < 0.0001$; 30 Hz versus 50 Hz, $p > 0.9999$). In contrast, peak [DA] in the CeA did not differ significantly for different stimulation frequencies (Bonferroni multiple comparisons: 20 Hz versus 30 Hz, $p = 0.7134$; 20 Hz versus 50 Hz, $p = 0.0806$; 30 Hz versus 50 Hz, $p > 0.9999$). Peak-evoked [DA] was greater in the BNST compared to the CeA at 30 Hz ($F_{1,441} = 54.39$, ****$p < 0.0001$; Bonferroni multiple comparisons: ****$p < 0.0001$) and 50 Hz (Bonferroni multiple comparisons: ****$p < 0.0001$).

Data are represented as mean + SEM.

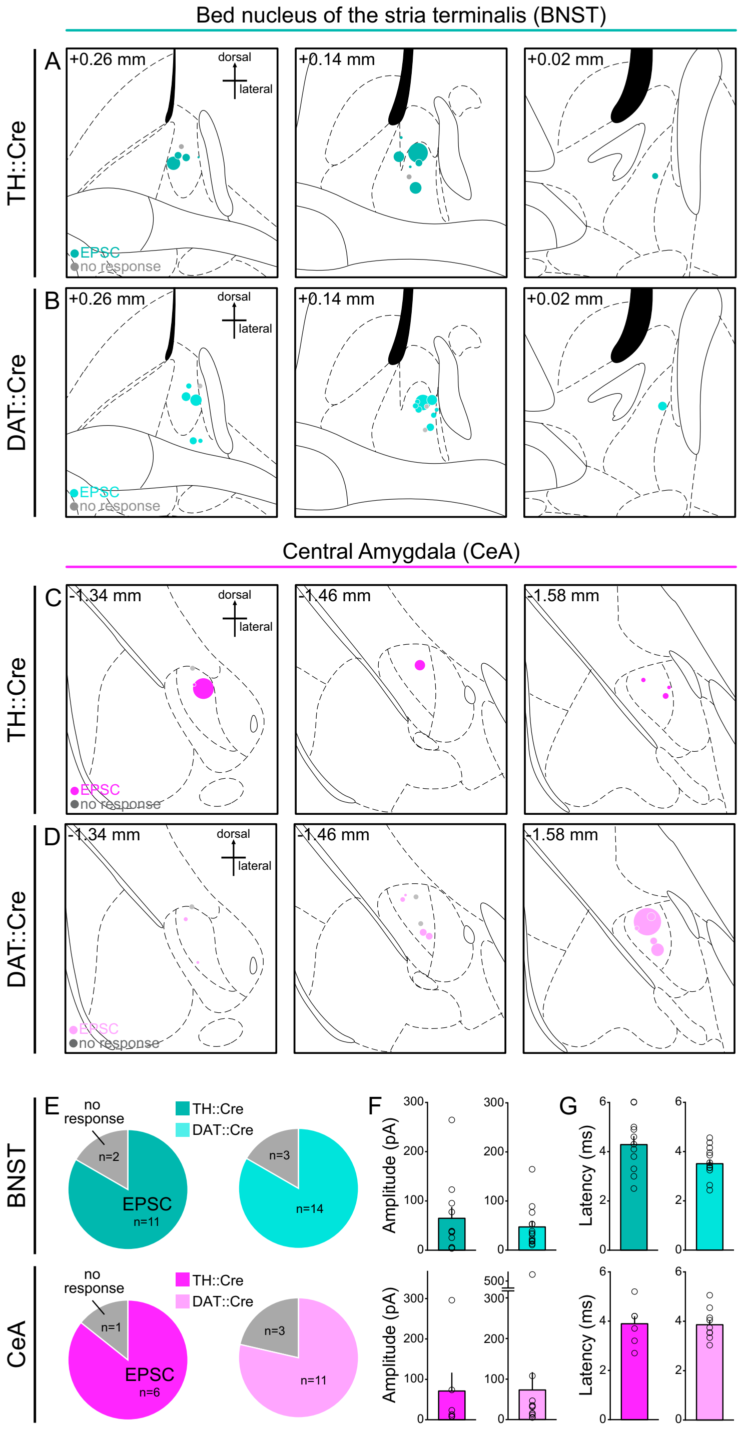

Figure S4. Optically Evoked Glutamate Currents in the BNST and CeA Following DRN DA Terminal Stimulation in TH::Cre and DAT::Cre Mice, Related to Figure 4

(A and B) Location of ex vivo recorded cells in the BNST and (C and D) CeA of TH::Cre and DAT::Cre mice, superimposed on coronal brain atlas sections (distance from Bregma shown in top left corner). The relative amplitude of the optically evoked EPSC from each cell is indicated by the size of the teal (BNST) or magenta (CeA) circle. Cells with no response are displayed in gray.

(E) Pie charts showing the proportion of cells that responded with an EPSC to a 5 ms blue light pulse in the BNST and CeA of TH::Cre and DAT::Cre mice and (F) the amplitude and (G) latency of each EPSC. There was no significant difference in the proportion of neurons which responded to optical stimulation with an EPSC in TH::Cre mice compared to DAT::Cre mice in the BNST (Chi-square = 0.0271, p = 0.8691) or CeA (Chi-square = 0.1544, p = 0.6944).

Data are represented as mean + SEM

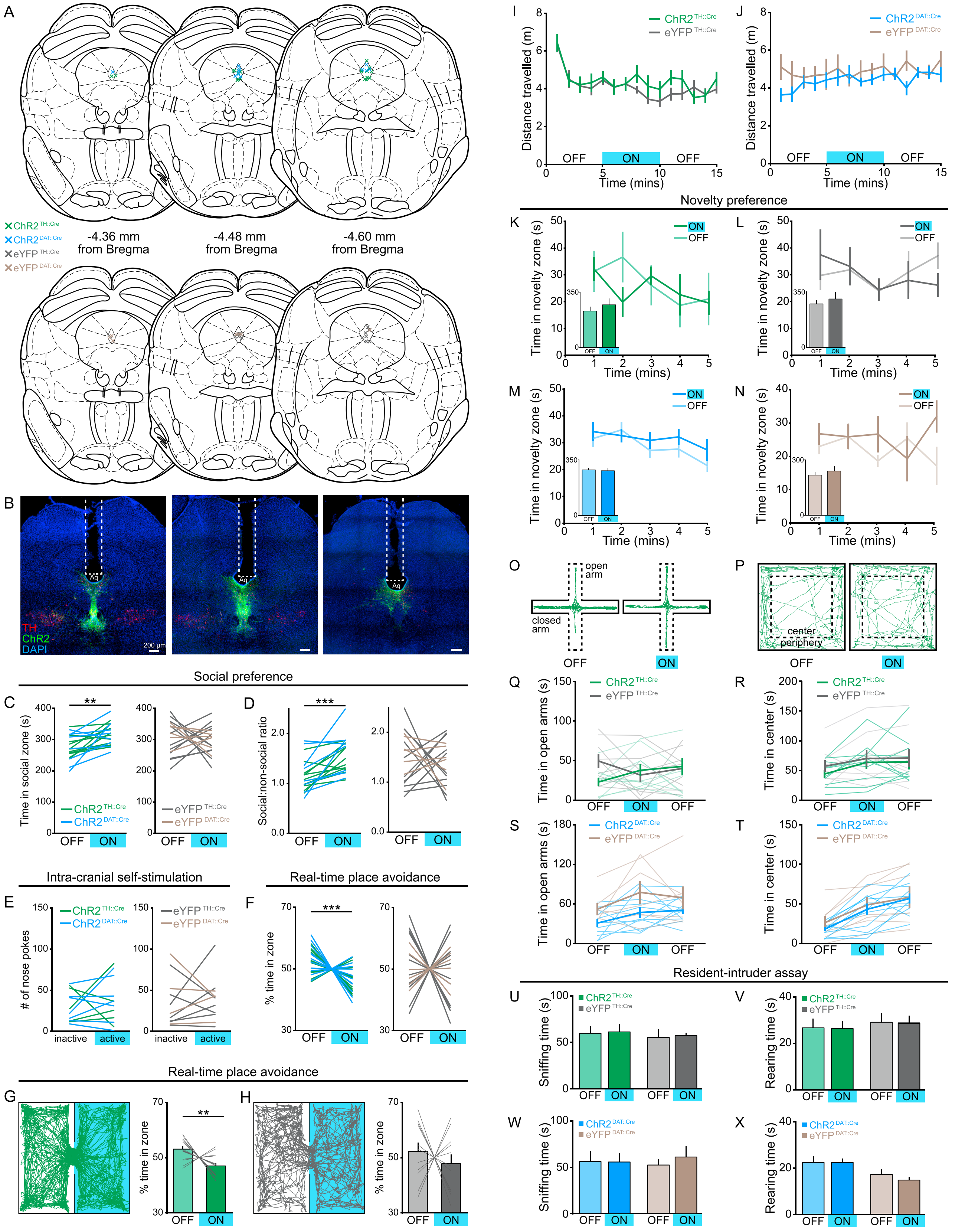

(legend on next page)

Figure S5. Effect of Photoactivation in TH::Cre and DAT::Cre Mice on Behavioral Measures of Social Preference, Avoidance, Arousal, and Anxiety-Related Behavior, Related to Figures 5 and 7

(A) The position of the optic fiber tip above the DRN of TH::Cre and DAT::Cre mice expressing ChR2 or eYFP used for behavioral analysis.

(B) Example confocal images showing the optic fiber track (white dashed line), ChR2-eYFP expression (green), and post hoc immunohistochemistry for TH (red) in the DRN of TH::Cre mice at each anteroposterior position indicated in A.

(C) Time spent in the social zone of the three-chamber apparatus and (D) social:non-social ratio of individual TH::Cre and DAT::Cre mice expressing ChR2 (green and blue, respectively) or eYFP (gray and brown, respectively) in DRN DA neurons. There was no significant difference in the behavior of TH::Cre and DAT::Cre mice with blue light stimulation (ChR2: time in social zone – unpaired t test: $t_{17} = 0.0565$, p = 0.9556; social:non-social ratio – $t_{17} = 1.185$, p = 0.2523, n = 11 TH::Cre, 8 DAT::Cre; eYFP: time in social zone – unpaired t test: $t_{15} = 0.0165$, p = 0.9870; social:non-social ratio – $t_{15} = 0.3015$, p = 0.7672, n = 11 TH::Cre, 6 DAT::Cre) or without stimulation (ChR2: time in social zone – unpaired t test: $t_{17} = 0.1162$, p = 0.9089; social:non-social ratio – $t_{17} = 0.7161$, p = 0.4836, n = 11 TH::Cre, 8 DAT::Cre; eYFP: time in social zone – unpaired t test: $t_{15} = 0.8873$, p = 0.3889; social:non-social ratio – $t_{15} = 0.8239$, p = 0.4229, n = 11 TH::Cre, 6 DAT::Cre). Pooling data from TH::Cre and DAT::Cre mice revealed that blue light stimulation caused a significant increase in the time spent in the social zone and social:non-social ratio of ChR2- (time in social zone – paired t test: $t_{18} = 3.877$, **p = 0.0011; social:non-social ratio – $t_{18} = 4.527$, ***p = 0.0003, n = 19), but not eYFP-expressing mice (time in social zone – paired t test: $t_{16} = 0.4094$, p = 0.6876; social:non-social ratio – $t_{16} = 0.5364$, p = 0.5991, n = 17).

(E) In an ICSS task, there was no significant difference in the number of nose pokes made into the active or inactive ports by TH::Cre and DAT::Cre mice expressing ChR2 (active – unpaired t test: $t_{10} = 0.2347$, p = 0.8192; inactive – unpaired t test: $t_{10} = 0.3284$, p = 0.7494, n = 5 TH::Cre, 7 DAT::Cre) or eYFP (active – unpaired t test: $t_{19} = 0.2205$, p = 0.8304; inactive – unpaired t test: $t_9 = 0.7528$, p = 0.4708, n = 7 TH::Cre, 4 DAT::Cre).

(F) The proportion of time spent in the stimulation zone during an RTPA assay was not significantly difference between TH::Cre and DAT::Cre mice expressing ChR2- (unpaired t test: $t_{26} = 0.1678$, p = 0.8681, n = 17 TH::Cre, 11 DAT::Cre) or eYFP (unpaired t test: $t_{15} = 0.5274$, p = 0.6056, n = 11 TH::Cre, 6 DAT::Cre). Pooling data from TH::Cre and DAT::Cre mice revealed that ChR2- but not eYFP-expressing mice spent a significantly greater proportion of time in the unstimulated zone (ChR2 – paired t test: $t_{27} = 4.033$, ***p = 0.0004, n = 28; eYFP: paired t test: $t_{16} = 0.5849$, p = 0.5668, n = 17).

(G) Representative track from a ChR2-expressing and (H) eYFP-expressing TH::Cre mouse in the RTPA assay. ChR2- (paired t test: $t_{16} = 3.248$, **p = 0.005, n = 17), but not eYFP-expressing TH::Cre mice (paired t test: $t_{10} = 0.6779$, p = 0.5132, n = 11) spent a significantly lower proportion of time in the light-paired zone of the chamber.

(I and J) There was no effect of blue light stimulation on locomotion in the open field as measured by total distance traveled in the arena (TH::Cre – two-way ANOVA: group × light interaction: $F_{2,34} = 0.6333$, p = 0.5370, n = 10 ChR2, 9 eYFP; DAT::Cre – two-way ANOVA: group × light interaction: $F_{2,30} = 0.2483$, p = 0.7817, n = 10 ChR2, 7 eYFP). (K-N) Novelty preference was assessed in a modified three-chamber task in which a novel object was placed under one inverted cup instead of a juvenile mouse. Optical stimulation of DRN DA neurons did not significantly affect time spent exploring the novelty zone in (K and L) ChR2- (TH::Cre – paired t test: $t_4 = 0.9499$, p = 0.3959, n = 5; DAT::Cre – paired t test: $t_8 = 0.1215$, p = 0.9063, n = 9) or (M and N) eYFP-expressing mice (TH::Cre – paired t test: $t_5 = 0.8107$, p = 0.4544, n = 6; DAT::Cre – paired t test: $t_5 = 0.6382$, p = 0.5514, n = 6).

(O) Mice were assessed for anxiety-related behavior in the elevated plus maze (EPM) and (P) open field test across a 15 min trial with the middle 5 min paired with blue light stimulation. Representative tracks from a ChR2- (green) and an eYFP-expressing (gray) TH::Cre mouse showing the first ‘light OFF’ and second ‘light ON’ epochs.

(Q–T) There was no significant effect of light stimulation on (Q and S) the time spent in the open arms of the EPM in ChR2- relative to eYFP-expressing mice (TH::Cre - two-way ANOVA: group × light interaction: $F_{2,34} = 3.016$, p = 0.0623, n = 10 ChR2, 9 eYFP; DAT::Cre – two-way ANOVA: group × light interaction: $F_{2,32} = 0.3468$, p = 0.7095, n = 11 ChR2, 7 eYFP) or (R and T) the time spent in the center of the open field (TH::Cre – two-way ANOVA: group × light interaction: $F_{2,34} = 0.1201$, p = 0.8872, n = 10 ChR2, 9 eYFP; DAT::Cre – two-way ANOVA: group × light interaction: $F_{2,30} = 0.2322$, p = 0.7942, n = 10 ChR2, 7 eYFP).

(U and V) In a non-exploratory assay for anxiety-related behavior, the resident-intruder task, blue light stimulation had no effect on total time spent sniffing the juvenile intruder mouse (TH::Cre – ChR2: paired t test: $t_{11} = 0.1924$, p = 0.8510, n = 12; eYFP: $t_{11} = 0.2378$, p = 0.8164; n = 12; DAT::Cre – ChR2: paired t test: $t_6 = 0.01584$, p = 0.9879, n = 7; eYFP: $t_4 = 1.158$, p = 0.3113; n = 5) or (W and X) rearing (TH::Cre – ChR2: $t_{11} = 0.1012$, p = 0.9213; n = 12; eYFP: $t_{11} = 0.1133$, p = 0.9118, n = 12; DAT::Cre – ChR2: paired t test: $t_6 = 0.02656$, p = 0.9797, n = 7; eYFP: $t_4 = 0.9856$, p = 0.3801; n = 5).

Data are represented as mean ± SEM.

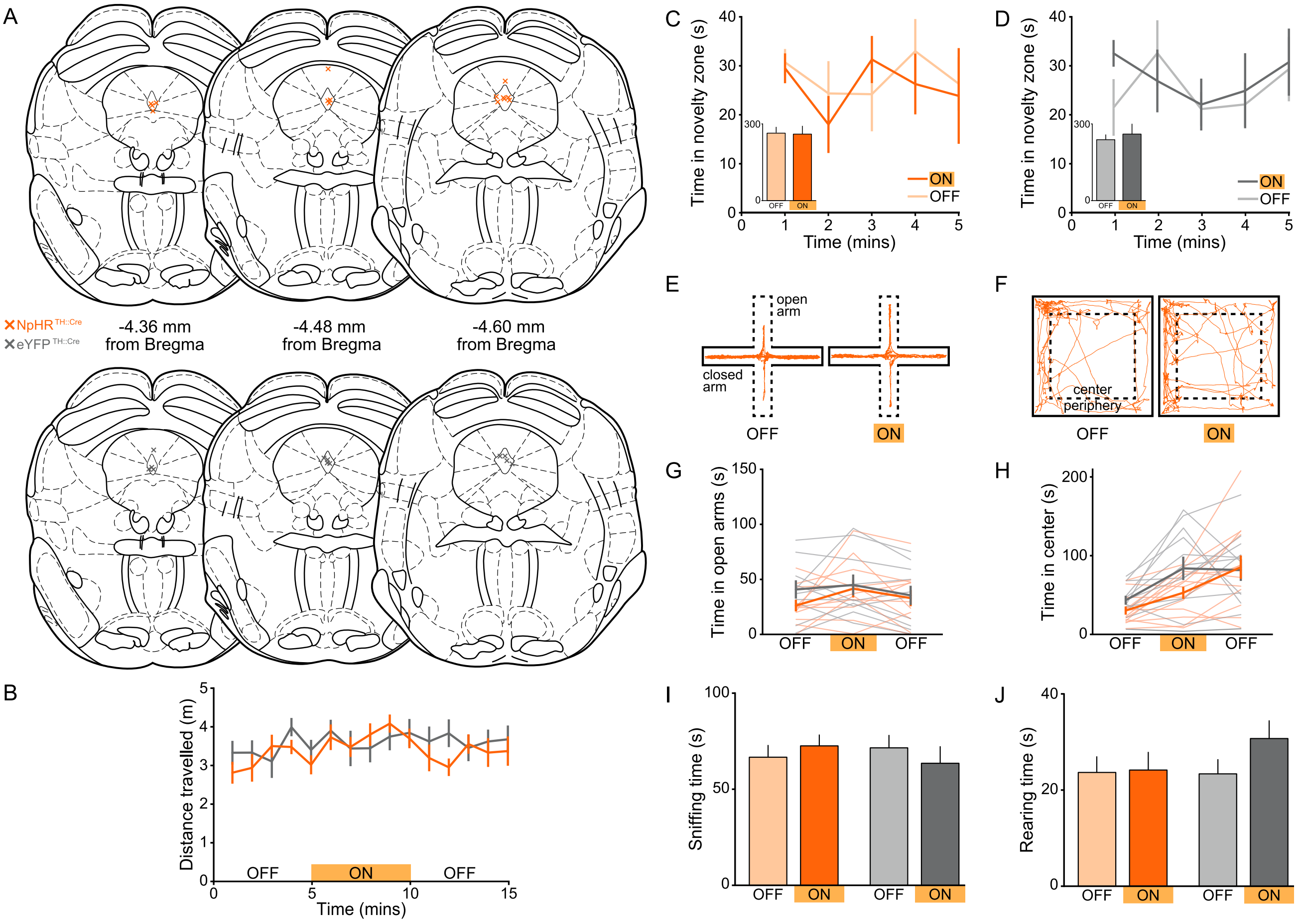

Figure S6. Effect of Photoinhibition in TH::Cre Mice on Behavioral Measures of Arousal and Anxiety-Related Behavior, Related to Figure 6

(A) The position of the optic fiber tip above the DRN of TH::Cre mice expressing NpHR (orange crosses) or eYFP (gray crosses) used for behavioral analysis.

(B) No significant difference was detected between NpHR- and eYFP-expressing mice in distance traveled in the open field.

(C) In an assay for novelty preference, optical inhibition of DRN DA neurons had no effect on the time spent in the novelty zone in NpHR- (paired t test: $t_5 = 0.08958$, $p = 0.9321$, $n = 6$) or (D) eYFP-expressing mice (paired t test: $t_6 = 0.4595$, $p = 0.6620$; $n = 7$).

(E) Representative track from a TH::Cre mouse expressing NpHR in DRN DA neurons in the EPM and (F) open field before light stimulation and during delivery of constant yellow light.

(G) There was no significant effect of photoinhibition on time spent in the open arms of the EPM (two-way ANOVA: group × light interaction: $F_{2,38} = 1.009$, $p = 0.3742$, $n = 11$ NpHR, 10 eYFP) or (H) time spent in the center of the open field (two-way ANOVA: group × light interaction: $F_{2,46} = 2.138$, $p = 0.1294$, $n = 13$ NpHR, 12 eYFP) in NpHR- relative to eYFP-expressing mice.

(I) In the resident-intruder task optical inhibition did not significantly affect time spent sniffing the juvenile (NpHR: paired t test – $t_{13} = 0.8025$, $p = 0.4367$, $n = 14$; eYFP: $t_9 = 1.628$, $p = 0.1380$; $n = 10$) or (J) time spent rearing (NpHR: $t_{13} = 0.0619$, $p = 0.9516$, $n = 14$; eYFP: $t_9 = 1.679$, $p = 0.1274$; $n = 10$).

Data are represented as mean ± SEM.

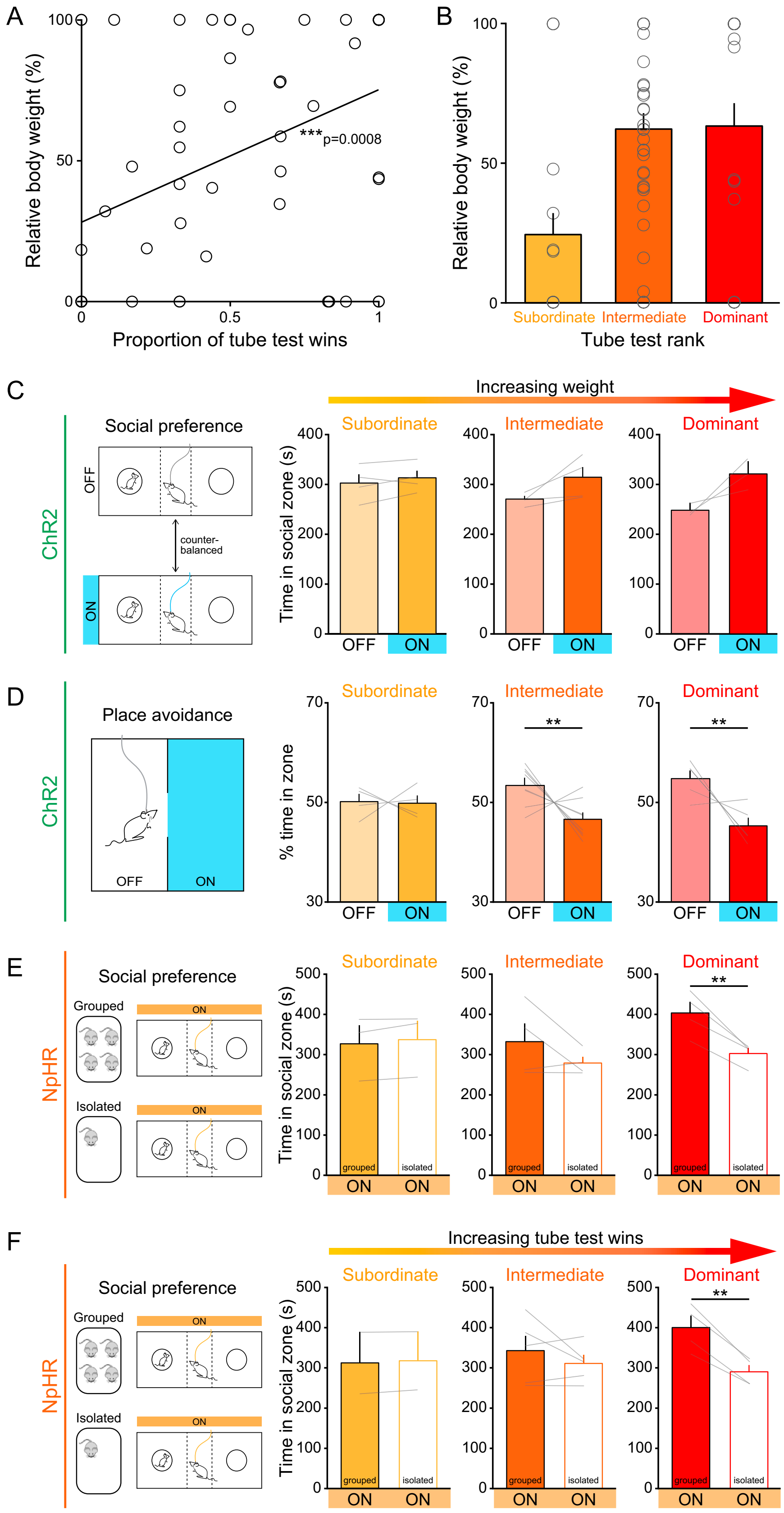

(legend on next page)

Figure S7. Social Rank Predicts the Behavioral Effect of Photoactivation and Photoinhibition of DRN DA Neurons, Related to Figure 7

(A) Proportion of ‘wins’ in the tube test for social dominance plotted against the relative body weight of TH::Cre mice. There was a significant positive correlation between the proportion of wins and body weight (Spearman’s rank correlation: ***$p = 0.0008$, $r^2 = 0.1869$; $n = 57$).

(B) Relative body weight of all mice classified as subordinate, intermediate, and dominant based on performance in the tube test.

(C) Time spent by ChR2-expressing mice in the social zone of the three-chamber apparatus, with and without blue light stimulation, ranked based on relative body weight. (D) % time spent in the light-paired and unstimulated zones in the RTPA assay, by ChR2-expressing mice ranked based on relative body weight.

(E) Time spent by NpHR-expressing mice in the social zone of the three-chamber apparatus when receiving yellow light while group-housed and following social isolation, ranked based on relative weight or (F) proportion of wins in the tube test.

Data are represented as mean +SEM. Paired t tests used for within-group comparisons, *$p < 0.05$, **$p < 0.01$.

Cell, Volume *164*

Supplemental Information

Dorsal Raphe Dopamine Neurons Represent the Experience of Social Isolation

Gillian A. Matthews, Edward H. Nieh, Caitlin M. Vander Weele, Sarah A. Halbert, Roma V. Pradhan, Ariella S. Yosafat, Gordon F. Glober, Ehsan M. Izadmehr, Rain E. Thomas, Gabrielle D. Lacy, Craig P. Wildes, Mark A. Ungless, and Kay M. Tye

SUPPLEMENTAL EXPERIMENTAL PROCEDURES

Methods (for all *ex vivo* recordings in Figures 1 and S1)

Animals and Manipulations

All breeding and experimental procedures were conducted under a project license approved by the Home Office, in accordance with the Animals (Scientific Procedures) Act of 1986 (United Kingdom). 1-4 month-old male, heterozygous tyrosine hydroxylase (TH)-GFP (Sawamoto et al., 2001) or pituitary homeobox 3 (Pitx3)-GFP (Zhao et al., 2004) mice were used to target dopamine (DA) neurons (see (Dougalis et al., 2012) for validation of these mice to target DA neurons in the dorsal raphe nucleus (DRN)). A subset of mice received a single dose of saline or vehicle (0.9% saline with 0.3% Tween-80), administered via intraperitoneal (IP) injection at a volume of 10 mL/kg. Mice were housed in a normal 12 h:12 h light-dark cycle (lights on at 7.00 am) and cage movements and/or injections were conducted at 10.00 am, 24 h before brain slice preparation.

Brain slice preparation

Mice were sacrificed by isoflurane anesthesia followed by decapitation. The brain was rapidly removed out of the cranial cavity and bathed in ice-cold (0-4 °C) artificial cerebrospinal fluid (ACSF, composition in mM, NaCl 120, KCl 3.5, NaH_2PO_4 1.25, $NaHCO_3$ 25, Glucose 10, $MgCl_2$ 1, $CaCl_2$ 2) fully equilibrated with carbogen gas (95% oxygen and 5% carbon dioxide). Two or three coronal brain slices (220 µm thickness) encompassing the DRN were obtained using a vibratome (Leica VT1000S, Leica

Microsystems, Germany). Slices were then maintained in a standard custom-made chamber and were gently and continuously aerated with carbogen gas for at least 1.5 h at room temperature (20-22 °C) before use for electrophysiology. For VTA recordings, two or three horizontal slices were prepared (220 μm thickness).

Electrophysiological recordings

Slices were transferred to a submersion recording chamber and were continuously perfused at a rate of 2-4 mL/min with fully oxygenated ACSF (containing 100 μM picrotoxin) at 30-32 °C. Neurons were visualized using infra-red differential interference contrast (IR-DIC) under an upright microscope (Olympus BXWI 51, Japan) equipped with a 40X objective (0.8 numerical aperture (NA)), an IR filter, DIC optics and a charge coupled device (CCD) video camera (Hamamatsu Photonics, Germany). Neurons were identified as GFP+ using fluorescence illumination (Xcite120 unit, EXFO, UK) coupled to a GFP excitation filter.

Whole-cell patch-clamp recordings were performed with a Multiclamp 700B amplifier (Molecular Devices, CA, USA) and Clampex 10.2 software (Molecular Devices, CA, USA) using glass microelectrodes (5–7 MΩ in resistance) filled with an internal solution containing (in mM): $CsCH_3SO_3$ 125, NaCl 2.8, HEPES 20, EGTA 0.4, TEA-Cl 5, MgATP 2, LiGTP 0.5, and 0.1% neurobiotin. Electrodes were pulled from thin-walled borosilicate glass capillary tubing (GC150F-10, Harvard Apparatus, UK) using a two-stage vertical puller (model PC-10, Narashige, Japan). Recordings were low-pass filtered at 1 kHz and digitized at 3-5 kHz. Series resistance (R_s) and input resistance (R_{in}) were frequently monitored throughout the experiments via a 10 mV, 250 ms

hyperpolarizing step. Any large changes in holding current or noise characteristics were taken as early signs of cell loss and recordings were terminated. Experiments were also terminated if R_s exceeded 35 MΩ or if R_{in} changed more than 15% after break in to whole-cell mode. All potentials cited here have not been corrected for liquid junction potentials (estimated using pClamp calculator as 9.2 mV).

In order to evoke postsynaptic currents, a bipolar stimulating electrode (FHC, USA) was placed 100-300 μm ventrolateral (in coronal slices) or rostral (in horizontal slices) to the recorded neuron, and used to stimulate afferents at 0.03 Hz. Stimulus intensity was controlled using an ISO-flex stimulus isolator (AMPI, Jerusalem, Israel) and adjusted to evoke monosynaptic events. Therefore stimulation only elicited currents with a single peak, and fast rise and decay kinetics. At least 12 stable sweeps were recorded for each protocol, with averaged responses calculated and analyzed using Clampfit 10.2 (Molecular Devices, CA, USA). To determine the AMPAR/NMDAR ratio, the neuron was voltage-clamped at +40 mV and a stable mixed AMPAR- and NMDAR-mediated excitatory postsynaptic current (EPSC) recorded. The NMDAR antagonist d-AP5 (50 μM; Tocris Bioscience, UK) was then applied to the slice, and the pure AMPAR current recorded. This was then digitally subtracted from the mixed current using ClampFit 10.2 to give the pure NMDAR current, and the AMPAR/NMDAR ratio was calculated by dividing the peak amplitude of the average AMPAR-mediated EPSC by the peak amplitude of the NMDAR-mediated EPSC. For analysis of correlation between AMPAR/NMDAR ratio and number of mice per cage, an average AMPAR/NMDAR ratio was calculated for each mouse.

For paired-pulse experiments an inter-stimulus interval of 50 ms was used, and paired stimuli were delivered every 30 s. The paired-pulse ratio (PPR) was calculated by dividing the average peak amplitude of the EPSC generated by the second stimulus by the average peak amplitude generated by the first. For rectification indices (RIs), and a subset of the PPRs, 0.1 mM spermine was included in the internal solution. To calculate the RI, 50 μM d-AP5 was added to the ACSF and the AMPAR current recorded while the neuron was voltage-clamped at -70 mV, 0 mV, and +40 mV. The RI was calculated by dividing the average peak of the AMPAR current recorded at -70 mV by the average peak recorded at +40 mV. NASPM (25 μM; Sigma, UK) was applied to the slice while stimulating EPSCs at -50 mV every 30 s. A stable baseline was recorded for at least 5 min followed by a 5 min application of NASPM. The average amplitude of the EPSC was calculated during the 5 min immediately prior to NASPM application (baseline) and the 5 min immediately following drug application, with the EPSC amplitude normalized to the baseline period. The decay kinetics of the NMDAR-mediated current recorded at +40 mV was determined by fitting the decay phase of the current with a double exponential function using Spike 2 (Cambridge Electronic Design, UK; Figure S1H). The following formula was used to calculate the weighted decay time constant (τ_W) of the NMDAR-mediated current:

$$\tau_W = [(A_1 \times \tau_1) + (A_2 \times \tau_2)]/[(A_1 + A_2)]$$

A_1 and A_2 are the amplitudes, and τ_1 and τ_2 the decay time constants of the fast and slow components, respectively (Lammel et al., 2011; Vicini et al., 1998).

Immunohistochemistry

Following recording brain slices were incubated in 4% w/v paraformaldehyde (PFA), in phosphate-buffered saline (PBS) overnight at 4 ºC (or 45 min at room temperature). Fixed free-floating sections were subsequently washed in PBS containing 0.2% Triton X-100 (PBS-T 0.2%), then blocked in PBS-T 0.2% with 6% normal donkey serum (NDS; Jackson ImmunoResearch, USA) for 30-60 min at room temperature. They were then incubated overnight at room temperature in PBS-T 0.2% containing 2% NDS and primary antibodies: anti-TH polyclonal chicken antibody (1:1000, Abcam ab76442, USA); anti-VIP (vasoactive-intestinal polypeptide) rabbit antibody (1:500, Immunostar 20077, USA). See (Dougalis et al., 2012) and references therein for discussion of validation of these antibodies. Brain slices were then washed 4 times in PBS-T 0.2% and incubated for 90 min at room temperature in PBS-T 0.2% containing 2% NDS and appropriate secondary antibodies: Cy3-conjugated anti-rabbit (1:1000, Jackson ImmunoResearch 711-165-152, USA) and Alexa Fluor 633-conjugated anti-chicken (1:1000, Invitrogen A21103, USA). AMCA-conjugated streptavidin (1:1000, Jackson ImmunoResearch 016-150-084, USA) was used to reveal neurobiotin labeling. Brain slices were finally washed 3 times in PBS-T 0.2%, followed by twice in PBS, then mounted onto glass microscope slides, and coverslipped using VectaShield mounting medium (Vector Laboratories, USA). Confocal laser scanning microscopy was performed using a Leica SP5 II confocal microscope (Leica Microsystems, Germany) through a 20X / 0.7 NA dry HC Plan-Apochromat CS DIC objective, with 1.5X digital zoom applied during image capture (30X total magnification). GFP was excited by a 488 nm line of an Argon laser, Cy3 by a 561 nm line of a DPSS laser, Alexa Fluor 633 by a

633 nm line of a HeNE laser, and AMCA by a 405 nm line of a diode laser. Images were captured at a resolution of 2048 x 2048 and were processed with general brightness and contrast curve adjustments in Adobe Photoshop CS5 (Adobe Systems Incorporated, CA, USA).

We observed a significant increase in the AMPAR/NMDAR ratio following 24 h of social isolation in GFP+ DRN neurons from TH-GFP and Pitx3-GFP mice (Figure S1A), and also in immunohistochemically-identified GFP+/TH+ and GFP+/TH- neurons (Figure S1B). We also found that both VIP+ and VIP- DRN DA neurons (Dougalis et al., 2012) displayed a significantly greater AMPAR/NMDAR ratio following social isolation compared with group-housed mice (Figures S1D and S1E). Furthermore, 24 h of social isolation significantly increased the AMPAR/NDMAR ratio in both adolescent (<postnatal day (PN)50) and adult (>PN50) mice (Figure S1C). Consequently, data from all GFP+ neurons, in 1-4 month-old mice from both transgenic lines, was pooled. Data presented in Figure S1A-E and Figure 7A-B includes all mice which either remained group-housed, or were socially isolated, including a subset of mice which received an IP injection of saline or vehicle 24 h prior to recording.

Statistics

Statistical analysis was performed using GraphPad Prism (GraphPad Software, Inc., CA, USA), and values are reported as mean ± standard error of the mean (S.E.M.). Two experimental groups were compared using an unpaired t-test (non-directional) or Mann-Whitney U test, and three or more groups were compared using a one-way ANOVA with Newman-Keuls post-hoc tests to control for multiple comparisons. The Pearson's product-moment correlation coefficient was used to analyze correlation between two variables. Group sizes were based those used in previous studies to obtain significant differences using similar measures.

Methods (for all stereotaxic surgeries, FSCV, *ex vivo* recordings, behavioral testing, and fiber photometry in Figures 2-7 and S2-S7)

Animals

All experiments involving the use of animals were in accordance with NIH guidelines and approved by the MIT Institutional Animal Care and Use Committee. Male heterozygous TH::IRES-Cre (Lindeberg et al., 2004), DAT::IRES-Cre (Bäckman et al., 2006), VGLUT2::IRES-Cre (Vong et al., 2011), VGAT::IRES-Cre (Vong et al., 2011), and wild-type C57BL/6 mice (Jackson Laboratory, ME, USA) were housed on a 12 h: 12 h reverse light/dark cycle (lights off at 09.00 am) with food and water available *ad libitum*.

Stereotaxic Surgery

Surgery was performed on mice between 6-8 weeks of age. Mice were anesthetized with isoflurane (4% for induction, 1.5-2% thereafter for maintenance) and then placed in a small animal stereotax (David Kopf Instruments, CA, USA). All skull measurements were made relative to Bregma, unless otherwise noted. Viral injection into the DRN was performed using a beveled 33 gauge microinjection needle (facing medially) with a 10 µL microsyringe (Nanofil; WPI, FL, USA) delivering the virus at a rate of 0.1 µL/min using a microsyringe pump (UMP3; WPI, FL, USA) and controller (Micro4; WPI, FL, USA). Following viral injection, the needle was held at the injection site for 1-2 min, then raised up by 0.05 mm and held for a further 10 min to allow diffusion of the virus. The needle was then slowly withdrawn.

For behavioral experiments, 0.3-0.5 µL of the anterogradely travelling adeno-associated virus serotype 5 (AAV_5), encoding channelrhodopsin-2 (ChR2)-eYFP, under a double-floxed inverted open-reading frame construct (DIO) (AAV_5-EF1α-DIO-ChR2(H134R)-eYFP), or enhanced halorhodopsin 3.0 (AAV_5-EF1α-DIO-eNpHR3.0-eYFP) (Gradinaru et al., 2008), or eYFP alone (AAV_5-EF1α-DIO-eYFP) was injected into the DRN (at a 20° angle from the right side to avoid the aqueduct; AP: -4.10; ML: 1.25; DV: -2.90). For fiber photometry recordings, an AAV_5 encoding the calcium indicator, GCaMP6m, under a FLEX construct and the CAG promoter (AAV_5-CAG-FLEX-GCaMP6m) was injected into the DRN. In TH::Cre and DAT::Cre mice this resulted in eYFP expression in TH+ neurons within the DRN, ventrolateral periaqueductal grey, caudal linear and rostral linear nuclei. However, for *in vivo* manipulations or recording the optic fiber was always positioned over the DRN (Figures S2, S5, and S6).

In order to examine downstream release of glutamate in brain slices *ex vivo* and release of DA *in vivo*, TH::Cre and DAT::Cre mice received an injection of 0.3-0.5 µL AAV_5-EF1α-DIO-ChR2-eYFP into the DRN (AP: -4.10; ML: 1.25; DV: -2.90; at a 20° angle from the right side). Additionally, to determine co-expression of VGLUT2 or VGAT with TH in the DRN, 0.5 µL AAV_5-EF1α-DIO-ChR2-eYFP was injected into the DRN (AP: -4.10; ML: 1.25; DV: 2.90; at a 20° angle from the right side) of VGLUT2::Cre and VGAT::Cre mice.

For *in vivo* optogenetic manipulations, a manually-constructed optic fiber (300 µm core, NA=0.37 (Thorlabs, NJ, USA) (Sparta et al., 2012)) held in a stainless steel ferrule (Precision Fiber Products, CA, USA) was implanted directly above the DRN (AP: -4.05

to -4.10; ML: 0.00; DV: -2.40 to -2.45). For photometry experiments, an optic fiber was implanted within the DRN (AP: -3.90 to -4.20; ML: 0.00; DV: -2.50 to -2.80). A layer of adhesive cement (C&B Metabond; Parkell Inc., NY, USA) followed by cranioplastic cement (Ortho-Jet; Lang, IL, USA) was used to secure the optic fiber to the skull, which was allowed to dry completely before closure of the incision with nylon sutures. Mice were maintained under a heat lamp until they had fully recovered from anesthesia.

Viral constructs

Recombinant AAV vectors containing ChR2 or NpHR were serotyped with AAV_5 coat proteins and packaged by the University of North Carolina Vector Core (Chapel Hill, NC, USA). Viruses carrying GCaMP6m were packaged by the University of Pennsylvania Vector Core (Philadelphia, PA, USA).

Behavioral Experiments with Optogenetic Manipulations

Behavioral testing was performed at least 4 weeks following viral injection to allow sufficient time for transgene expression. Mice were tested during their active dark phase (09.00 am – 5:00 pm) and were given at least 1 h in the behavioral testing room to acclimate before experiments commenced. For experiments involving social isolation, mice were isolated between 9.00 am – 10.00 am and behavioral testing was performed 24-28 h later. Mice were isolated in their home cage or in a clean cage with some of the previous home cage bedding. Optic fiber implants were connected to a patch cable using a ceramic sleeve (PFP, CA, USA), which in turn was connected to a commutator (rotary joint; Doric, Québec, Canada) by means of an FC/PC adapter to allow

unrestricted movement. A second patch cable, with an FC/PC connector at either end (Doric, Québec, Canada), connected the commutator to a 473 nm or 593 nm diode-pumped solid state (DPSS) laser (OEM Laser Systems, UT, USA). A Master-8 pulse stimulator (A.M.P.I., Jerusalem, Israel) was used to control the output of the 473 nm laser using a light power of 20 mW, and the 593 nm laser using a light power of 1-5 mW.

Three-Chamber Sociability Test: Based on the three-chamber task design previously described (Felix-Ortiz and Tye, 2014; Moy et al., 2004), each experimental mouse was placed in a (57.15 x 22.5 x 30.5 cm) chamber consisting of four transparent Plexiglas walls and grey plastic floors. Within the chamber there were three compartments which the mice could move freely between: left and right compartments (each 24.5 x 22.5 cm) and a smaller center compartment (8 x 22.5 cm). The left and right compartments each contained an inverted wire cup. Mice were first subjected to a 10 min habituation period to acquaint them to the apparatus. Mice which displayed a strong preference for one side of the chamber (>70%) during this period were excluded from the analysis. Following habituation, the test mouse was contained within the center compartment for 1-2 min while a juvenile male C57BL/6 mouse (3.5-5 weeks of age) was placed under one of the two inverted wire cups (counterbalanced). The test mouse was then allowed to freely move between the compartments for a further 10 min. This test was repeated 24 h later using a different juvenile mouse and with the chamber rotated 90° to change external spatial cues. The 10 min phase in which the juvenile mouse was present was paired with optical stimulation (ChR2: 473 nm, 8 pulses of 5 ms pulse-width, at 30 Hz, every 5 s; NpHR: 593 nm, constant) on one of the two days

(counterbalanced across groups). A video camera located directly above the arena recorded each phase with mouse movement tracked using EthoVision XT (Noldus, Wageningen, Netherlands). Heat maps were generated in Ethovision XT with pseudo-color representing the relative time spent by the mouse at each position, with the maximum and minimum calculated within each trial. The social:non-social ratio was calculated by dividing the total time spent in the social zone (containing the juvenile mouse) by the time spent in the non-social zone.

Three-Chamber Novelty Preference Test: To test for novelty preference the three-chamber task was conducted (as described above for sociability), except instead of a juvenile mouse, a novel object was placed under one of the two inverted cups. The 10 min phase in the presence of the novel object was paired with optical stimulation (ChR2: 473 nm, 8 pulses of 5 ms pulse-width, at 30 Hz, every 5 s; NpHR: 593 nm, constant) on one of the two days (counter-balanced across groups).

Intra-Cranial Self-Stimulation: Mice were removed from *ad libitum* food 14-18 h prior to testing to facilitate behavioral responding. Single mice were placed in a Med Associates operant chamber (Med Associates, VT, USA) containing an active and inactive nose poke port, which were both illuminated. Low volume white noise was played into the chamber to mask extraneous sounds. A nose poke into either port was accompanied by illumination of a cue-light (positioned above the nose poke port) and a distinct 1 s tone (1 or 1.5 kHz) played into the box. A nose poke into the active port also resulted in delivery of a 30 Hz train of 5 ms pulse-width 473 nm light for 3 s, whereas a nose poke into the inactive port resulted in no stimulation. The physical location and associated tone of the nose poke ports were counterbalanced between groups. Mice

were allowed to explore the chamber for 2 h, with nose poke activity recorded with MedPC software (Med Associates, VT, USA), and subsequently analyzed using MATLAB (Mathworks, MA, USA). On the first training day, both operanda were baited with identical palatable odor cues to encourage investigation. On the second test day, the operanda were not baited.

Real-Time Place Aversion: Individual mice were placed in a transparent Plexiglas chamber (50 x 53 cm divided into left and right compartments, or 57.15 x 22.5 x 30.5 cm divided into left, right, and center compartments). They were allowed to freely move between compartments for 1 h, during which entry into one of the two sides resulted in photostimulation (473 nm light, 15 pulses of 5 ms pulse-width, at 30 Hz, every 5 s). The side paired with photostimulation was counterbalanced between animals. A video camera positioned directly above the arena was used to track mouse movement (EthoVision XT, Noldus, Wageningen, Netherlands). The first 30 min of exploration was used for analysis.

Conditioned-Place Preference: Single mice were placed in a (57.15 x 22.5 x 30.5 cm) chamber consisting of left and right compartments (each 24.5 x 22.5 cm) with rough or smooth grey plastic floors and a center compartment (8 x 22.5 cm) with a punched metal floor. Left and right compartment walls were either thin black and white vertical stripes or thick black and white horizontal stripes (counterbalanced across animals). On day 1, mice were exposed to a 15 min habituation phase in which they were allowed to freely explore the chamber. Mice with a strong initial preference for either side of the chamber (>70%) were excluded from the analysis. On day 2, mice were exposed to two 30 min conditioning sessions (separated by at least 2 h) during which they were

confined to one side of the chamber and received optical stimulation (15 pulses of 5 ms pulse-width 473 nm light, at 30 Hz, every 5 s) or no stimulation (counterbalanced for order and side across animals). On day 3, mice were placed in the chamber and allowed to freely explore in the absence of optical stimulation for 45 min, with the first 10 min used for analysis. A video camera positioned directly above the arena was used to track mouse movement (EthoVision XT, Noldus, Wageningen, Netherlands). Side preference was calculated by dividing the time spent in the compartment paired with optical stimulation by the total time spent in both compartments.

Open Field Test: Individual mice were placed in a 50 x 53 cm arena composed of four transparent Plexiglas walls. They were allowed to freely move throughout the arena for 15 min, with light stimulation occurring during the middle 5 min epoch (ChR2: 473 nm, 8 pulses of 5 ms pulse-width, at 30 Hz, every 5 s; NpHR: 593 nm, constant). A video camera positioned directly above the arena was used to track the movement of each mouse (EthoVision XT, Noldus, Wageningen, Netherlands). In order to assess anxiety-related behavior (Carola et al., 2002), the chamber was divided into a center (36 x 37 cm) and periphery region.

Elevated Plus Maze: The maze was elevated from the ground by 75 cm and consisted of two closed arms (30 x 5 x 30 cm) and two open arms (30 x 5 cm), emanating at 90° from each other from a central platform (5 x 5 cm), all made of grey plastic. Mice were placed on one of the open arms of the maze and allowed to freely explore for 15 min, with light stimulation occurring during the middle 5 min epoch (ChR2: 473 nm, 8 pulses of 5 ms pulse-width, at 30 Hz, every 5 s; NpHR: 593 nm,

constant). A video camera positioned directly above the arena was used to track the movement of each mouse (EthoVision XT, Noldus, Wageningen, Netherlands).

Resident-Intruder Assay: Mice were individually recorded in their home cage for a total of 9 min. After the first 2 min, photostimulation (ChR2: 473 nm, 8 pulses of 5 ms pulse-width, at 30 Hz, every 5 s; NpHR: 593 nm, constant) commenced. After a further 3 min, a juvenile mouse (3.5-4.5 weeks of age) was placed in the cage with the test mouse. Following 3 min of interaction photostimulation ceased and 1 min later the juvenile was removed. The behavior of the test mouse across the 3 min immediately following introduction of the juvenile intruder was manually scored twice by two different experimenters, blind to the experimental conditions, using ODLog behavioral analysis software (Macropod Inc., USA). The resident-intruder assay was performed twice, 24 h apart, with a different juvenile mouse used on each day, and with one day paired with photostimulation (order counterbalanced across groups). Notably, this assay was designed to examine anxiety-related behavior (File and Seth, 2003) rather than social interest, as the addition of an intruder to the home cage of a male may be interpreted as a threat.

Estimated Social Rank: In ChR2-expressing TH::Cre mice, relative social dominance was estimated based on relative body weight within the cage, whereby the heaviest mouse within a given cage was assigned a relative dominance score of ‘1’, the lightest mouse a score of ‘0’, and intermediate mice a score between 0-1 based on their relative weight. In addition, in a subset of mice, the tube test for social dominance was performed, similar to previously described (Lindzey et al., 1961; Wang et al., 2011). A transparent Plexiglas tube, 30 cm in length and 3.2 cm or 3.8 cm inside diameter

(dependent on mouse size) was used, which was sufficient to allow a single mouse to pass through uninhibited. Mice were exposed to 4 days of training, with eight trials on days 1-2 and 3 trials on days 3-4. For training trials, mice were released into the tube, at alternating ends, and allowed to run through and exit at the opposite end. Mice which were reluctant to pass all the way through, or attempted to reverse, were encouraged with a plastic stick gently pressing on their hind region. Following training, mice were then tested against each of their cagemates, in a round-robin design, each day for three consecutive days. For each test trial, two mice were simultaneously released at either end of the tube, and the mouse which was the first to retreat from the tube was designated as the 'loser', while his opponent was designated as the 'winner'. Trials in which the mice did not meet in the center of the tube were repeated. The order in which mice were tested against each other and the side from which they were released were counterbalanced across each day of trials. The relative proportion of 'wins' was calculated for each mouse across the three days of testing, by taking the number of 'wins' divided by the total number of contests. For NpHR-expressing mice, and a subset of ChR2-expressing mice, the proportion of tube test 'wins' was taken into account, in addition to relative body weight, so that their relative dominance score reflected an average of both of these measures.

Fiber Photometry

For the photometry system, blue light from a 473 nm DPSS laser (60-160 µW; OEM Laser Systems, UT, USA) was filtered through a neutral density filter (1.0 optical density, Thorlabs, NJ, USA) held in a filter wheel (FW1A, Thorlabs, NJ, USA), chopped

at 400±10 Hz (Model SR540 Chopper Controller, Stanford Research Systems, CA, USA) through a filter (LD01-473, Semrock, NY, USA) and reflected off of a dichroic mirror (FF495, Semrock, NY, USA) and coupled through a fiber collimation package (F240FC-A, Thorlabs, NJ, USA) into a patch cable connected to the implanted ferrule via a ceramic sleeve (PFP, CA, USA). GCaMP6m fluorescence was collected through a 525 nm bandpass filter (FF03-525, Semrock, NY, USA) into a photodetector (Model 2151, Newport, CA, USA). The signal was passed through a lock-in amplifier (100 ms, 12 dB, 500 mV, Model SR810, Stanford Research Systems, CA, USA) and digitized and collected with a LabJack U6-PRO (250 Hz sampling frequency, LabJack, CO, USA). The raw signal over the entire session was divided by a linear fit to normalize the baseline over the trial. Z-scores were taken using the period -5 to -2 s prior to the event detections as baseline.

Ethovision XT (Noldus, Wageningen, Netherlands) was used to collect video footage of the test mouse, and a TTL pulse was sent to the LabJack U6-PRO to time-lock the video with the photometry data. Mice (either group-housed or socially isolated for 24 h) were placed in their home cage and fluorescence was recorded for 5 min, after which a juvenile intruder or novel object was placed into the cage. Bouts of interaction with either the juvenile intruder or novel object were scored manually by experimenters blind to the experimental conditions.

Fast-Scan Cyclic Voltammetry (FSCV)

TH::Cre mice, which had received an injection of 0.3-0.5 µL AAV_5-DIO-ChR2-eYFP in the DRN, were given at least 8 weeks for viral expression before recording experiments. Anesthetized *in vivo* FSCV experiments were conducted similar to those

previously described (Tsai et al., 2009). Briefly, mice were anesthetized with urethane (1.5 g/kg; IP) and placed in a stereotaxic frame. Craniotomies were performed above the BNST (AP: +0.20 mm, ML: +1.00 mm), CeA (AP: -0.75 mm, ML: +2.35 mm), DRN (AP: -4.15, ML: 0.00 mm), and contralateral cortex. An Ag/AgCl reference electrode was implanted in the contralateral cortex and a 300 µm optical fiber was implanted above the DRN (DV: - 2.45 mm). Both implants were secured to the skull with adhesive cement (C&B Metabond; Parkell, NY, USA). A glass-encased carbon fiber electrode (~120 µm in length, epoxied seal) was lowered into the BNST (DV: -3.00 mm from brain surface) or CeA (DV: -3.20 mm from brain surface) for electrochemical recordings. Electrodes were allowed to equilibrate for 20 min at 60 Hz and 10 min at 10 Hz. Voltammetric recordings were collected at 10 Hz by applying a triangular waveform (-0.4 V to +1.3 V to -0.4 V, 400 V/s) to the carbon-fiber electrode versus the Ag/AgCl reference. DA release was evoked by optical stimulation of the DRN using 150 pulses of 473 nm light (25 mW, 5 ms pulse duration) at 20, 30, or 50 Hz, delivered via a DPSS laser (OEM Laser Systems, UT, USA) and controlled using a Master-8 pulse stimulator (A.M.P.I., Jerusalem, Israel). Data were collected using Tarheel CV (NC, USA) in 15 s files with the stimulation onset occurring 5 s into the file. Files were collected every 60 s and background subtracted at the lowest current value prior to stimulation onset. Light-evoked signals maintained characteristic cyclic voltammograms for DA (unless otherwise noted in text), with oxidation and reduction peaks at ~+0.65 V and ~-0.2 V, respectively.

A group of 10 carbon-fiber electrodes were calibrated in known concentrations of DA (250 nM, 500 nM, and 1 µM) and mean calibration data were used to convert *in vivo*

signals to changes in DA concentration using chemometric, principal component regression, and residual analyses using a custom LabView program (provided by R. Keithley). Following recordings, mice were transcardially perfused with 4% PFA (described below) and processed using immunohistochemistry to confirm viral expression and placement of the optic fiber and recording electrodes. Stimulation recordings collected at 10 Hz were individually binned in 0.5 s bins for visualization. Evoked DA release was quantified by calculating the area under the curve and peak evoked release for each recording.

Ex Vivo **Electrophysiological Recordings in the BNST and CeA**

TH::Cre or DAT::Cre mice received an injection of 0.3-0.5 µL AAV_5-DIO-ChR2-eYFP into the DRN and at least 8 weeks was allowed for viral expression. Mice were deeply anesthetized with sodium pentobarbital (200 mg/kg; IP) then transcardially perfused with 20 mL ice-cold modified ACSF (composition in mM: NaCl 87, KCl 2.5, NaH2PO4*H20 1.3, MgCl2*6H2O 7, NaHCO3 25, sucrose 75, ascorbate 5, CaCl2*2H2O 0.5, in ddH20; osmolarity 320-330 mOsm, pH 7.30-7.40) saturated with carbogen gas (95% oxygen, 5% carbon dioxide). The brain was rapidly removed from the cranial cavity and sectioned on a vibrating-blade microtome (Leica VT1000S, Leica Microsystems, Germany). Coronal 300 µm brain slices were prepared containing the DRN, the BNST, and the CeA. Slices were given at least 1.5 h to recover in a holding chamber containing ACSF (composition in mM: NaCl 126, KCl 2.5; NaH2PO4*H20 1.25, MgCl2*6H2O 1, NaHCO3 26, glucose 10, CaCl2*2H2O 2.4, in ddH20; osmolarity 299-301 mOsm; pH 7.30-7.40) saturated with carbogen gas at 32 °C before being transferred to the recording chamber for electrophysiology. In the recording chamber,

slices were continuously perfused at a rate of 2 mL/min with fully oxygenated ACSF at 30-32 °C.

For whole-cell patch-clamp electrophysiology, electrodes were pulled from thin-walled borosilicate glass capillary tubing on a P-97 puller (Sutter Instrument, CA, USA) and had resistances of 4-7 MΩ when filled with internal solution (composition in mM: $CsCH_3SO_3$ 117, NaCl 2.8, HEPES 20, EGTA 0.4, TEA-Cl 5, MgATP 4, Na-GTP 0.3, QX-314 5, spermine 0.1, and 0.3% biocytin, in ddH20; osmolarity 287 mOsm; pH 7.30). Recordings were made using a Multiclamp 700B amplifier and Clampex 10.4 software (Molecular Devices, CA, USA). Signals were low-pass filtered at 1 kHz and digitized at 10 kHz using a Digidata 1550 (Molecular Devices, CA, USA). Throughout recording, capacitance, R_s, and R_{in} were frequently measured to monitor cell health. Neurons were visualized via a 40X water-immersion objective on an upright microscope (Scientifica, UK) equipped with IR-DIC optics and a Q-imaging Retiga Exi camera (Q Imaging, Canada). The region of terminal expression in the BNST and CeA was identified by brief illumination through a 470 nm LED light source (pE-100; CoolLED, NY, USA). Neurons were recorded in voltage-clamp mode with a holding potential of -70 mV and 0 mV to elicit EPSCs and IPSCs, respectively. ChR2 was activated by a 5 ms pulse of 470 nm LED light, delivered through the objective, every 20 s. In order to confirm the existence of a monosynaptic connection, in a subset of cells, the optically-evoked current was recorded in the presence of tetrodotoxin (TTX; 1 μM) and 4-aminopyridine (4AP; 0.5-1 mM) (Petreanu et al., 2009).

For recording ChR2-expressing neurons within the DRN, an internal solution was used containing (in mM): potassium gluconate 125, NaCl 10, HEPES 20, MgATP 3, and

0.3% biocytin, in ddH20 (osmolarity 287 mOsm; pH 7.33). Neurons were recorded in current-clamp mode and ChR2 was activated by 8 pulses of 470 nm LED light (5 ms pulse duration) delivered at 30 Hz every 5 s.

Subsequent analysis was performed using Clampfit 10.4 software (Molecular Devices, CA, USA) with at least 12 sweeps used to calculate the average light-evoked EPSC. Amplitude and latency were estimated in Clampfit, with latency taken as the time from light onset to the initial downward deflection of the EPSC.

Immunohistochemistry

Mice were deeply anesthetized with sodium pentobarbital (200 mg/kg; IP) and then transcardially perfused with 20 mL ice-cold (~4 °C) Ringers solution followed by 20 mL ice-cold 4% PFA in PBS. The brain was then dissected out of the cranial cavity and placed in 4% PFA solution at 4 °C for 5-18 h before being transferred to 30% sucrose solution for at least 36 h at 4 °C. Brains were sectioned at 40 µm thickness on a freezing microtome (HM430; Thermo Fisher Scientific, MA, USA) and sections were subsequently stored in PBS at 4 °C. For immunohistochemistry sections were blocked in PBS-T 0.3% with 3% NDS (Jackson ImmunoResearch, USA) for 1 h at room temperature followed by incubation in primary antibody solution: chicken anti-TH (1:1000; Millipore, USA) and rabbit anti-5-HT (1:2000; Immunostar, WI, USA) in PBS-T 0.3% with 3% NDS for 48 h at 4 °C. Sections were subsequently washed 4 times in PBS (for 10 min each) and then transferred to secondary antibody solution: Alexa 647-conjugated donkey anti-chicken (1:500-1:1000), Cy3-conjugated donkey anti-rabbit (1:1000), and a DNA-specific fluorescent probe (DAPI; 1:50,000) in PBS-T 0.1% with

3% NDS for 2 h at room temperature. Sections were again washed 4 times in PBS (for 10 min each) before being mounted onto glass slides and coverslipped using polyvinyl alcohol mounting medium with DABCO (Sigma, MO, USA).

Confocal Microscopy

Fluorescent images were captured using a confocal laser scanning microscope (Olympus FV1000), with FluoView software (Olympus, PA, USA), under a 10X / 0.40 NA dry objective or a 40X /1.30 NA oil immersion objective. The location of viral expression at the injection site, the lesion from the optic fiber placement, and the position of carbon-fiber recording electrodes were determined by taking serial z-stack images through the 10X objective across a depth of at least 20 μm, with an optical slice thickness of 5 μm. High magnification images of eYFP-expressing cells under the optic fiber tip were obtained through the 40X objective using serial z-stack images with an optical slice thickness of 2 μm. Images were subsequently processed in Adobe Photoshop CS6 (Adobe Systems Incorporated, CA, USA).

Immunohistochemical Quantification

At least 4 weeks following viral injection, VGLUT2::Cre and VGAT::Cre mice were transcardially perfused-fixed and the brains processed using immunohistochemistry (as described above). High magnification images of eYFP+ cells in the DRN were obtained, within the region containing TH+ neurons. Two VGLUT2::Cre and two VGAT::Cre mice were analyzed using 2 sections per animal. The numbers of TH+ and eYFP+/TH+ neurons within the region were counted by an experimenter blind to the experimental condition.

In TH::Cre and DAT::Cre mice the proportion of eYFP+ cells co-labeled with TH in the DRN was determined from counts performed on 1-2 sections per animal, in which the lesion from the tip of the optic fiber was visible (n=8 mice for TH::Cre and n=6 mice for DAT::Cre). In order to determine eYFP/5-HT co-expression one anterior and one posterior section containing the DRN was counted from each animal (n=3 TH::Cre and n=3 DAT::Cre). Cell counting was performed using custom written software in MATLAB (Mathworks, Natick, MA, USA).

Statistics

Statistical analyses were performed using using GraphPad Prism (GraphPad Software, Inc., CA, USA), OriginPro 8.6 (OriginLab, MA, USA), and MATLAB (Mathworks, MA, USA). Group comparisons were made using two-way repeated measures ANOVA followed by Bonferroni post-hoc tests to control for multiple comparisons. Paired and unpaired t-tests, as well as one-way repeated measures ANOVAs were used to make single-variable comparisons, and the Chi-squared test was used to compare populations. The Pearson's product-moment correlation coefficient or the Spearman's rank correlation coefficient was used to analyze correlation between two variables.

SUPPLEMENTAL REFERENCES

Bäckman, C.M., Malik, N., Zhang, Y., Shan, L., Grinberg, A., Hoffer, B.J., Westphal, H., and Tomac, A.C. (2006). Characterization of a mouse strain expressing Cre recombinase from the 3' untranslated region of the dopamine transporter locus. Genes. N. Y. N 2000 *44*, 383–390.

Carola, V., D'Olimpio, F., Brunamonti, E., Mangia, F., and Renzi, P. (2002). Evaluation of the elevated plus-maze and open-field tests for the assessment of anxiety-related behaviour in inbred mice. Behav. Brain Res. *134*, 49–57.

Dougalis, A.G., Matthews, G.A.C., Bishop, M.W., Brischoux, F., Kobayashi, K., and Ungless, M.A. (2012). Functional properties of dopamine neurons and co-expression of vasoactive intestinal polypeptide in the dorsal raphe nucleus and ventro-lateral periaqueductal grey. Eur. J. Neurosci. *36*, 3322–3332.

Felix-Ortiz, A.C., and Tye, K.M. (2014). Amygdala inputs to the ventral hippocampus bidirectionally modulate social behavior. J. Neurosci. Off. J. Soc. Neurosci. *34*, 586–595.

File, S.E., and Seth, P. (2003). A review of 25 years of the social interaction test. Eur. J. Pharmacol. *463*, 35–53.

Gradinaru, V., Thompson, K.R., and Deisseroth, K. (2008). eNpHR: a Natronomonas halorhodopsin enhanced for optogenetic applications. Brain Cell Biol. *36*, 129–139.

Lammel, S., Ion, D.I., Roeper, J., and Malenka, R.C. (2011). Projection-specific modulation of dopamine neuron synapses by aversive and rewarding stimuli. Neuron *70*, 855–862.

Lindeberg, J., Usoskin, D., Bengtsson, H., Gustafsson, A., Kylberg, A., Söderström, S., and Ebendal, T. (2004). Transgenic expression of Cre recombinase from the tyrosine hydroxylase locus. Genes. N. Y. N 2000 *40*, 67–73.

Lindzey, G., Winston, H., and Manosevitz, M. (1961). Social Dominance in Inbred Mouse Strains. Nature *191*, 474–476.

Moy, S.S., Nadler, J.J., Perez, A., Barbaro, R.P., Johns, J.M., Magnuson, T.R., Piven, J., and Crawley, J.N. (2004). Sociability and preference for social novelty in five inbred strains: an approach to assess autistic-like behavior in mice. Genes Brain Behav. *3*, 287–302.

Sawamoto, K., Nakao, N., Kobayashi, K., Matsushita, N., Takahashi, H., Kakishita, K., Yamamoto, A., Yoshizaki, T., Terashima, T., Murakami, F., et al. (2001). Visualization, direct isolation, and transplantation of midbrain dopaminergic neurons. Proc. Natl. Acad. Sci. *98*, 6423–6428.

Sparta, D.R., Stamatakis, A.M., Phillips, J.L., Hovelsø, N., van Zessen, R., and Stuber, G.D. (2012). Construction of implantable optical fibers for long-term optogenetic manipulation of neural circuits. Nat. Protoc. *7*, 12–23.

Tsai, H.-C., Zhang, F., Adamantidis, A., Stuber, G.D., Bonci, A., de Lecea, L., and Deisseroth, K. (2009). Phasic firing in dopaminergic neurons is sufficient for behavioral conditioning. Science *324*, 1080–1084.

Vicini, S., Wang, J.F., Li, J.H., Zhu, W.J., Wang, Y.H., Luo, J.H., Wolfe, B.B., and Grayson, D.R. (1998). Functional and pharmacological differences between recombinant N-methyl-D-aspartate receptors. J. Neurophysiol. *79*, 555–566.

Vong, L., Ye, C., Yang, Z., Choi, B., Chua, S., and Lowell, B.B. (2011). Leptin Action on GABAergic Neurons Prevents Obesity and Reduces Inhibitory Tone to POMC Neurons. Neuron *71*, 142–154.

Wang, F., Zhu, J., Zhu, H., Zhang, Q., Lin, Z., and Hu, H. (2011). Bidirectional Control of Social Hierarchy by Synaptic Efficacy in Medial Prefrontal Cortex. Science *334*, 693–697.

Zhao, S., Maxwell, S., Jimenez-Beristain, A., Vives, J., Kuehner, E., Zhao, J., O'Brien, C., de Felipe, C., Semina, E., and Li, M. (2004). Generation of embryonic stem cells and transgenic mice expressing green fluorescence protein in midbrain dopaminergic neurons. Eur. J. Neurosci. *19*, 1133–1140.
